# Risk Diagrams Based on Primary Care Electronic Medical Records and Linked Real-Time PCR Data to Monitor Local COVID-19 Outbreaks During the Summer 2020: A Prospective Study Including 7,671,862 People in Catalonia

**DOI:** 10.3389/fpubh.2021.693956

**Published:** 2021-07-05

**Authors:** Marti Catala, Ermengol Coma, Sergio Alonso, Enrique Álvarez-Lacalle, Silvia Cordomi, Daniel López, Francesc Fina, Manuel Medina-Peralta, Clara Prats, Daniel Prieto-Alhambra

**Affiliations:** ^1^Computational Biology and Complex Systems (BIOCOM-SC), Department of Physics, Universitat Politècnica de Catalunya, Castelldefels, Spain; ^2^Comparative Medicine and Bioimage Centre of Catalonia (CMCiB), Germans Trias i Pujol Research Institute (IGTP), Badalona, Spain; ^3^Sistemes d'Informació dels Serveis d'Atenció Primària (SISAP), Institut Català de la Salut (ICS), Barcelona, Spain; ^4^Direcció d'Estratègia i Qualitat, Institut Català de la Salut, Barcelona, Spain; ^5^Centre for Statistics in Medicine, NDORMS, University of Oxford, Oxford, United Kingdom

**Keywords:** COVID-19, public health, surveillance, outbreak detection, sentinel

## Abstract

Monitoring transmission is a prerequisite for containing COVID-19. We report on effective potential growth (EPG) as a novel measure for the early identification of local outbreaks based on primary care electronic medical records (EMR) and PCR-confirmed cases. Secondly, we studied whether increasing EPG precedes local hospital and intensive care (ICU) admissions and mortality. Population-based cohort including all Catalan citizens' PCR tests, hospitalization, intensive care (ICU) and mortality between 1/07/2020 and 13/09/2020; linked EMR covering 88.6% of the Catalan population was obtained. Nursing home residents were excluded. COVID-19 counts were ascertained based on EMR and PCRs separately. Weekly empirical propagation (ρ_7_) and 14-day cumulative incidence (A_14_) and 95% confidence intervals were estimated at care management area (CMA) level, and combined as EPG = ρ_7_ × A_14_. Overall, 7,607,201 and 6,798,994 people in 43 CMAs were included for PCR and EMR measures, respectively. A14, ρ_7_, and EPG increased in numerous CMAs during summer 2020. EMR identified 2.70-fold more cases than PCRs, with similar trends, a median (interquartile range) 2 (1) days earlier, and better precision. Upticks in EPG preceded increases in local hospital admissions, ICU occupancy, and mortality. Increasing EPG identified localized outbreaks in Catalonia, and preceded local hospital and ICU admissions and subsequent mortality. EMRs provided similar estimates to PCR, but some days earlier and with better precision. EPG is a useful tool for the monitoring of community transmission and for the early identification of COVID-19 local outbreaks.

## Introduction

Coronavirus disease 2019 (COVID-19) caused by SARS-CoV-2 started as an outbreak in Wuhan (China) late December and quickly evolved into a worldwide pandemic. The first cases in Europe were reported in France on January 24 2020, spreading to neighboring countries within weeks (1). The first case reported in Spain was seen on February 1st in the Canary islands, and in the peninsula just over 3 weeks later, on February 25th. Despite universal healthcare and other advantages, Spain was heavily hit by COVID-19 morbimortality in March to May, motivating public health experts to ask for an independent evaluation of the national response to the pandemic (2).

During the first wave of COVID-19, Spain lacked the capacity to test all cases and contacts, and PCR confirmation was only required when patients were admitted to hospital or if they were healthcare workers. In a recent study, only 38.5% of clinical COVID-19 cases diagnosed between March 1 and April 24 2020 received a RT-PCR test in Catalonia (3). Subsequently, on May 11th, Spanish authorities advocated that all clinical diagnoses of COVID-19 should have a test, and the number of RT-PCR performed increased dramatically since then in an attempt to reduce the test positivity ratio (4, 5). In Catalonia, official figures show that testing rates increased from 551 RT-PCR tests per week per 100,000 people the first week of June to 1,352 the first week of September.

In parallel to testing, primary care electronic medical records (EMR) have been previously used as a surveillance system for the monitoring of influenza epidemics (6), and could be useful to complement testing data. During the first months of the pandemic, EMR-based measures detected an excess of flu cases weeks before the first official cases of COVID-19 in Catalonia were reported (7), demonstrating the potential usefulness of EMR-based surveillance systems for the timely detection of COVID-19 outbreaks.

Recent analyses of lessons learnt from easing COVID-19 restrictions have highlighted the need for measures of community transmission and actionable indicators as part of successful exit strategies (8). In this framework, it has revealed essential to count on a robust system to detect local outbreaks and determine the epidemiological risk at different spatial levels. Besides the traditional methods, new approaches for the detection of anomalies in surveillance data have been developed over the last years, but their practical use is still limited (9). Effective potential growth (EPG) and risk diagrams have been used to visualize the dynamics of the pandemic at a local level in Spain and included in weekly reports by the DG CONNECT of the European Commission (4, 5, 10). By combining 14-day cumulative incidence (A_14_) as an indicator of active infections and empirical reproduction number (ρ_7_) as a measure of propagation trends, EPGs have been used to monitor the status of the pandemic, and to inform public health actions by the Catalan government (4, 5, 11, 12). Risk diagrams built with RT-PCR positive records have been used for the monitoring of de-confinement in April-June, and proposed as a useful tool for the detection of local outbreaks during summer 2020.

We aimed to describe EPG and risk diagrams based on primary care EMR as well as on PCR-confirmed cases as novel measures to monitor COVID-19 local outbreaks. Secondly, we set out to demonstrate how increases in EPG precede local COVID-19 hospital and intensive care unit (ICU) admissions, and subsequent related mortality.

## Methods

### Design and Data Sources

We performed a prospective population-based study. Data were obtained from the official repository of reverse transcriptase polymerase chain reaction (RT-PCR) tests for severe acute respiratory syndrome coronavirus 2 (SARS-CoV-2) linked to hospital admissions, intensive care units (ICU) and mortality registries, covering the whole population of Catalonia (>7.6 million people). In addition, a data series of the linked primary care electronic medical records (EMR) was obtained, covering a representative >6.7 million people (>88% of the total population). These data are recorded using the same EMR software called ECAP, which includes clinical diagnoses, measurements, prescriptions, and health outcomes. ECAP data have been previously validated (13, 14), and used extensively for research on COVID-19 as well as on other conditions (7, 15). The different databases involved in the extractions are linked between them by the anonymized individual identifier of the patients, which guarantees the validity of such extractions and the comparability between the obtained data series, since all of them correspond to a common cohort of patients. All the data analyzed for this report are available online at https://github.com/catalamarti/EMRandPCR_Catalonia.

### Participants

We included the whole source population (the whole population of Catalonia) alive during the study period (1st July to 13th September 2020) for measures based on RT-PCR, hospital admissions, ICU, and mortality. Analyses based on EMR were based on the >6.7 million people with linked EMR available. We excluded nursing-home residents from all analyses.

### Case Ascertainment

To perform our analysis, we used two different case definitions, based on positive RT-PCR and EMR diagnoses, respectively. PCR cases are based on a series of positive RT-PCR cases where we attribute the date of case identification as the earliest of the following: collection of a positive RT-PCR test or clinical diagnosis (where available). EMR cases are identified based on primary care clinical diagnoses of COVID-19. Their index date is the day when a general practitioner recorded one of the eligible codes in the patient's EMR. Eligible codes were based on the International Classification of Diseases 10th revision (ICD-10) and included any of the following: U07.1, B34.2, B97.21, B97.29, J12.81 and J12.89.

### Study Period

We used data from 1st July to 13th September 2020 divided into two study periods. We separated these periods to allow prospective follow up to calculate the delay in real-world conditions. The two periods established were: (1) from 27th August to 13rd September to calculate the delay between series; and (2) from 1st July to August 31st for the rest of the measures.

### Epidemiological Measures

All the epidemiological measures described below were estimated at a Care Management Area (CMA) level. CMAs are the smallest healthcare provision unit in Catalonia, and include primary care practices and geographically related local hospitals. There is a total of 43 CMAs in Catalonia, including large urban areas of Barcelona and surrounding areas, as well as smaller rural areas. The epidemiological measures below were estimated based on PCR cases and on EMR counts separately. Formuli are available in Supplementary Equations 1–4.

- n_7_: Average case count based on the last 7 days
- A_14_: 14-day cumulative incidence per 100,000 inhabitants
- ρ_7_: empirical reproduction number. Indicative of average number of contagions by each infectious individual. See formula in Supplementary Equation 3.
- EPG (Effective Potential Growth). This novel index is calculated as the product between A_14_ and ρ_7_ (EPG=A_14_·ρ_7_). Given that A_14_ is a rough measurement of the contagious population (active cases) and that ρ_7_ is the mean number of new cases per contagious individual, EPG can be interpreted as indicative of the level of new cases to be managed the subsequent fortnight.
- Outbreak risk levels: thresholds of EPG have been pre-specified for Catalonia based on local testing and healthcare capacity, and detailed in European reports (16). The proposed thresholds, from very low to very high risk, are detailed in Supplementary Information.

### Statistical Analyses

Data on EMR and PCR-based cases over time up to a given day change when data from an extra day is added. The new report on a given day not only adds data for the new day but also increases the count in previous days. Eighteen consecutive daily series were used to perform an analysis of these delays, each of them containing the whole historical series until August 26th. Each of the eighteen individual series were extracted by adding the information of one extra day between August 27th and September 13th. We compared each series with the previous one to compute where new cases were added. A total of 17 comparisons were carried out.

Eighteen consecutive daily series were used to perform delay analysis, each of them containing the whole historical series at that moment. Individual series were extracted between August 27th and September 13th. Each consecutive series included data from a new day but also corrected using case counts from previous ones. We compared each series with the previous one to compute where new cases were added. A total of 17 comparisons were carried out.

For each comparison we estimated how many cases (PCR and EMR) separately, added to that day's point (0 days back), previous day counts, etc. Cases added more than 30 days back were excluded from the analysis. Therefore, the total amount of analyzed records is 17,016 PCR and 43,972 EMR. We computed mean, median and 5 and 95% percentiles of the observed delay between PCR- and EMR-based cases at CMA level. Daily measures based on PCR and EMR case counts were reported separately, with confidence intervals estimated based on a binomial probability multiplied by population size. Binomial confidence intervals were computed using the Clopper-Pearson method (17). Confidence intervals for n_7_, A_14_, ρ_7_, and EPG were computed using propagation uncertainty (see equations in Supplementary Equations 6–9).

Delays between EPG series and other series (e.g., hospitalizations series) were determined by comparing the correlation between series displaced. We subsequently displaced the compared series a certain delay that was increased by 1 day each time. Then, we assessed directly the correlation between displaced series. Given the closely linked nature of the data series, which prevents the influence of complicated confounders, the delay that provided the maximum correlation was established as the lag between series, as seen in Supplementary Figure 183. Full method description can be found in (18).

The proportionality coefficient between PCR and EMR series was computed as the ratio between both case counts during the study period (1 July−31st August). This quotient was computed for Catalonia overall and for each CMA. Ninety five percent confidence intervals were estimated using propagation of uncertainty theory (see Supplementary Equation 5). We used the henceforth derived overall and CMA-specific proportionality coefficients to correct EMR-based estimates of epidemiological indicators (n_7_, A_14_, ρ_7_, and EPG). Daily EPGs were calculated based on PCR and EMR-based estimates separately, and categorized according to the pre-defined thresholds above. We therefore obtained two EPG values and two risk categories per day, one based on PCR and one on EMR data. We considered both in agreement when (1) their confidence intervals overlapped, or (2) they resulted in the same risk category. Such concordance was evaluated for each CMA overall, and stratified by risk category.

Only aggregated data was used for these analyses. No patient level data was requested or analyzed. All of these data are freely available at https://github.com/catalamarti/EMRandPCR_Catalonia. All calculations were performed using Matlab 2020b. The analytical codes are provided at the same link.

## Results

A total of 43 CMAs were analyzed, covering a total of 7,671,862 Catalan residents. Of these, 7,607,201 (99.2%) people were included for the analyses of PCR-based EPGs, and 6,798,994 (88.6%) for EMR-based analyses. CMAs varied in population size, from 3,439 people in Alta Ribagorça to 520,609 in the district of Barcelona Esquerra. Socio-demographics were also different, with average (standard deviation) age ranging from 40.8 (22.5) to 47.1 (24) years old in Gironès Nord and Altebrat respectively; and % of women ranging from 48.9 to 53.5% in Gironès Sud and Barcelona Dreta. Socio-economic status also varied, from 0 to 61.2% in the lowest quintile (i.e., most deprived) for Alta Ribagorça and Selva Marítima, respectively. Details for the overall population as assessed on 16th October, overall and at CMA level are reported in Supplementary Table 1. Baseline characteristics for those included in EMR-based analyses are detailed in Supplementary Table 2.

Overall, 49,666 PCR-confirmed cases were observed in Catalonia in the study period, corresponding to a cumulative incidence of 652.8/100,000 people between July 1st and August 31st 2020. Great heterogeneity existed in the number of PCR+ cases identified per health region, ranging from 12 cases in Alta Ribagorça to 7,096 in Lleida. Similarly, local estimates of cumulative incidence varied greatly in the same period, between 193.8/100,000 in Bergueda and 1975.8/100,000 in Lleida. The number of EMR-registered cases was overall 2.70 fold higher, with a total of 134,071 in Catalonia in the same period, equivalent to a cumulative incidence of 1,762.2/100,000. Consistent with PCR data, Alta Ribagorça and Lleida were the health regions with the lowest and highest numbers of EMR-based cases, with a total of 64 and 13,868 cases, respectively. Local (CMA-level) EMR-based cumulative incidence estimates ranged between 106.3/100,000 in Baix Penedes to 3,861.4/100,000 in Lleida. The number of EMR-suspected cases exceeded that of PCR-confirmed in all health regions except Baix Penedes (93 EMR vs. 249 PCR+), with a ratio of EMR/PCR ranging -excluding Baix Penedes- from 1.39 (Alt Urgell) to 8.81 (Baix Emporda). Supplementary Table 3 reports all these measures at the health region level and for the whole of Catalonia.

EMR preceded PCR notification by a mean (median) of 2.8 (2) days, with a range per CMA from 2.0 (2) days in Ripolles to 4.4 (3) in Lleida. Supplementary Figure 5 depicts the delay between PCR and EMR-based cases according to fraction of reported cases. As shown, EMR-based diagnoses were recorded most often on the same day of the case, whilst PCR confirmation was delayed by a median (mean) of 2 (3.2) days. Exact figures for each CMA are reported in Supplementary Table 4.

The overall number of 7-day average daily new cases (n_7_), empirical propagation (ρ_7_), 14-day cumulative incidence (A_14_) and the resulting effective potential growth index (EPG) are depicted over time for the whole of Catalonia in Figure 1. A_14_ and EPG increased rapidly in July to then plateau in early August to start slowly increasing again in the last 10 days of that same month. These ran in parallel with the opposite trend for ρ_7_ measures, that fluctuated around 1.5 in July to then decrease to close to 1 during August. EMR and PCR-based estimates approximated each other closely (see Supplementary Figure 6). CMA-specific figures are provided in Supplementary Figures 7–50.

**Figure 1:**
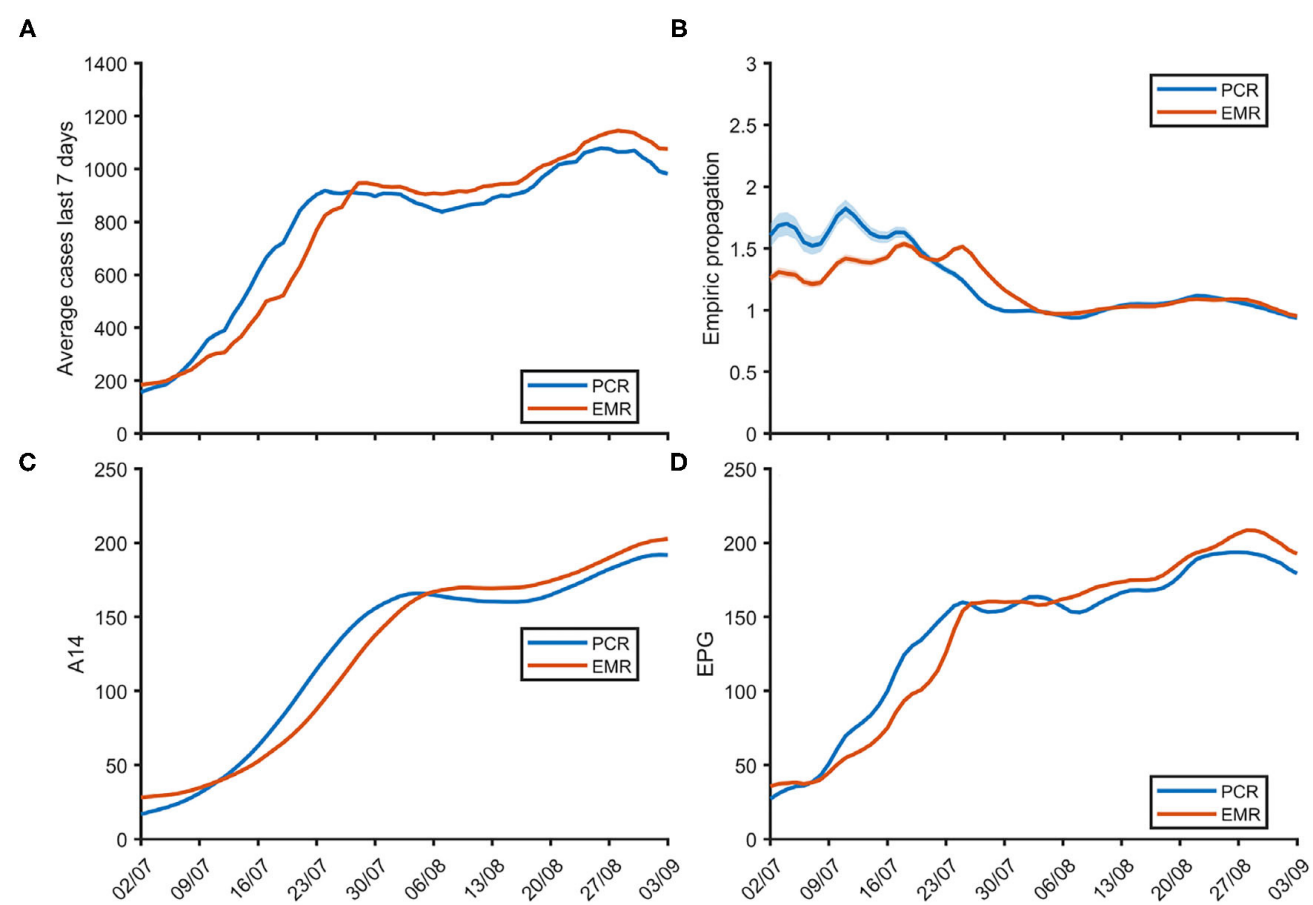
Weekly average of daily new cases n_7_ **(A)**, empirical propagation ρ_7_ **(B)**, 14-day cumulative incidence A_14_ **(C)** and effective potential growth EPG **(D)** over time for the whole of Catalonia.

Both EMR and PCR-based EPGs (and their confidence intervals) overlapped in 88% of cases: 86% for periods classified as very low risk, 81% for low risk, 85% for moderate risk, 93% for high risk, and 93% for periods of very high risk. CMA-specific EMR and PCR-based measures are reported in Supplementary Table 5 and depicted in Supplementary Figures 51–94.

**Figure 2:**
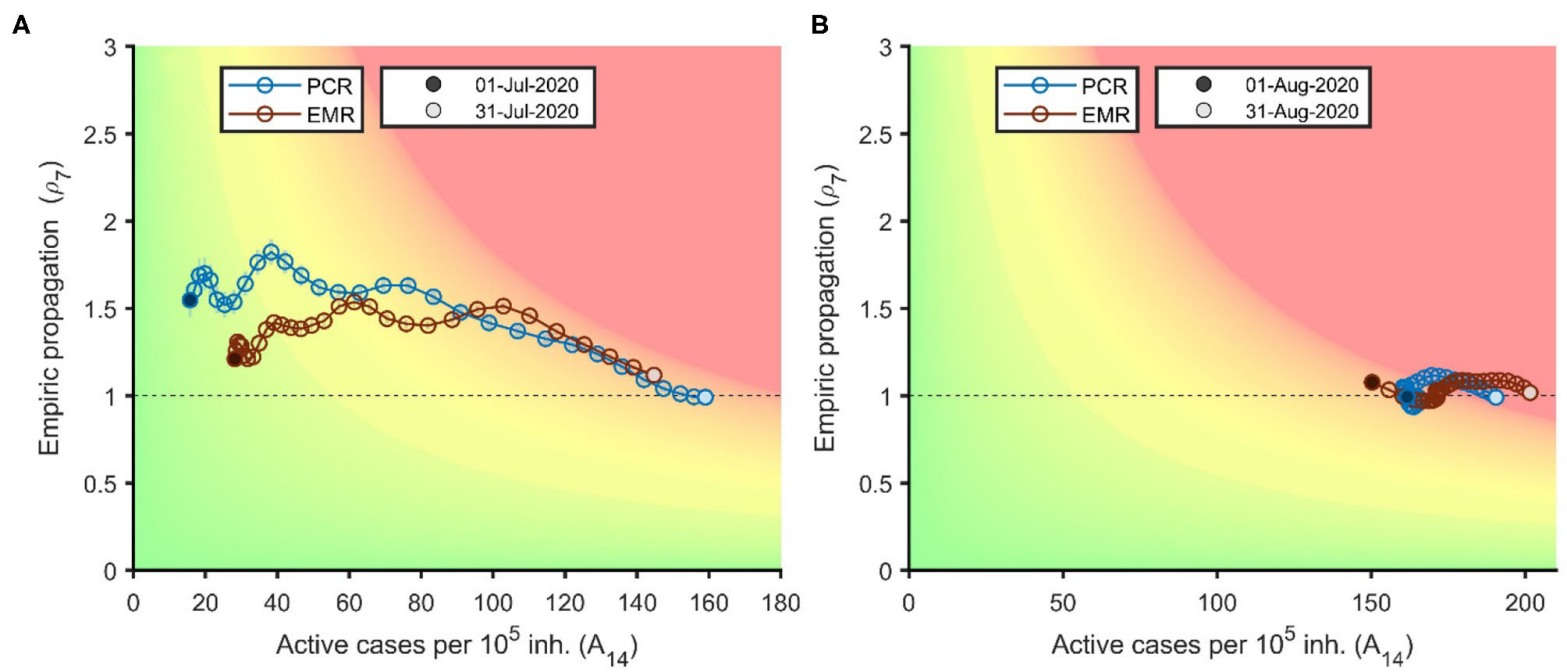
Risk diagram for the evolution of the COVID-19 pandemic in Catalonia based on EMR (red) and on PCR (blue) cases for the month of July **(A)** and August 2020 **(B)**.

Risk diagrams for Catalonia in July and August 2020 are shown in Figure 2, with colors denoting pre-specified outbreak risk categories, from green (very low risk) to red (very high risk). The diagram shows how a ρ_7_ above 1–1.5 (Y axis) in early July led to a rapid increase in A_14_, from low figures (20–30/100,000) at the beginning to 140–160/100,000 by the end of the month. Late July and August saw a reduction in ρ_7_ to values close to 1, stable during the whole month of August. A_14_ estimates however continued to increase although at a slower pace than in July, going from around 150 at the beginning of August to around 200/100,000 by the end of the month.

Figure 3 shows the illustrative example of Lleida, the first CMA to suffer a local outbreak in the summer, with both ρ_7_ and A_14_ increasing (between 1.5 and 2 and around 150/100,000, respectively) already at the beginning of July. Strict restrictions drove ρ_7_ downwards to values below 1 by the end of July. This resulted in a down and left turn in the diagram due to a rapid decrease in ρ_7_ and A_14_ in August. This is shown as a steep left shift across the X axis, with ρ_7_ ~0.7–0.8 during most of the month. Restrictions were then lifted followed by an increase in ρ_7_ and an upwards-right loop, with increased ρ_7_ and A_14_ in the last days of the month. Similar risk diagrams for all the CMAs are available in Supplementary Figures 95–138. Less populated areas demonstrate gains in precision when EMR-based EPGs are compared to PCR-based ones, as shown by narrower confidence intervals (shaded bars).

**Figure 3:**
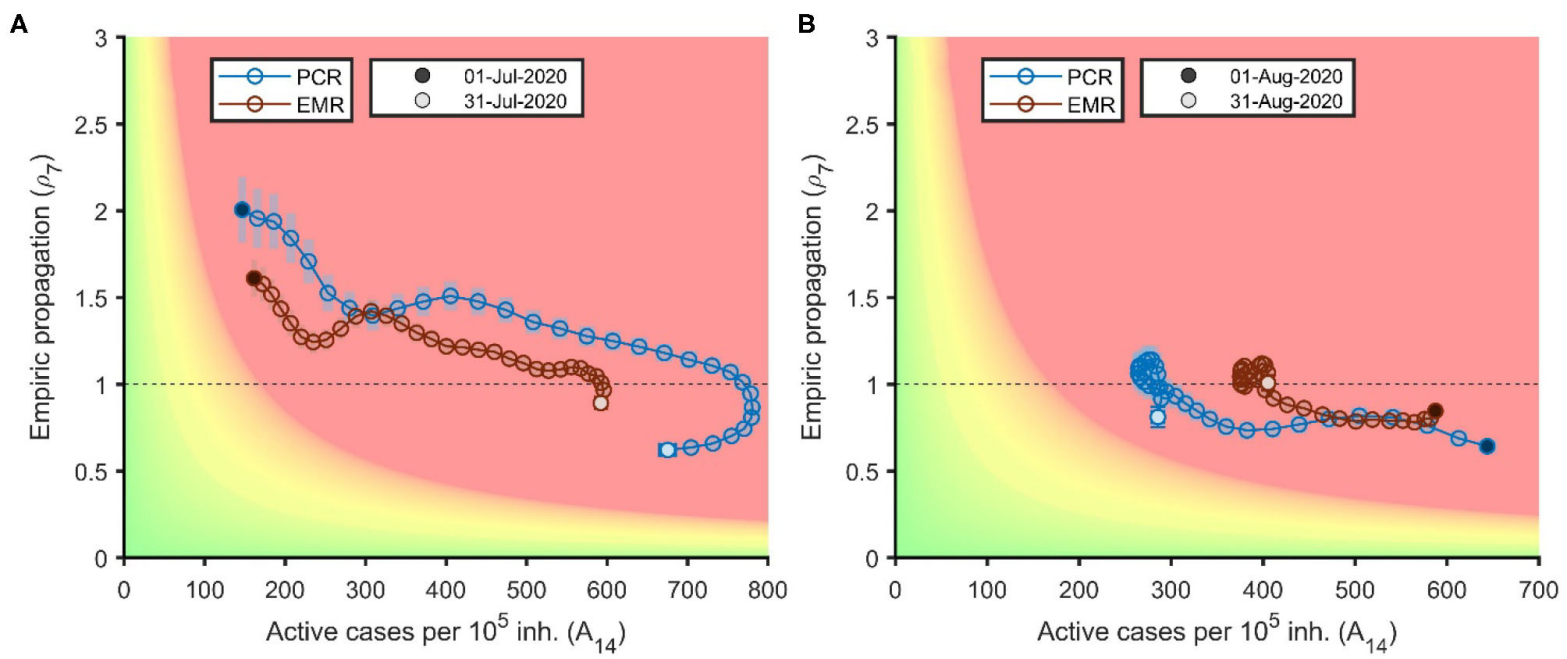
Risk diagrams for the health region of Lleida in July **(A)** and August 2020 **(B)**.

Figure 4 demonstrates how an increase in EPG in Catalonia preceded subsequent rises in hospital admissions, ICU occupancy, and COVID-19 mortality in July and August 2020. Whilst EPGs started increasing in the first week of July, hospital admissions started to grow a week later, followed by ICU admissions a few days later, and mortality increasing subsequently. According to the correlation analysis, the lags between increases in EPG and hospitalizations, ICU and mortality were 4, 10, and 10 days if using the PCR series and 2, 7, and 9 days if using the EMR series, respectively. Similar associations were observed at the local level for each of the CMAs (see Supplementary Figures 139–182).

**Figure 4:**
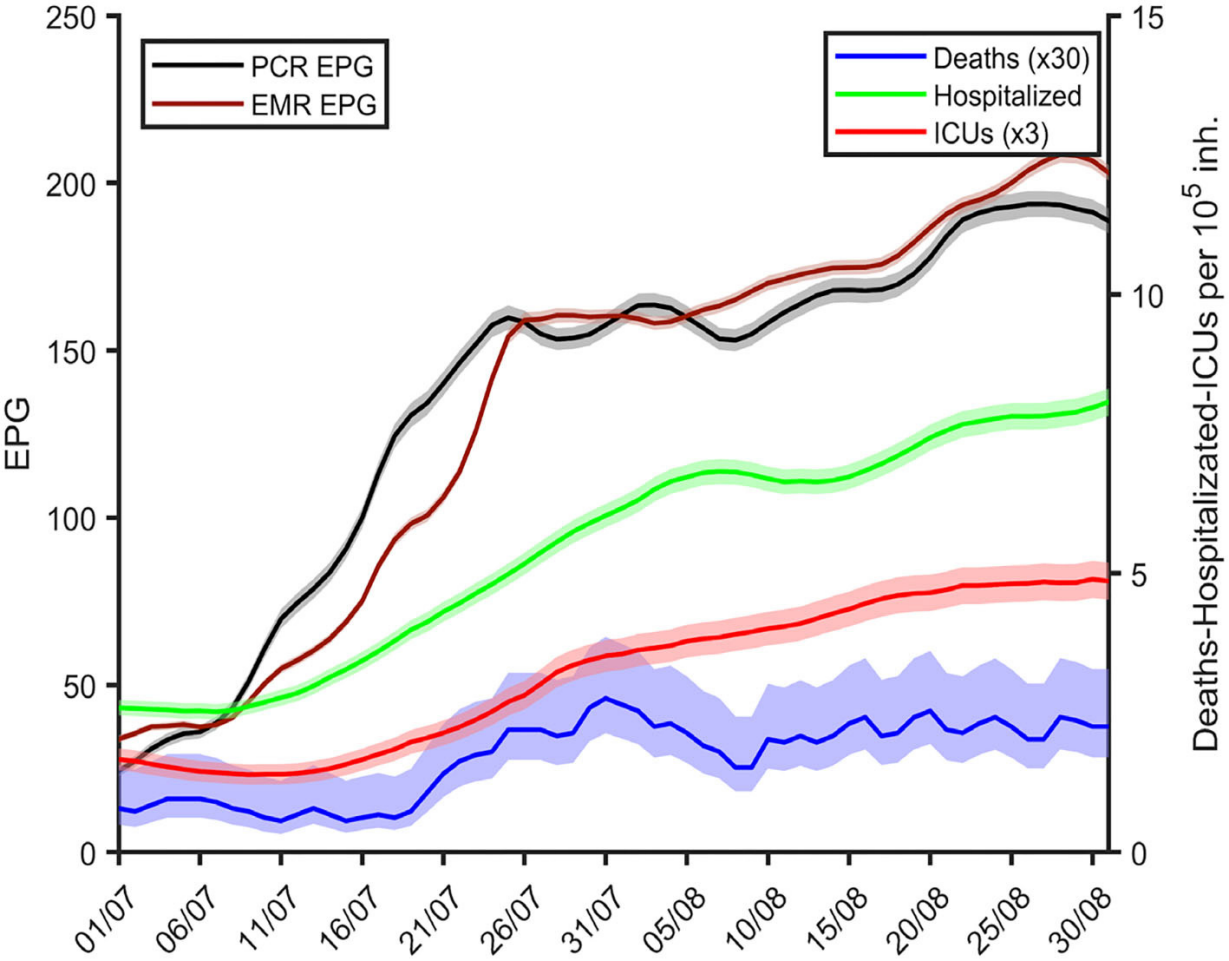
Daily measures of EMR (maroon) and PCR-based EPGs (black), number of hospitalizations (green), ICU occupancy (red), and mortality (blue) in all Catalonia over time from late June until the end of August 2020. Hospitalizations, ICU and mortality are averaged over the previous 7 day period.

## Discussion

We report ρ_7_, A_14_, and EPG measures as early markers of local outbreaks and subsequent increases in hospital admissions, ICU bed occupancy and mortality in Catalonia health regions (CMAs) during the summer of 2020. Estimates were based on PCR test data for 7.6 million people (>99% of the population), with equivalent measures derived based on linked primary care EMR covering almost 90% of the population. We identified over 49,000 PCR-confirmed cases of COVID-19 between July 1st and August 31st 2020 in Catalonia, equivalent to an overall A_14_ of almost 700/100,000. Numbers of cases and cumulative incidence varied greatly among the 43 Catalan CMAs, with A_14_ ranging between just below 200 and over 2,000 in the same study period. EMR-based measures showed similar trends but with an almost 3-fold higher A_14_ of almost 1,900/100,000 in the whole of Catalonia, and localized outbreaks reaching to over 4,000/100,000 in the most affected CMAs.

EPG identified CMAs of growing concern and was used to target local public health measures including mobility restrictions and curfews. With over 80 countries having gone through lockdowns in the first half of 2020, and others establishing new similar restrictions in the autumn of the same year, it is essential that key principles are followed to ensure successful exiting from such restrictions (19). Having the ability to monitor community transmission has been proposed as one of the pillars for successfully exiting restrictions (8, 20).

Both ρ_7_, and A_14_ are complementary measures, and similar ones (R_t_ and A_7_) have been used by countries like Germany to trigger restrictions after the identification of local outbreaks (21). The here proposed novel EPG combines both and provides good predictive measures for healthcare system strains. As a product of ρ_7_, and A_14_, EPG relates to increases in any of both estimates, and amplifies even further when both increase, thus becoming a useful proxy to identify new outbreaks.

In many countries, the first indicator that is used as an alarm sign for the application of certain measures is 7 or 14-day cumulative incidence. This indicator is based on the last 1 or 2 weeks dynamics, thus corresponding to a past situation. EPG is based on past data but points to future dynamics. Therefore, its use for the setting of primary thresholds that may suggest the application or release of control measures include, to certain extent, not only the last days' situation but also the short-term expected trend. The merging of a past situation indicator and a future short-term trend in a single index provides a more robust way of assessing the epidemiological situation. Our data demonstrate how EPG preceded increases in local hospital admissions, ICU occupancy and COVID-19 mortality by 4, 10, and 10 days if using the PCR series and 2, 7, and 9 if using the EMR series, respectively, demonstrating the usefulness of EPG as an epidemiological marker of local outbreaks. Further to the epidemiological usefulness of EPGs, their depiction in the form of risk diagrams is useful to facilitate communication of outbreak risks and improve engagement with society. The use of pre-specified thresholds and related actions has been highlighted as a useful tool to improve compliance with any proposed public health restrictions (8).

The simplicity of this indicator together with the robustness of its application as an early warning system makes the EPG a reliable index to incorporate in any public health setting, when more sophisticated modeling infrastructures are difficult to implement. Nevertheless, even though the EPG incorporates the future trend (ρ_7_) in addition to the past situation (A_14_), it should not be considered as a forecasting index, but a way to assess the degree of epidemiological risk in a certain region.

In addition to PCR-confirmed case counts, EMR-based epidemiological measures can be obtained from local primary care practices. Correlation with PCR ~90%, whilst providing improved accuracy (less uncertainty) and earlier estimates by 1–4 days. In addition to these advantages, EMR-based measures can be obtained in real time. Real-time R measures have been successfully used in some countries like Hong Kong to control the pandemic (22). EMR data has been used in the past and showed to be able to identify COVID-19 outbreaks before testing capacity was developed in Catalonia (7). Our data suggest that EMR-based EPGs can be added to the existing toolbox of measures useful to control COVID-19 community transmission (23).

Our study has both limitations and strengths. First, EMR-based measures are based on clinical diagnoses, not always confirmed with gold standard tests like RT-PCRs. However, we demonstrate that correlation in the derived epidemiological estimates of ρ_7_ and A_14_ is high and close to 90%. Secondly, the proposed EPG thresholds were pre-specified based on local testing and healthcare capacity, limiting their generalizability. Similar thresholds have been used in other countries like Germany (24), but further validation is needed to adapt the proposed thresholds to parts of the world with different testing and healthcare systems. Our study also has strengths. We accessed a unique EMR dataset with previously demonstrated quality and completeness, and enriched with linkage to RT-PCR testing, hospital occupancy, and mortality registries. Access to real time EMR and laboratory data enabled the analyses reported here.

## Conclusions

EPG and related risk diagrams are useful tools to identify local outbreaks, and to communicate pre-specified thresholds for local public health targeted action. Increases in local EPG precede hospital admissions, ICU occupancy and mortality by 4, 10, and 10 days if using the PCR series and 2, 7, and 9 if using the EMR series, respectively. Besides, the use of EMR case counts resulted in better accuracy and 1–3 days gains in the estimation of COVID-19 epidemiological measures. Available as real-time measures, EMR-based EPG can help the early targeting of local outbreaks before RT-PCR counts confirm them.

## Data Availability Statement

The datasets presented in this study can be found in online repositories. The names of the repository/repositories and accession number(s) can be found below: < https://github.com/catalamarti/EMRandPCR_Catalonia>.

## Ethics Statement

Ethical review and approval was not required for the study on human participants in accordance with the local legislation and institutional requirements. Written informed consent from the participants' legal guardian/next of kin was not required to participate in this study in accordance with the national legislation and the institutional requirements.

## Author Contributions

EC, SC, FF, and MM-P performed data curation and aggregation. SA, DL, EA-L, MC, and CP developed the indicators and tools used for data analysis. MC and EC lead data and statistical analysis and prepared the figures. DP-A, CP, and MM-P designed and lead the research. DP-A, EC, MC, and CP wrote the first drafte of the manuscript. All authors have read, reviewed, corrected and approved the manuscript.

## Conflict of Interest

DP-A department has received consultancy fees from UCB Biopharma. DP-A department has received speaker fees from AMGEN. Janssen, on behalf of IMI-funded EHDEN and EMIF consortiums, and Synapse Management Partners have supported training programmes organised by DP-A department and open for external participants. CP has received consultancy fees from Janssen. None of these relationships has directly funded this study. The remaining authors declare that the research was conducted in the absence of any commercial or financial relationships that could be construed as a potential conflict of interest.
