# Supplementary material for: Risk Diagrams Based on Primary Care Electronic Medical Records and Linked Real-Time PCR Data to Monitor Local COVID-19 Outbreaks During the Summer 2020: A Prospective Study Including 7,671,862 People in Catalonia
Source: Front Public Health. 2021 Jul 5;9:693956. doi: 10.3389/fpubh.2021.693956 (PMC8287173; doi:10.3389/fpubh.2021.693956)
Supplement: Supplementary file 1 [file Data_Sheet_1.PDF]

Risk diagrams based on primary care electronic medical records and linked real-time PCR data to monitor local COVID-19 outbreaks during the summer 2020: a prospective cohort study including 7,671,862 people in Catalonia

Supplementary Information

Authors: Marti Catala, Ermengol Coma, Sergio Alonso, Enrique Alvarez, Silvia Cordomi, Daniel Lopez-Codina, Francesc Fina, Manuel Medina-Peralta, Clara Prats*, Daniel Prieto-Alhambra

* Corresponding author: clara.prats@upc.edu

Methodological notes

Detail on the calculation of the proposed epidemiological measures

- n_7: Average case count based on the last 7 days. Series of daily new cases using a 7-day moving average. This measure was used for PCR-, EMR-based cas deaths, hospitalizations, ICU, and mortality.

$$n_7(t) = \frac{1}{7}\sum_{t-6}^{t} n(t) \qquad (1)$$

- A_{14}: 14-day cumulative incidence. This value is assessed as the sum of the cases diagnosed last 14 days per 100,000 inhabitants. Calculated using n_7 and used as an indicator of infectious cases in the community:

$$A_{14}(t) = \frac{10^5}{population}\sum_{t-13}^{t} n_7(t) \qquad (2)$$

- ρ_7: empirical reproduction number. Indicative of average number of contagions by each infectious individual, it is calculated based on n_7 as follows:

$$\rho_7(t) = \frac{n_7(t) + n_7(t-1) + n_7(t-2)}{n_7(t-5) + n_7(t-6) + n_7(t-7)} \qquad (3)$$

- EPG (Effective Potential Growth). This novel index is calculated as the product between A_{14} and ρ_7. Given that A_{14} is a rough measurement of the contagious population (active cases) and that ρ_7 is the mean number of new cases per contagious individual, EPG can be interpreted as an estimation of the number of new cases expected for the subsequent fortnight.

$$EPG(t) = \rho_7(t) \cdot A_{14}(t) \qquad (4)$$

- Outbreak risk levels: thresholds of EPG have been pre-specified for Catalonia based on local testing and healthcare capacity, and detailed in European reports [ref]. The proposed thresholds are as follows:
    - EPG<30: very low risk. Test and trace capacity is capable of identifying all cases and contacting all controls.

- EPG between 30 and <70: low risk level. The test and trace capacity is enough to control the epidemic almost case by case, but personal preventive measures (masks, distance, hygiene, ventilation) must be reinforced.
- EPG between 70 and <100: intermediate risk level. The test and trace capacity could be close to saturation in some primary care centers. The situation requires close monitoring to detect any local hotspot and redirect resources, if necessary.
- EPG between 100 and <200: high risk level. The test and trace capacity is close to saturation, and soft supplementary control measures must be assessed and implemented.
- EPG≥200: very high risk level. The test and trace capacity could be overcome. Harder supplementary control measures must be assessed and implemented.

Propagation of errors

Experimental measures of the number of cases gives us an uncertainty interval $\sigma_n(t)$ for each measure on a given day $n(t)$. Uncertainty of other epidemiological measures can be computed from propagation of errors theory. Given a magnitude z that is a function of two other variables x and y we can compute the uncertainty in z, σ_z, as a function of uncertainty in x, σ_x, and y, σ_y:

$$z = f(x, y) \rightarrow \sigma_z^2 = \left(\frac{\partial f}{\partial x}\right)^2 \sigma_x^2 + \left(\frac{\partial f}{\partial y}\right)^2 \sigma_y^2. \quad (5)$$

The epidemiological indicators which we are interested in are: average case count based on last 7 days (n_7), 14-day cumulative incidence (A_{14}), empirical reproduction number (ρ_7) and Effective Potential Growth (EPG). Using equation 5 there can be computed uncertainty intervals for different epidemiological indices:

$$\sigma_{n_7}(t) = \frac{1}{7}\sqrt{\sum_{t-6}^{t} \sigma_n(t)^2}, \quad (6)$$

$$\sigma_{A_{14}}(t) = \frac{10^5}{population}\sqrt{\sum_{t-13}^{t} \sigma_{n_7}(t)^2}, \quad (7)$$

$$\sigma_{\rho_7}(t) = \frac{1}{n_7(t-5) + n_7(t-6) + n_7(t-7)}\sqrt{\sum_{t-2}^{t} \sigma_{n_7}(t)^2 + {\rho_7}^2 \sum_{t-7}^{t-5} \sigma_{n_7}(t)^2}, \quad (8)$$

$$\sigma_{EPG}(t) = \sqrt{A_{14}(t)^2 \cdot \sigma_{\rho_7}(t)^2 + \rho_7(t)^2 \cdot \sigma_{A_{14}}(t)^2}. \quad (9)$$

Table 1. Baseline characteristics of study population for PCR analyses on 16th October 2020, overall and stratified by CMA

CMA	N	N in nursing home (excluded)	Age (Mean)	Age (SD)	% of women
Alt Camp i Conca de Barberà	61356	624	43.0	23.1	49.1
Alt Empordà	138684	852	42.1	22.9	50.2
Alt Maresme	118610	911	43.1	23.0	50.3
Alt Penedès	98020	735	41.9	22.9	49.9
Alt Urgell	20071	198	45.4	23.4	50.3
Alta Ribagorça	3482	40	44.4	23.0	49.2
Altebrat	32303	335	47.1	24.0	49.0
Anoia	113559	869	42.1	23.1	49.8
Aran	9355	43	42.0	21.3	49.9
Bages i Solsonès	212683	2308	42.9	23.3	50.2
Baix Camp i Priorat	201625	2147	41.8	22.9	50.6
Baix Ebre	93154	715	44.3	23.6	49.5
Baix Empordà	128254	230	42.8	23.1	50.3
Baix Llobregat Centre i Fontsanta - L'H N	434994	2777	42.3	22.8	51.4
Baix Llobregat Litoral i Sant	127020	761	42.2	22.6	50.9
Baix Llobregat Litoral i Viladecans	188874	1185	41.4	22.3	50.8
Baix Llobregat Nord	160554	1269	40.8	22.3	49.5
Baix Montseny	56590	448	41.7	22.6	50.1
Baix Penedès	87821	22	42.5	23.2	50.1
Baix Vallès	139790	856	41.8	22.3	50.2
Barcelona Dreta	408021	3909	45.1	22.8	53.5

Barcelona Esquerra	524159	2613	45.0	23.0	53.0
Barcelona Litoral Mar	316501	1386	42.4	22.1	49.8
Barcelona Nord	446848	2965	44.3	23.3	52.6
Barcelonès Nord i Baix Maresme	425260	2323	42.6	22.8	50.7
Berguedà	37942	643	45.8	23.7	49.8
Cerdanya	16508	35	42.6	22.4	49.5
Garraf	158289	1368	42.7	22.5	50.7
Garrotxa	57448	538	43.3	23.3	49.8
Gironès Nord i Pla de l'Estany	163911	1692	40.8	22.5	51.1
Gironès Sud i Selva Interior	153475	880	40.9	22.8	48.9
L'Hospitalet Sud i el Prat de Llobregat	205776	961	44.0	23.0	51.3
Lleida	364037	4493	42.7	23.0	48.9
Maresme Central	270531	2682	42.3	22.7	50.4
Montsià	51722	761	43.6	23.4	49.7
Osona	163552	1378	41.9	23.0	49.5
Pallars	18278	291	45.5	23.8	49.7
Ripollès	24263	264	46.6	23.7	50.3
Selva Marítima	80029	254	42.2	22.3	49.9
Tarragonès	260282	889	41.5	22.7	50.4
Vallès Occidental Est	400787	2663	42.2	22.8	51.0
Vallès Occidental Oest	442760	1260	40.9	22.6	50.8
Vallès Oriental Central	254665	1750	41.5	22.5	49.9
Total	*7,671,862*	*53,324*	*42.7*	*23.0*	*50.8*

Table 2. Baseline characteristics of the population with linked EMR data available, overall and stratified by CMA. Q1 is the lest deprived quintile and Q5 the most deprived quintile.

CMA	N		Age		Sex	Socio-economic status					
	Total	Excl.	Mean	SD	% w.	Q1	Q2	Q3	Q4	Q5	Miss.
Alta Ribagorça	3475	34	44.23	22.96	49.15	0.03%	16.5%	39.4%	25. 4%	0.0%	18.4%
Pallars	17912	278	45.24	23.79	49.78	16.4%	47.0%	19.8%	1.4%	0.0%	15.2%
Cerdanya	16340	29	42.51	22.31	49.67	9.0%	31.3%	10.6%	11.1%	0.0%	37.8%
Alt Urgell	19781	177	45.10	23.37	50.31	21.4%	43.4%	16.2%	4.6%	0.0%	14.3%
Aran	9176	39	41.77	21.32	49.85	1.3%	52.2%	0.0%	0.0%	0.3%	45.9%
Barcelona Dreta	381176	3169	44.71	22.70	53.58	51.0%	31.5%	11.6%	1.5%	0.4%	3.7%
Barcelona Esquerra	455847	1864	44.78	22.86	53.35	56.2%	22.4%	7.8%	5.0%	2.7%	5.5%
Baix Montseny	56149	374	41.50	22.50	49.91	10.6%	20.8%	26.9%	21.1%	3.4%	16.9%
Gironès Nord i Pla de l'Estany	158429	1169	40.45	22.45	50.80	35.7%	22.9%	10.9%	12.1%	4.4%	13.8%
Garrotxa	56436	473	43.11	23.37	49.59	17.8%	27.5%	22.6%	10.8%	4.8%	16.3%
Baix Penedès	2137	0	44.27	21.81	52.60	0.1%	8.4%	19.3%	21.1%	5.0%	45.9%
Ripollès	18087	173	45.95	23.69	50.14	9.3%	36.8%	11.4%	19.2%	5.1%	18.1%
Lleida	361297	3077	42.40	22.96	48.73	12.1%	32.9%	26.7%	11.3%	5.9%	10.9%
Osona	123442	968	42.49	23.02	49.66	13.5%	15.9%	34.3%	17.2%	5.9%	12.9%
Alt Penedès	98516	575	41.58	22.91	49.74	2.6%	17.7%	26.6%	22.8%	8.1%	22.3%
Berguedà	37438	530	45.71	23.62	49.74	6.9%	20.4%	32.9%	18.1%	8.2%	13.4%
Alt Camp i Conca de Barberà	59996	501	42.99	23.01	48.87	6.9%	12.5%	39.8%	11.5%	8.6%	20.7%
Vallès Oriental Central	253070	1381	41.45	22.42	49.76	17.4%	30.3%	14.8%	12.2%	10.3%	15.0%
Baix Llobregat Nord	135754	947	40.64	22.25	49.45	5.1%	20.6%	21.1%	29.3%	10.7%	13.0%

Garraf	157163	1081	42.55	22.53	50.72	13.4%	15.3%	23.5%	19.6%	12.9%	15.3%
Gironès Sud i Selva Interior	151498	586	40.70	22.74	48.79	8.4%	9.1%	28.7%	21.4%	13.2%	19.1%
Bages i Solsonès	197826	1683	42.69	23.21	50.14	7.3%	25.5%	26.8%	15.4%	13.6%	11.5%
L'Hospitalet Sud i el Prat de Llobregat	205333	685	43.75	23.00	51.25	2.6%	9.0%	30.7%	38.2%	14.6%	4.8%
Baix Vallès	139014	670	41.70	22.31	50.04	7.0%	20.0%	22.7%	26.8%	15.7%	8.0%
Alt Empordà	136061	661	41.81	22.84	50.08	9.1%	11.2%	31.8%	17.2%	15.7%	15.0%
Baix Llobregat Litoral i Viladecans	186903	1048	41.25	22.24	50.81	22.0%	16.2%	15.5%	21.6%	15.9%	8.9%
Altebrat	31867	279	47.04	24.00	49.12	5.9%	3.8%	28.7%	32.9%	16.3%	12.3%
Baix Empordà	35969	164	42.76	22.69	50.82	8.4%	20.7%	16.6%	16.5%	17.7%	20.0%
Barcelona Nord	393869	2367	43.93	23.19	52.36	8.6%	20.1%	19.2%	26.6%	20.0%	5.5%
Baix Camp i Priorat	198442	1391	41.63	22.84	50.41	9.0%	6.8%	15.8%	22.1%	22.4%	23.8%
Anoia	113070	686	41.99	23.08	49.68	12.5%	19.6%	15.0%	20.4%	22.7%	9.8%
Vallès Occidental Oest	196035	1024	40.23	22.37	50.31	19.0%	7.1%	11.9%	15.0%	23.5%	23.5%
Vallès Occidental Est	397724	1743	41.95	22.74	50.88	16.9%	14.2%	17.4%	20.6%	24.0%	7.0%
Tarragonès	181740	499	41.14	22.33	50.25	15.6%	14.8%	8.9%	25.5%	24.1%	11.3%
Maresme Central	267684	2147	42.14	22.69	50.34	23.7%	17.9%	11.7%	11.2%	25.0%	10.5%
Baix Llobregat Litoral i Sant Boi	125909	672	42.05	22.59	50.83	7.5%	11.1%	15.2%	34.4%	26.3%	5.5%
Baix Ebre	91969	460	44.25	23.58	49.53	0.0%	11.8%	12.3%	36.8%	26.5%	12.7%
Baix Llobregat Centre i	433839	2146	42.12	22.79	51.39	16.0%	13.0%	17.5%	19.5%	26.8%	7.2%

Fontsanta -L'H N											
Alt Maresme	70886	773	42.76	22.84	49.84	5.7%	6.6%	18.0%	28.3%	27.1%	14.6%
Barcelona Litoral Mar	318969	822	42.14	21.90	49.65	13.1%	16.2%	17.2%	13.3%	34.2%	5.9%
Montsià	50230	462	43.77	23.30	49.81	0.0%	4.7%	27.7%	19.8%	34.7%	13.1%
Barcelonès Nord i Baix Maresme	428053	1598	42.34	22.76	50.53	12.3%	7.1%	10.0%	20.8%	43.1%	6.7%
Selva Marítima	39455	200	42.89	22.51	50.07	0.0%	0.0%	14.0%	14.9%	61.2%	9.8%
SE	13660	36	42.56	21.67	52.73	4.9%	13.0%	4.8%	5.6%	4.5%	67.2%
Total	*6827626*	*39641*	*42.52*	*22.78*	*50.73*	*17.8%*	*17.8%*	*17.9%*	*17.9%*	*17.9%*	*10.6%*

Table 3. Population size, number of cases confirmed by PCR (PCR), recorded in EMR (EMR), with respective cumulative incidences/100,000 and ratio of EMR/PCR, overall and stratified by CMA. Data are based on figures recorded between July 1 and August 31st 2020.

CMA	Pop.	PCR			EMR			EMR/PCR	
		N	Inc.	Inc. 95% CI	N	Inc.	Inc. 95% CI	value	95% CI
CATALUNYA (overall)	7608261	49666	652.8	647.1 – 658.7	134071	1762.2	1756.5 – 1768.1	2.70	2.67 – 2.72
Alt Urgell	19854	143	720.3	630.4 – 2995.9	199	1002.3	912.5 – 1211.6	1.39	1.18 – 1.61
Cerdanya	16324	51	312.4	253.2 – 3080.1	169	1035.3	976.1 – 1245.5	3.31	2.66 – 3.97
Pallars	17970	50	278.2	231.9 – 2792.4	220	1224.3	1177.9 – 1416.3	4.40	3.65 – 5.15
Aran	9307	18	193.4	140.6 – 5047.8	77	827.3	774.5 – 1161.8	4.28	3.08 – 5.48
Lleida	359144	7096	1975.8	1931.9 – 2101.6	13868	3861.4	3817.5 – 3911.6	1.95	1.91 – 2.00
Alt Camp i Conca de Barberà	60621	179	295.3	261.3 – 1040.6	731	1205.9	1171.9 – 1278.1	4.08	3.60 – 4.57
Baix Camp i Priorat	199260	1027	515.4	487.1 – 742.1	3423	1717.9	1689.6 – 1757.0	3.33	3.14 – 3.52
Baix Penedès	87476	249	284.6	256.5 – 801.1	93	106.3	78.1 – 161.5	0.37	0.27 – 0.48
Tarragonès	258721	892	344.8	324.9 – 519.4	2357	911.0	891.1 – 939.8	2.64	2.48 – 2.81
Anoia	112510	278	247.1	224.5 – 648.7	1405	1248.8	1226.2 – 1293.5	5.05	4.58 – 5.53
Osona	161989	647	399.4	372.7 – 678.3	1573	971.1	944.4 – 1011.7	2.43	2.26 – 2.61
Baix Llobregat Centre i Fontsanta - L'H N	431991	5889	1363.2	1330.0 – 1467.8	11041	2555.8	2522.7 – 2594.1	1.87	1.82 – 1.93
L'Hospitalet Sud i el Prat de Llobregat	204612	1777	868.5	831.5 – 1089.3	4543	2220.3	2183.3 – 2268.3	2.56	2.44 – 2.67

Barcelonès Nord i Baix Maresme	422662	3543	838.3	812.3 – 945.2	9216	2180.5	2154.5 - 2211.7	2.60	2.52 - 2.69
Maresme Central	267757	1108	413.8	392.1 - 582.5	4119	1538.3	1516.6 - 1568.6	3.72	3.52 - 3.92
Vallès Occidental Est	398037	1982	497.9	477.8 - 611.5	7610	1911.9	1891.7 - 1937.7	3.84	3.68 - 4.00
Alta Ribagorça	3439	12	348.9	221.4 - 13486.4	64	1861.0	1733.5 - 2744.7	5.33	3.35 - 7.32
Barcelona Esquerra	520609	3852	739.9	717.9 - 826.7	10109	1941.8	1919.8 - 1968.1	2.62	2.54 - 2.71
Barcelona Nord	443154	3347	755.3	731.3 – 857.2	8098	1827.4	1803.4 – 1856.4	2.42	2.34 – 2.50
Altebrat	31964	130	406.7	356.1 – 1820.2	431	1348.4	1297.8 – 1476.5	3.32	2.88 – 3.75
Baix Ebre	92351	366	396.3	363.3 – 885.5	1582	1713.0	1680.0 – 1772.2	4.32	3.95 – 4.69
Montsià	50931	210	412.3	369.2 – 1299.4	720	1413.7	1370.5 – 1504.2	3.43	3.05 – 3.80
Alt Empordà	137786	849	616.2	579.7 – 944.1	2151	1561.1	1524.6 – 1614.1	2.53	2.37 – 2.69
Baix Empordà	127536	360	282.3	258.4 – 636.5	3172	2487.1	2463.2 – 2529.6	8.81	8.06 – 9.56
Garrotxa	56859	174	306.0	271.7 – 1100.6	568	999.0	964.6 – 1076.1	3.26	2.88 – 3.65
Ripollès	23871	81	339.3	288.1 – 2232.0	187	783.4	732.1 – 938.6	2.31	1.93 – 2.69
Alt Maresme	116991	329	281.2	256.6 – 667.4	961	821.4	796.8 – 866.4	2.92	2.65 – 3.19
Selva Marítima	79602	206	258.8	231.9 – 826.4	475	596.7	569.9 – 654.5	2.31	2.05 – 2.57
Gironès Nord i Pla de l'Estany	161987	533	329.0	305.3 – 607.9	2070	1277.9	1254.1 – 1316.0	3.88	3.59 – 4.17
Gironès Sud i Selva Interior	152451	546	358.1	332.5 – 654.5	1827	1198.4	1172.7 – 1239.1	3.35	3.10 – 3.60
Bages i Solsonès	209972	572	272.4	253.6 – 487.6	1957	932.0	913.2 – 962.4	3.42	3.18 – 3.67

Berguedà	37157	72	193.8	163.2 – 1409.7	468	1259.5	1229.0 – 1357.3	6.50	5.46 – 7.54
Alt Penedès	97275	370	380.4	348.9 – 844.8	1531	1573.9	1542.4 – 1630.3	4.14	3.79 – 4.49
Garraf	156908	473	301.5	278.7 – 589.4	1984	1264.4	1241.7 – 1302.3	4.19	3.87 – 4.52
Baix Llobregat Litoral i Viladecans	187482	1058	564.3	534.0 – 805.3	3446	1838.0	1807.7 – 1880.4	3.26	3.07 – 3.44
Baix Llobregat Litoral i Sant Boi	126024	620	492.0	458.8 – 850.5	1921	1524.3	1491.1 – 1576.1	3.10	2.88 – 3.32
Baix Llobregat Nord	159148	620	389.6	363.2 – 673.5	2177	1367.9	1341.6 – 1408.8	3.51	3.26 – 3.76
Vallès Occidental Oest	440266	2131	484.0	465.0 – 586.6	4458	1012.6	993.6 – 1036.6	2.09	2.00 – 2.18
Baix Montseny	56113	198	352.9	315.4 – 1158.0	1189	2118.9	2081.5 – 2200.0	6.01	5.36 – 6.65
Baix Vallès	138907	750	539.9	506.2 – 865.2	2710	1950.9	1917.2 – 2001.1	3.61	3.38 – 3.85
Vallès Oriental Central	252619	1892	749.0	717.8 – 927.8	5936	2349.8	2318.7 – 2389.5	3.14	3.00 – 3.27
Barcelona Dreta	403657	2454	607.9	585.7 – 719.9	6189	1533.2	1510.9 – 1561.1	2.52	2.42 – 2.62
Barcelona Litoral Mar	314967	2531	803.6	774.5 – 947.0	7045	2236.7	2207.7 – 2273.0	2.78	2.68 – 2.89

Table 4. Delay (in days) between case reports and PCR confirmation and EMR diagnosis, and the difference between both, overall and stratified by CMA.

CMA	PCR			EMR			EMR to PCR delay		
	mean	median	95%	mean	median	95%	mean	median	95%
TOTAL	*3.2*	*2*	*8*	*0.4*	*0*	*2*	*2.8*	*2*	*6*
Alt Urgell	4.4	3	13	1.2	0	6	3.2	3	7
Cerdanya	3.3	3	8	0.6	0	5	2.6	3	3
Pallars	3.8	3	10	0.8	0	5	3.0	3	5
Aran	4.3	5	8	2.0	0	20	2.3	5	12
Lleida	5.1	3	19	0.7	0	6	4.4	3	13
Alt Camp i Conca de Barberà	3.2	2	9	0.6	0	4	2.7	2	5
Baix Camp i Priorat	3.5	2	11	0.8	0	7	2.7	2	4
Baix Penedès	2.5	2	4	0.4	0	3	2.1	2	1
Tarragonès	3.2	3	9	0.8	0	6	2.4	3	3
Anoia	3.8	3	11	0.7	0	5	3.1	3	6
Osona	3.0	2	7	0.6	0	5	2.3	2	2
Baix Llobregat Centre i Fontsanta -L'H N	3.4	3	11	0.8	0	6	2.7	3	5
L'Hospitalet Sud i el Prat de Llobregat	3.1	2	8	0.6	0	5	2.5	2	3
Barcelonès Nord i Baix Maresme	3.2	2	10	0.8	0	5	2.5	2	5
Maresme Central	2.9	2	7	0.7	0	5	2.2	2	2
Vallès Occidental Est	3.4	3	10	0.7	0	5	2.7	3	5
Alta Ribagorça	3.0	3	4	0.2	0	2	2.8	3	2
Barcelona Esquerra	3.5	2	11	0.7	0	6	2.8	2	5
Barcelona Nord	3.7	3	13	0.9	0	7	2.9	3	6
Altebrat	4.6	3	12	1.2	0	7	3.5	3	5
Baix Ebre	3.6	3	11	0.5	0	4	3.1	3	7
Montsià	3.9	3	12	0.6	0	5	3.3	3	7

Alt Empordà	3.6	3	15	1.0	0	6	2.6	3	9
Baix Empordà	4.2	3	10	0.6	0	5	3.6	3	5
Garrotxa	3.2	3	9	0.7	0	5	2.4	3	4
Ripollès	3.3	3	8	1.3	0	7	2.0	3	1
Alt Maresme	3.5	2	10	0.8	0	7	2.7	2	3
Selva Marítima	4.0	3	10	1.6	0	9	2.4	3	1
Gironès Nord i Pla de l'Estany	3.5	3	10	1.0	0	6	2.5	3	4
Gironès Sud i Selva Interior	3.7	3	10	1.2	0	7	2.5	3	3
Bages i Solsonès	3.1	3	7	0.8	0	5	2.3	3	2
Berguedà	4.2	3	20	0.4	0	4	3.8	3	16
Alt Penedès	3.6	3	11	0.9	0	6	2.7	3	5
Garraf	3.6	3	9	0.6	0	6	3.0	3	3
Baix Llobregat Litoral i Viladecans	3.7	3	11	1.0	0	7	2.8	3	4
Baix Llobregat Litoral i Sant Boi	3.1	2	8	0.7	0	5	2.3	2	3
Baix Llobregat Nord	3.7	2	10	0.6	0	4	3.1	2	6
Vallès Occidental Oest	2.8	2	7	0.6	0	4	2.2	2	3
Baix Montseny	2.9	2	7	0.4	0	3	2.4	2	4
Baix Vallès	3.2	2	10	0.5	0	4	2.7	2	6
Vallès Oriental Central	3.3	2	10	0.5	0	4	2.8	2	6
Barcelona Dreta	3.5	3	10	0.8	0	7	2.6	3	3
Barcelona Litoral Mar	3.6	3	11	0.8	0	6	2.8	3	5

Table 5. Measures of concordance between EMR and PCR-based EPGs, overall and stratified by health region and by risk categories.

CMA	Pop.	Risk level											
		All		Very low		Low		Intermediate		High		Very high	
		N	Conc. (%)	N	Conc. (%)	N	Conc. (%)	N	Conc. (%)	N	Conc. (%)	N	Conc. (%)
CATALUNYA	7608768	62	74	2	0	9	100	5	20	46	78	0	
Alt Urgell	19854	62	100	15	100	16	100	6	100	5	100	20	100
Cerdanya	16327	62	100	40	100	7	100	4	100	1	100	10	100
Pallars	17970	62	100	15	100	26	100	9	100	9	100	3	100
Aran	9308	62	100	41	100	14	100	4	100	3	100	0	
Lleida	359194	62	100	0	0	0		0		0	0	62	100
Alt Camp i Conca de Barberà	60626	62	92	38	89	6	100	1	100	15	100	2	
Baix Camp i Priorat	199281	62	63	17	100	23	35	3	33	4	100	15	60
Baix Penedès	87476	62	100	27	100	13	100	5	100	17	100	0	
Tarragonès	258731	62	79	14	100	30	57	2	100	16	100	0	
Anoia	112519	62	100	11	100	42	100	5	100	4	100	0	
Osona	161996	62	60	16	50	16	44	12	67	14	100	4	
Baix Llobregat Centre i Fontsanta -L'H N	432011	62	94	2	0	5	100	1	100	6	100	48	96
L'Hospitalet Sud i el Prat de Llobregat	204627	62	84	8	25	3	67	1	100	14	79	36	100
Barcelonès Nord i Baix Maresme	422682	62	89	9	56	6	100	2	100	12	75	33	100
Maresme Central	267769	62	87	12	100	13	100	21	62	16	100	0	
Vallès Occidental Est	398055	62	87	15	100	9	67	3	0	33	100	2	
Alta Ribagorça	3439	62	56	48	44	2	100	2	100	1	100	9	100
Barcelona Esquerra	520642	62	87	2	0	7	100	1	100	39	92	13	77
Barcelona Nord	443192	62	90	9	56	3	100	5	100	16	88	29	100
Altebrat	31966	62	100	23	100	3	100	9	100	23	100	4	100
Baix Ebre	92354	62	94	14	100	4	100	20	80	24	100	0	
Montsià	50939	62	68	9	89	21	48	8	75	19	68	5	100
Alt Empordà	137793	62	61	6	100	5	20	14	7	21	86	16	75
Baix Empordà	127538	62	69	24	71	18	67	8	100	12	50	0	
Garrotxa	56859	62	94	3	100	31	87	22	100	6	100	0	

Ripollès	23873	62	100	25	100	12	100	10	100	10	100	5	100
Alt Maresme	117002	62	85	14	100	32	78	7	100	9	78	0	
Selva Marítima	79606	62	97	14	100	26	92	19	100	3	100	0	
Gironès Nord i Pla de l'Estany	161994	62	85	18	100	21	57	8	100	15	100	0	
Gironès Sud i Selva Interior	152464	62	76	25	84	17	53	2	100	15	100	3	0
Bages i Solsonès	209996	62	92	2	100	44	95	11	100	5	40	0	
Berguedà	37162	62	100	27	100	24	100	6	100	4	100	1	100
Alt Penedès	97280	62	94	9	100	8	100	25	84	20	100	0	
Garraf	156922	62	77	14	86	29	59	8	100	11	100	0	
Baix Llobregat Litoral i Viladecans	187497	62	94	10	100	11	100	2	100	31	90	8	88
Baix Llobregat Litoral i Sant Boi	126030	62	100	12	100	2	100	11	100	37	100	0	0
Baix Llobregat Nord	159157	62	94	17	82	4	100	10	100	31	97	0	
Vallès Occidental Oest	440279	62	76	19	58	8	25	2	50	33	100	0	
Baix Montseny	56136	62	98	16	94	9	100	16	100	21	100	0	
Baix Vallès	138912	62	97	15	100	10	100	6	100	20	100	11	82
Vallès Oriental Central	252643	62	87	16	100	10	60	3	0	8	100	25	96
Barcelona Dreta	403687	62	90	9	33	2	100	6	100	45	100	0	
Barcelona Litoral Mar	314980	62	85	6	17	8	100	2	100	10	70	36	97
Summary for all CMA		*2728*	*88*	*688*	*86*	*609*	*81*	*327*	*85*	*704*	*93*	*400*	*93*

Fig. 5 Delay in days compared to case confirmation (x axis) vs fraction of reported cases identified (y axis). Vertical lines show the area covering 95 of the estimates.

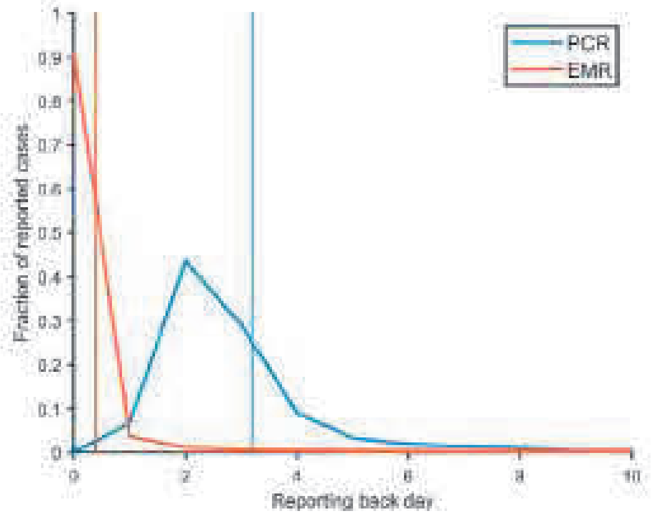

Fig. 6 Scatter plot of EMR vs PCR-based EPGs. Color depends on distance to the diagonal (dark blue zero distance to yellow if distance is bigger or equal to 100).

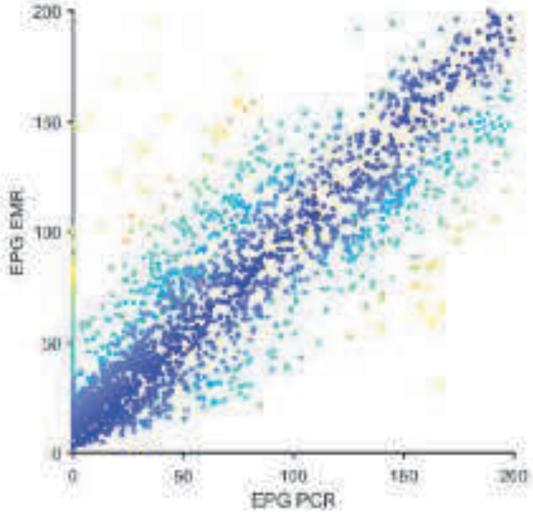

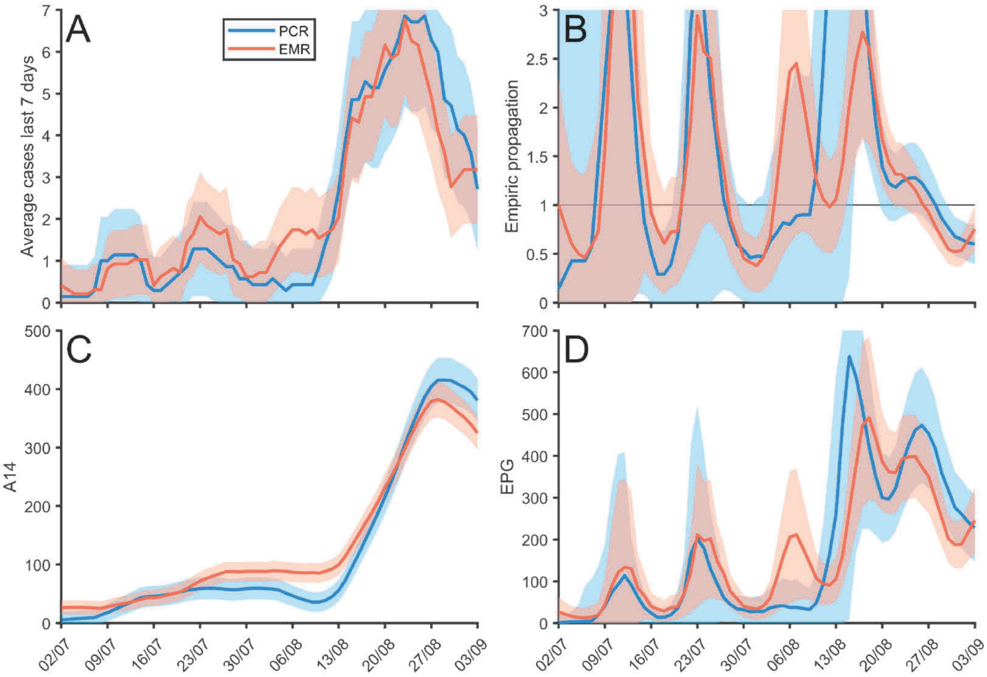

Fig. 7 Weekly average of daily new cases (A), ρ_7 empirical propagation (B), A_{14} attack rate (C) and EPG empirical propagation growth (D) over time for Alt Urgell.

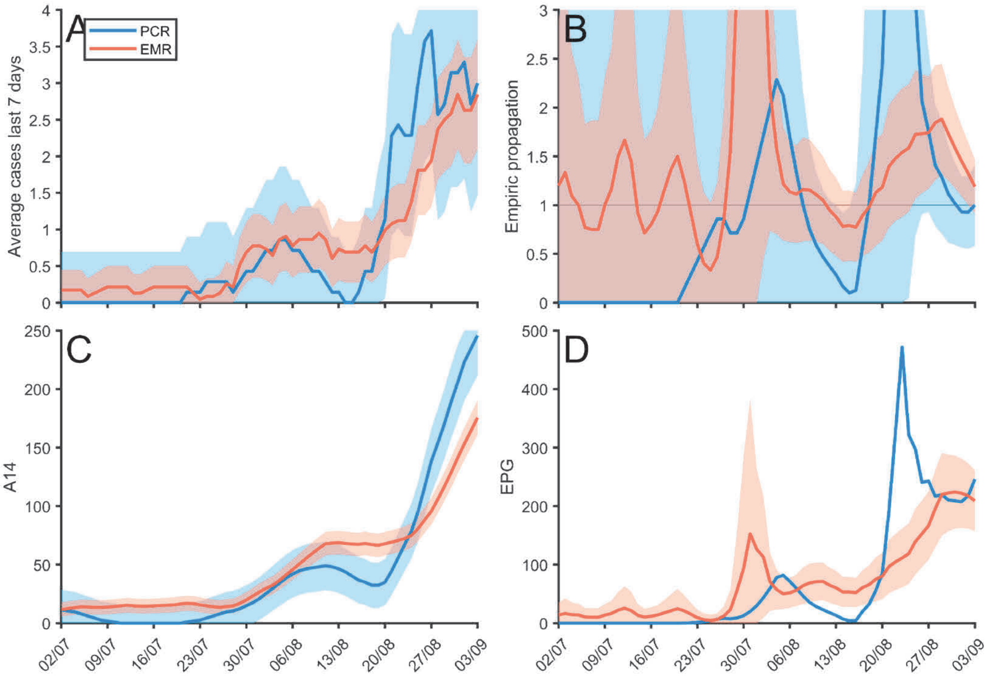

Fig. 8 Weekly average of daily new cases (A), ρ_7 empirical propagation (B), A_{14} attack rate (C) and EPG empirical propagation growth (D) over time for Cerdanya.

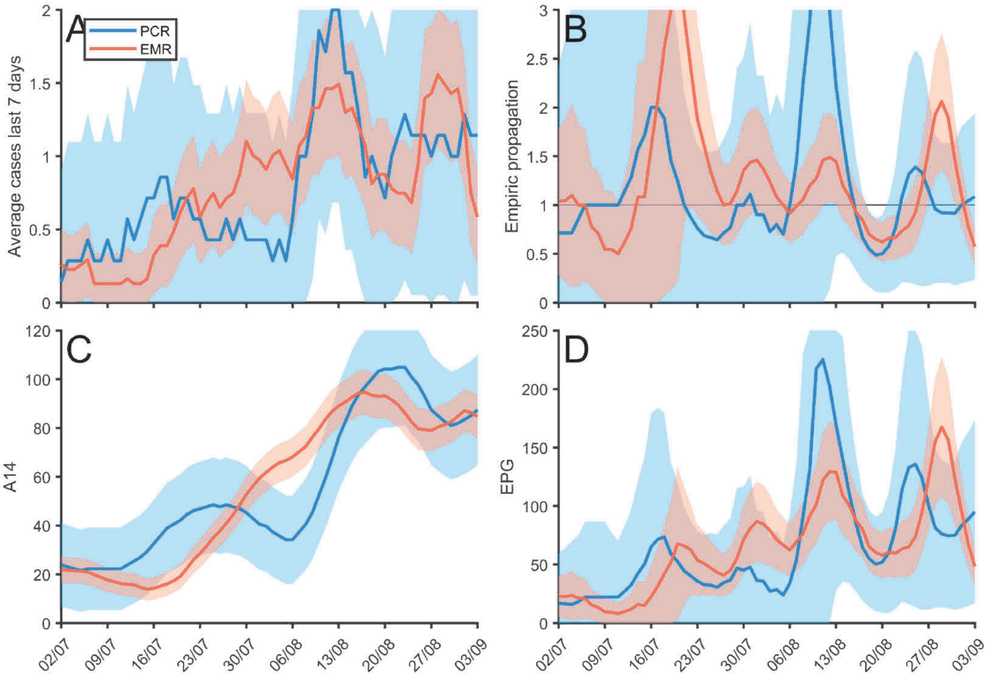

Fig. 9 Weekly average of daily new cases (A), ρ_7 empirical propagation (B), A_{14} attack rate (C) and EPG empirical propagation growth (D) over time for Pallars.

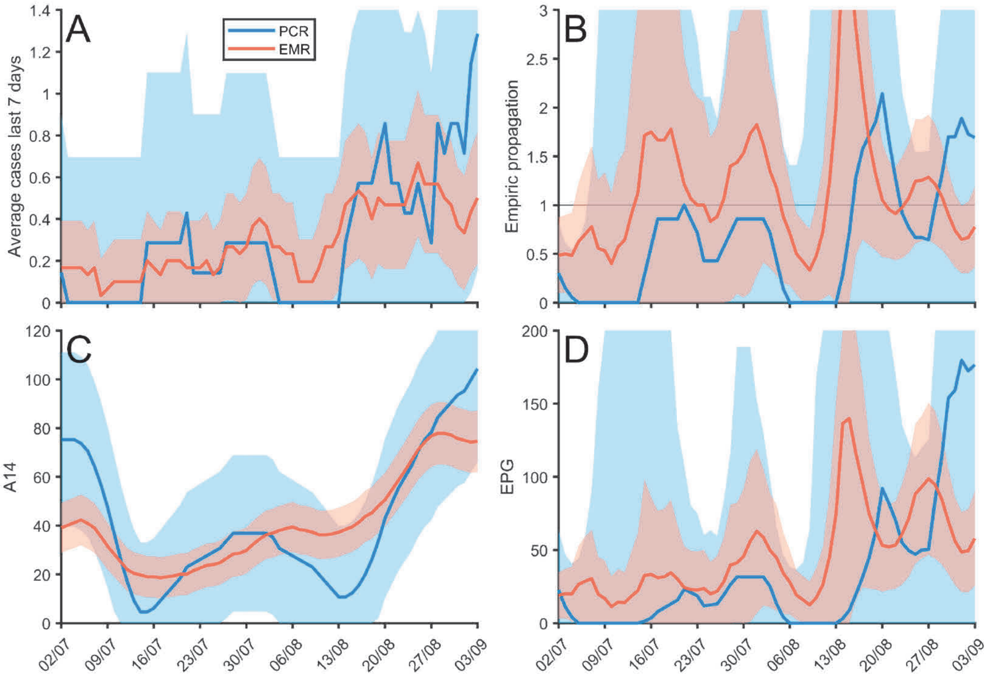

Fig. 10 Weekly average of daily new cases (A), ρ_7 empirical propagation (B), A_{14} attack rate (C) and EPG empirical propagation growth (D) over time for Aran.

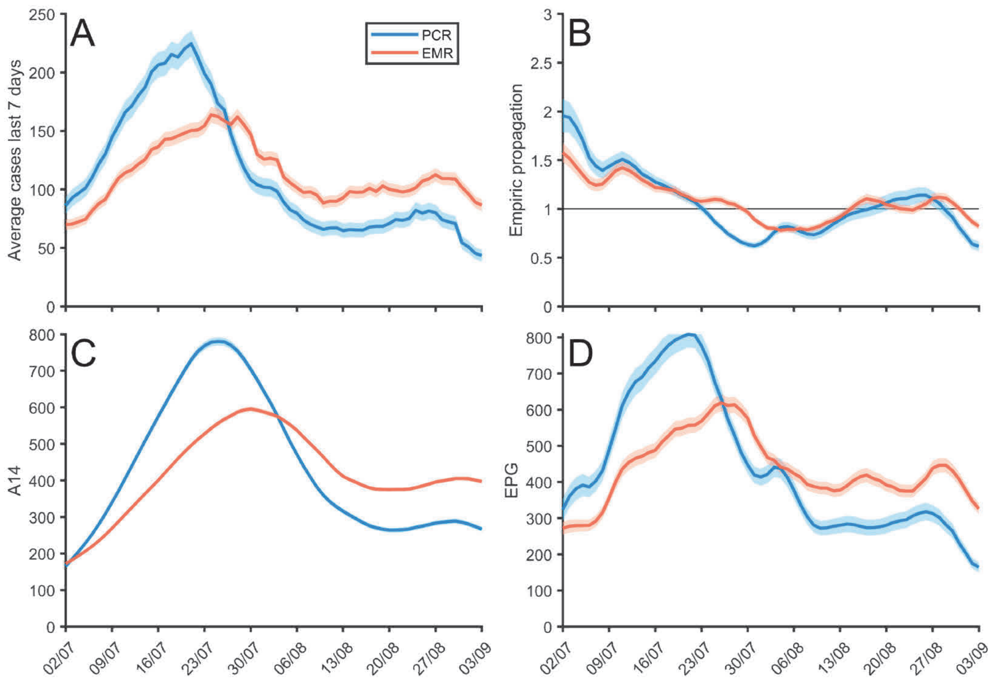

Fig. 11 Weekly average of daily new cases (A), ρ_7 empirical propagation (B), A_{14} attack rate (C) and EPG empirical propagation growth (D) over time for Lleida.

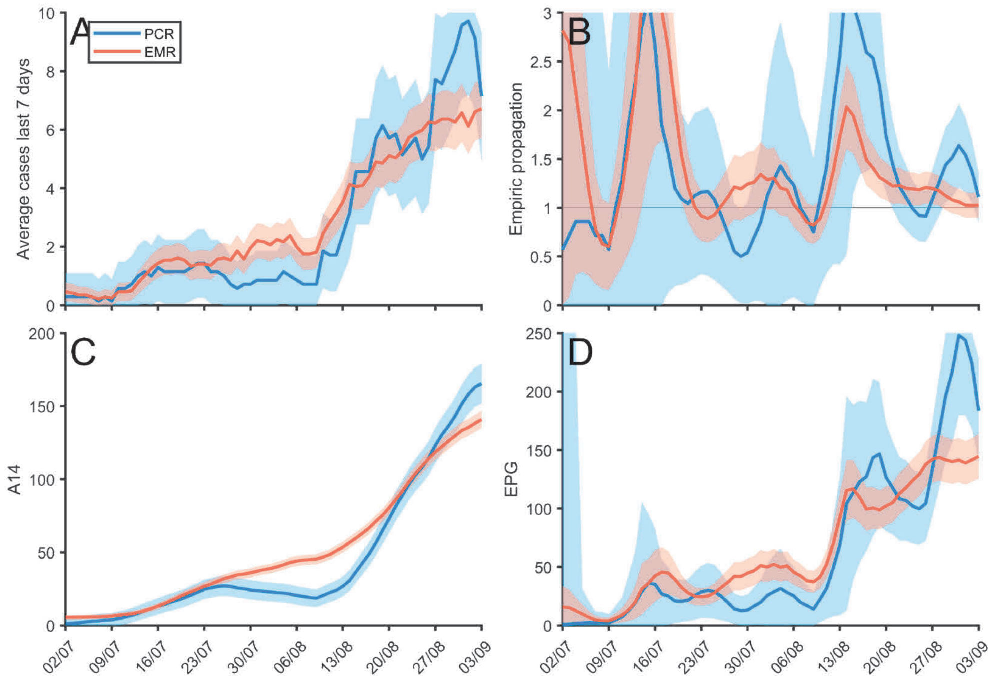

Fig. 12 Weekly average of daily new cases (A), ρ_7 empirical propagation (B), A_{14} attack rate (C) and EPG empirical propagation growth (D) over time for Alt Camp i Conca de Barberà.

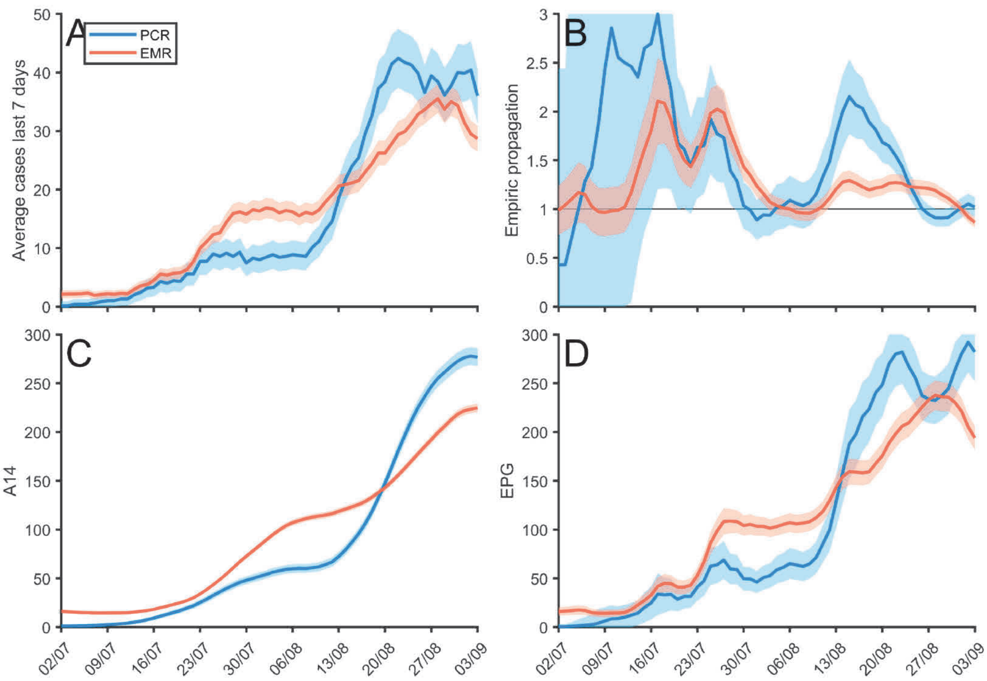

Fig. 13 Weekly average of daily new cases (A), ρ_7 empirical propagation (B), A_{14} attack rate (C) and EPG empirical propagation growth (D) over time for Baix Camp i Priorat.

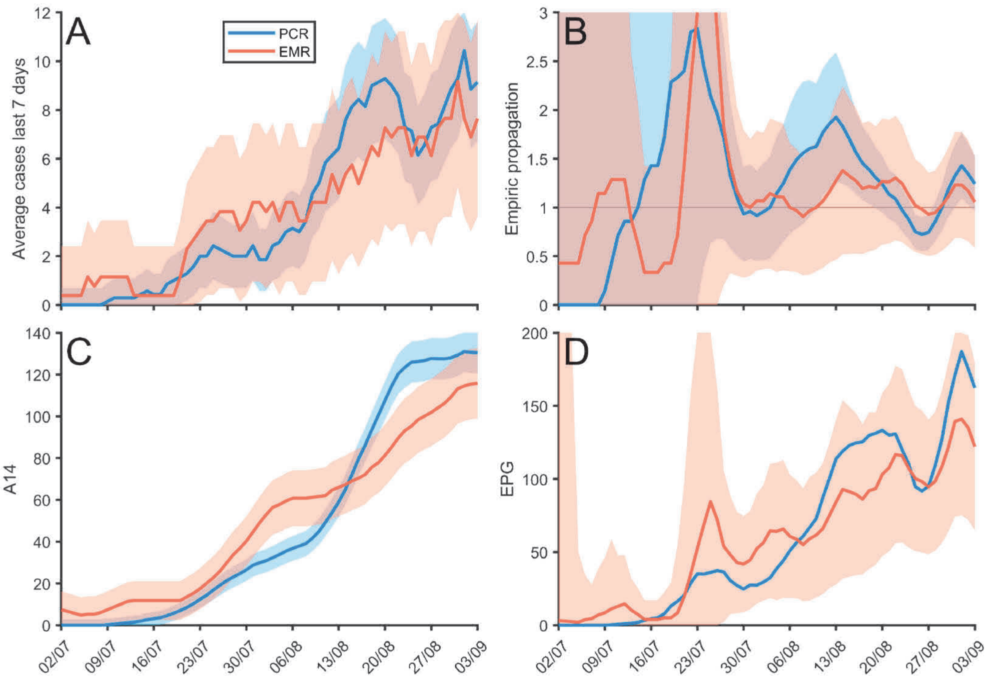

Fig. 14 Weekly average of daily new cases (A), ρ_7 empirical propagation (B), A_{14} attack rate (C) and EPG empirical propagation growth (D) over time for Baix Penedès.

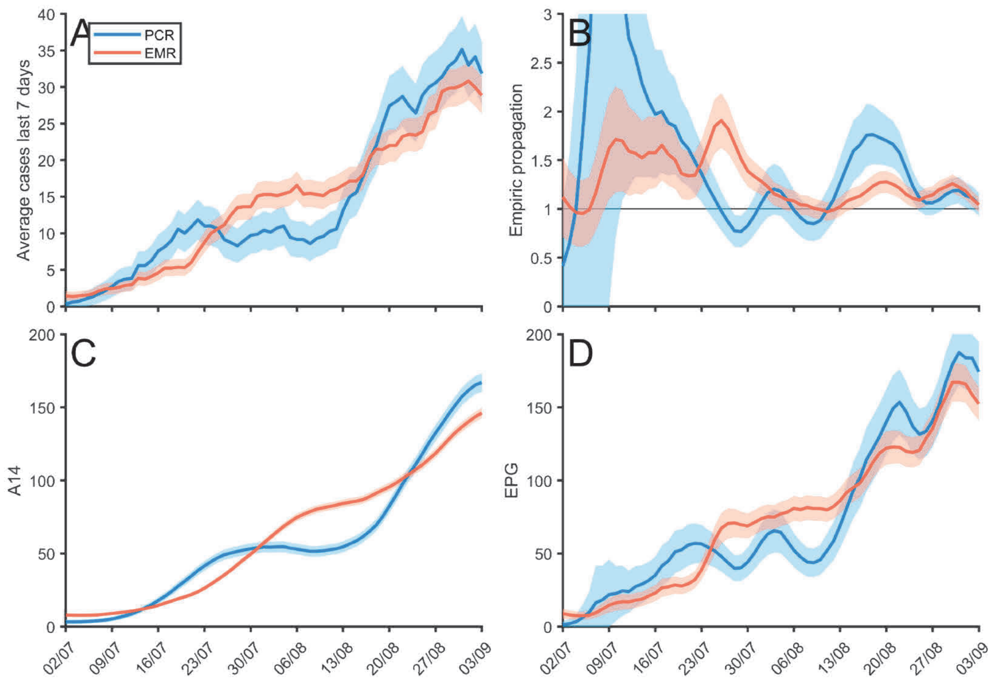

Fig. 15 Weekly average of daily new cases (A), ρ_7 empirical propagation (B), A_{14} attack rate (C) and EPG empirical propagation growth (D) over time for Tarragonès.

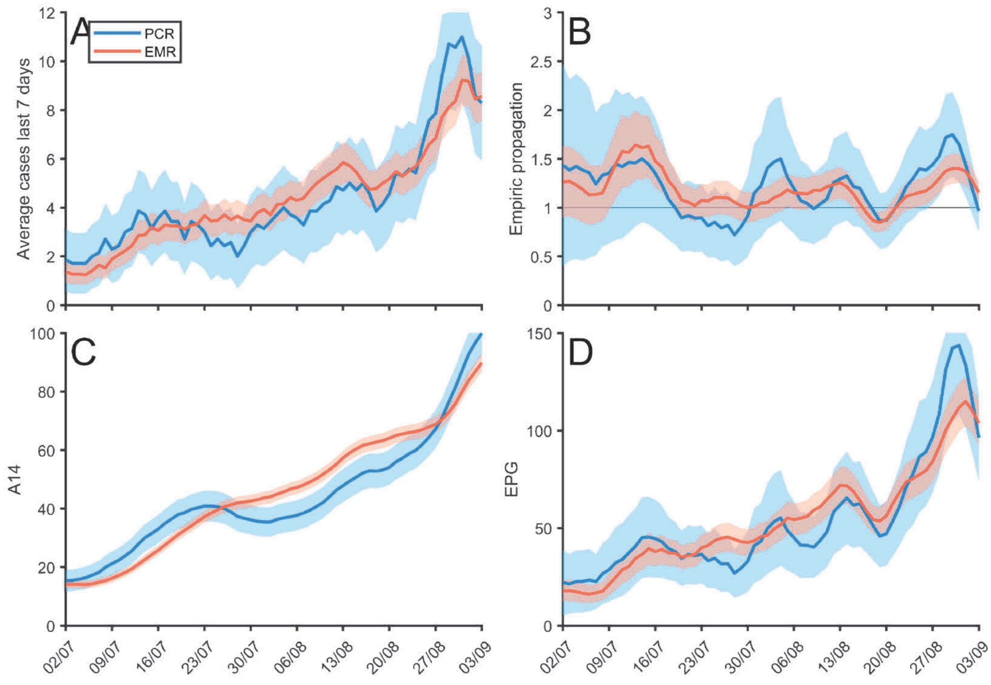

Fig. 16 Weekly average of daily new cases (A), ρ_7 empirical propagation (B), A_{14} attack rate (C) and EPG empirical propagation growth (D) over time for Anoia.

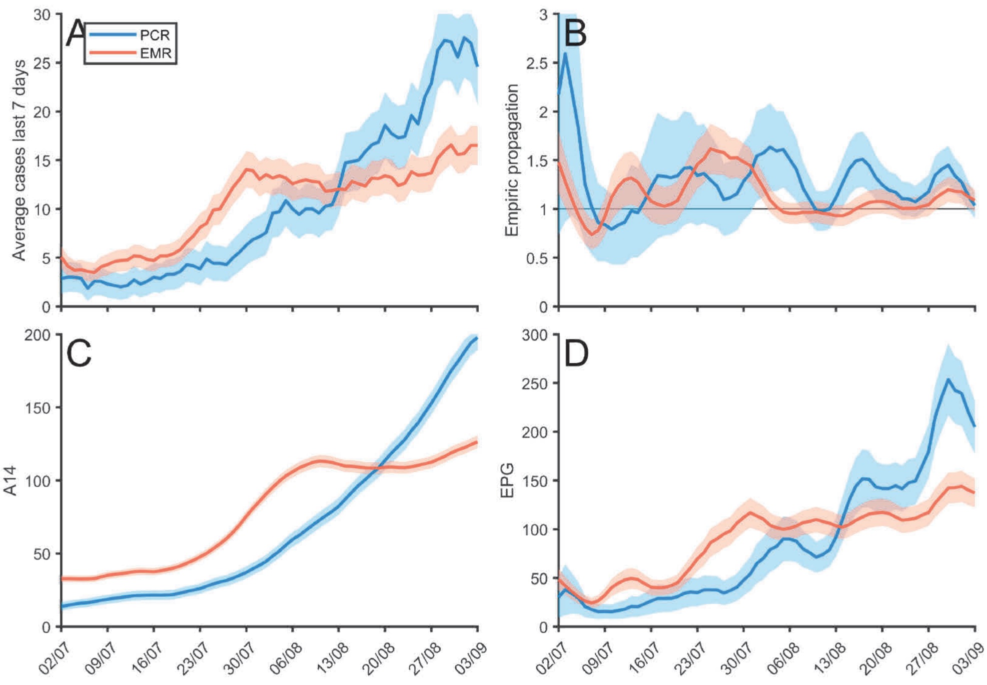

Fig. 17 Weekly average of daily new cases (A), ρ_7 empirical propagation (B), A_{14} attack rate (C) and EPG empirical propagation growth (D) over time for Osona.

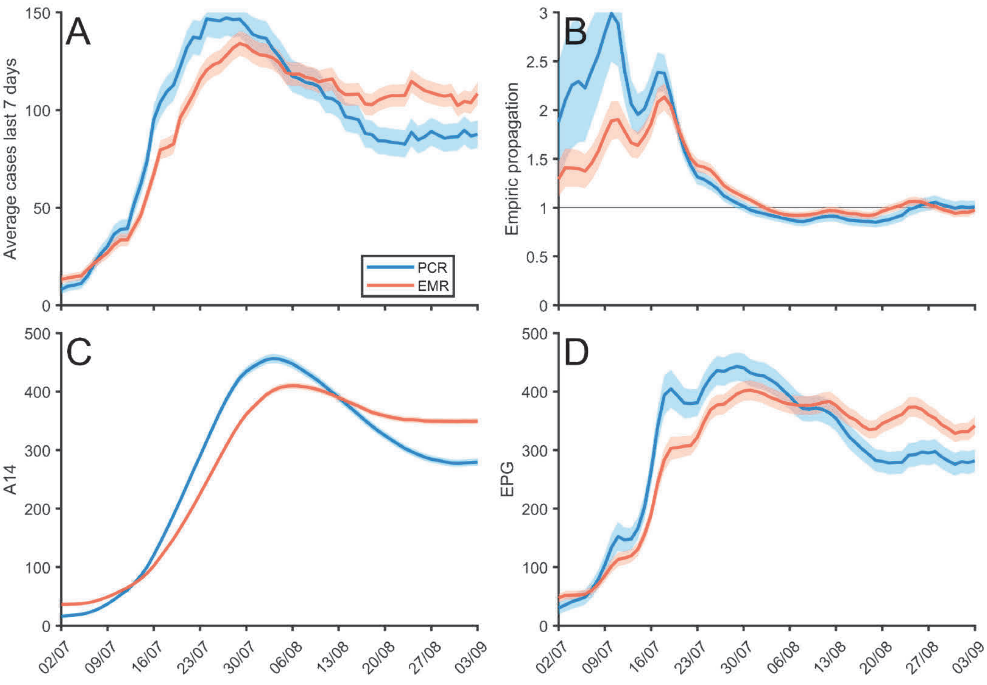

Fig. 18 Weekly average of daily new cases (A), ρ_7 empirical propagation (B), A_{14} attack rate (C) and EPG empirical propagation growth (D) over time for Baix Llobregat Centre i Fontsanta -L'H N.

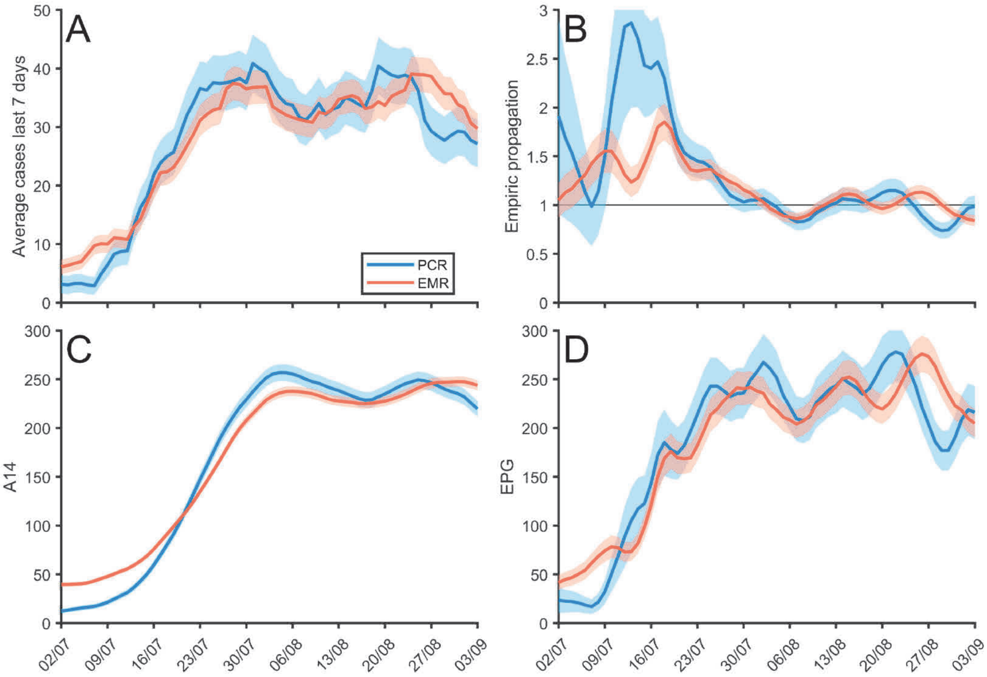

Fig. 19 Weekly average of daily new cases (A), ρ_7 empirical propagation (B), A_{14} attack rate (C) and EPG empirical propagation growth (D) over time for L'Hospitalet Sud i el Prat de Llobregat.

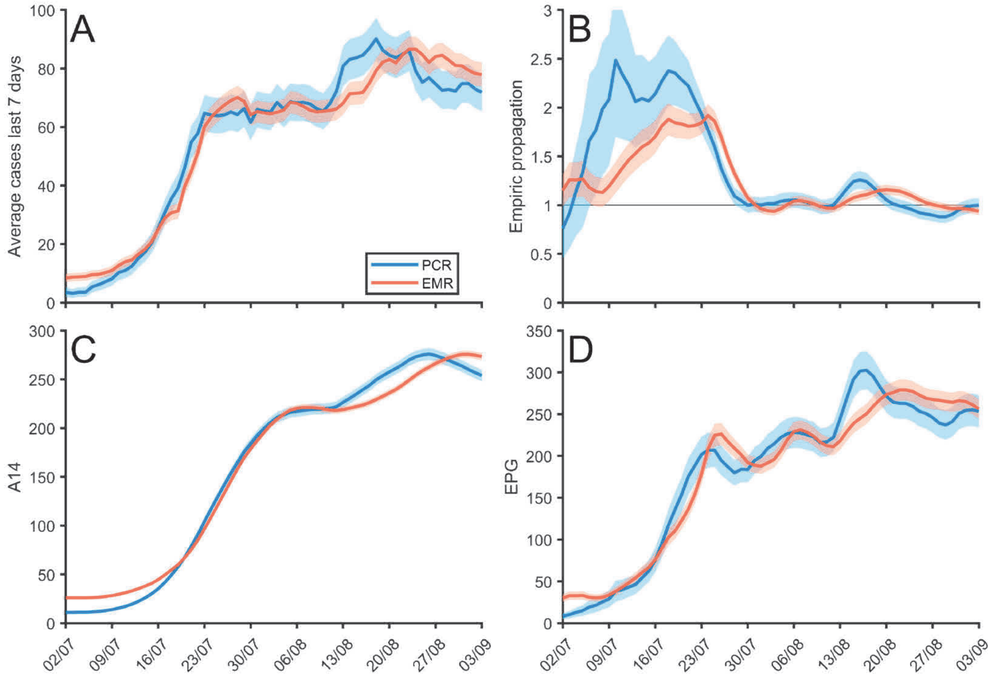

Fig. 20 Weekly average of daily new cases (A), ρ_7 empirical propagation (B), A_{14} attack rate (C) and EPG empirical propagation growth (D) over time for Barcelonès Nord i Baix Maresme.

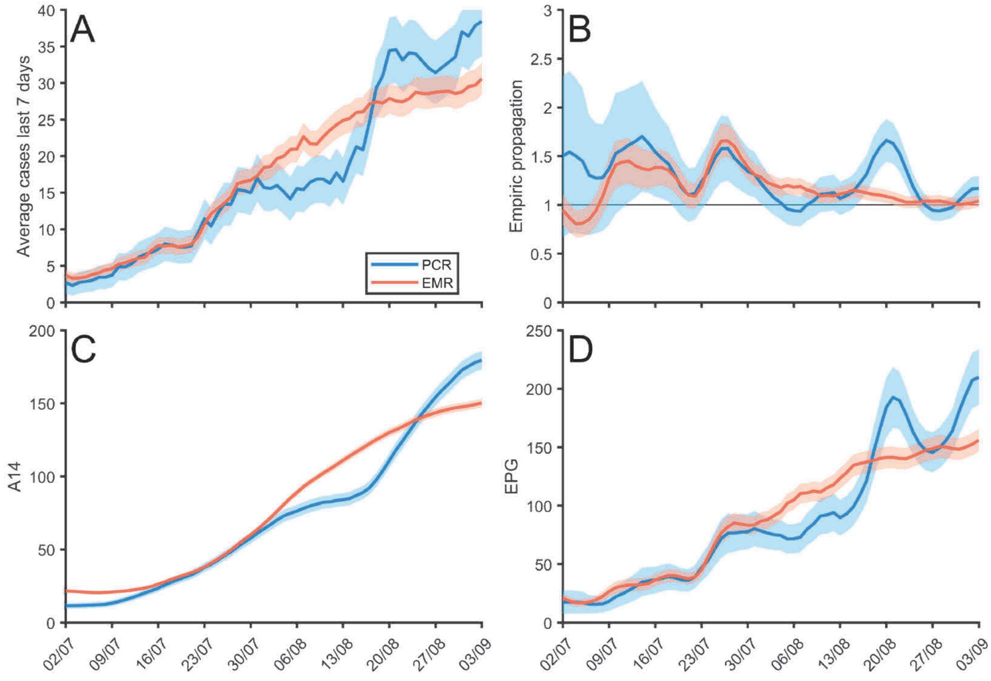

Fig. 21 Weekly average of daily new cases (A), ρ_7 empirical propagation (B), A_{14} attack rate (C) and EPG empirical propagation growth (D) over time for Maresme Central.

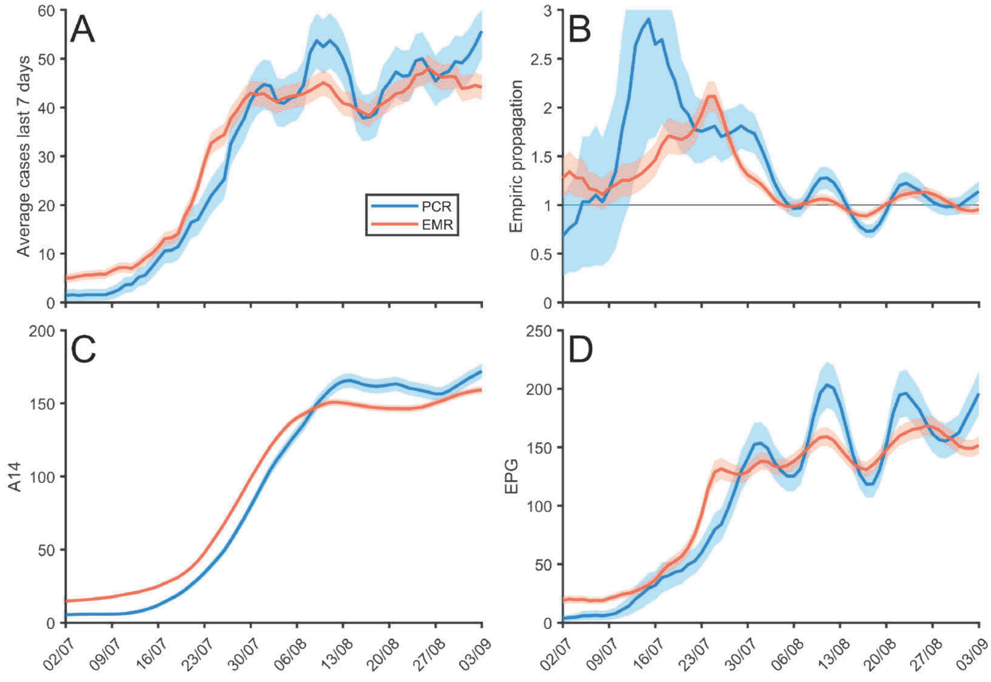

Fig. 22 Weekly average of daily new cases (A), ρ_7 empirical propagation (B), A_{14} attack rate (C) and EPG empirical propagation growth (D) over time for Vallès Occidental Est.

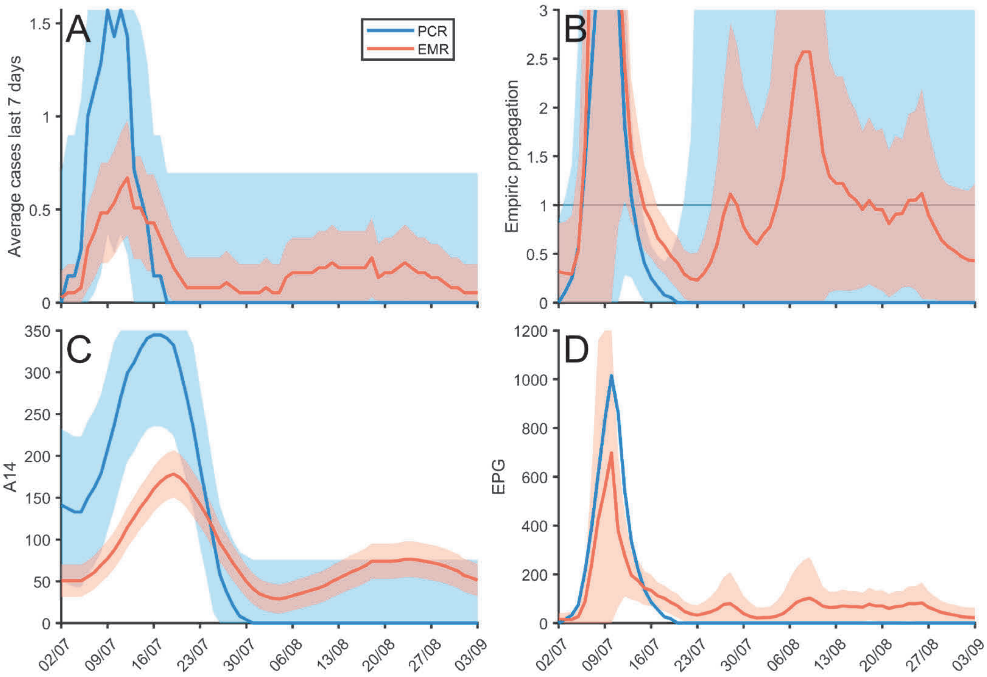

Fig. 23 Weekly average of daily new cases (A), ρ_7 empirical propagation (B), A_{14} attack rate (C) and EPG empirical propagation growth (D) over time for Alta Ribagorça.

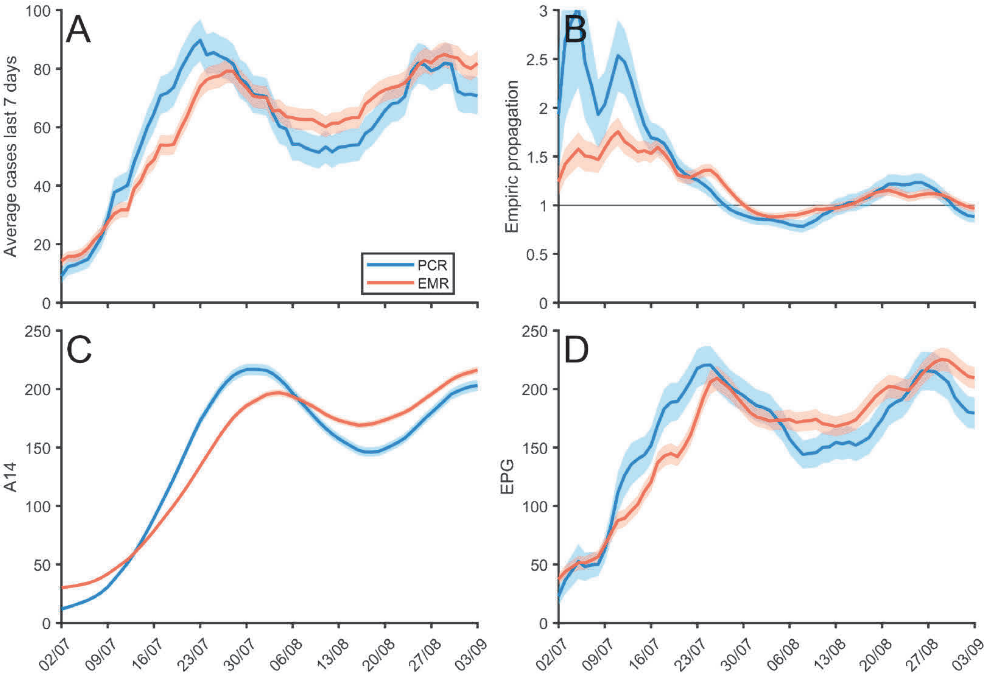

Fig. 24 Weekly average of daily new cases (A), ρ_7 empirical propagation (B), A_{14} attack rate (C) and EPG empirical propagation growth (D) over time for Barcelona Esquerra.

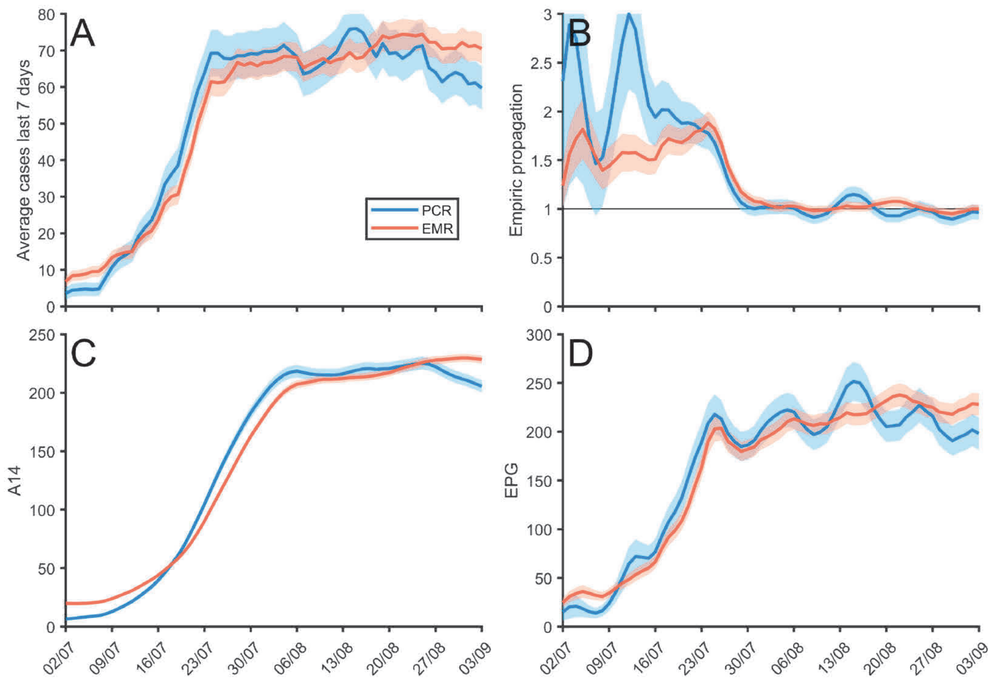

Fig. 25 Weekly average of daily new cases (A), ρ_7 empirical propagation (B), A_{14} attack rate (C) and EPG empirical propagation growth (D) over time for Barcelona Nord.

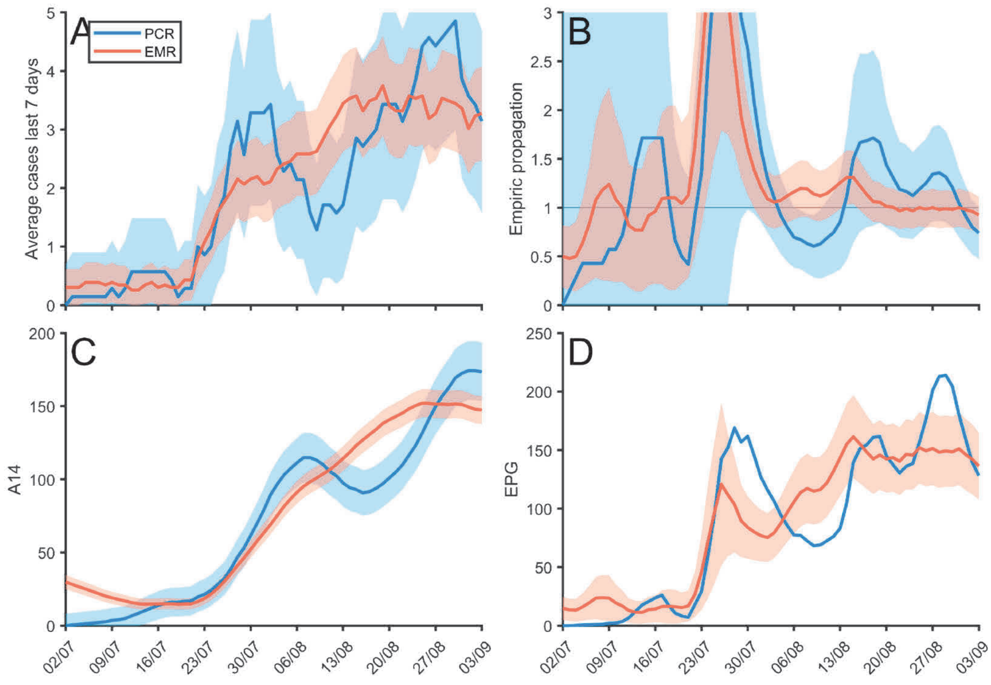

Fig. 26 Weekly average of daily new cases (A), ρ_7 empirical propagation (B), A_{14} attack rate (C) and EPG empirical propagation growth (D) over time for Altebrat.

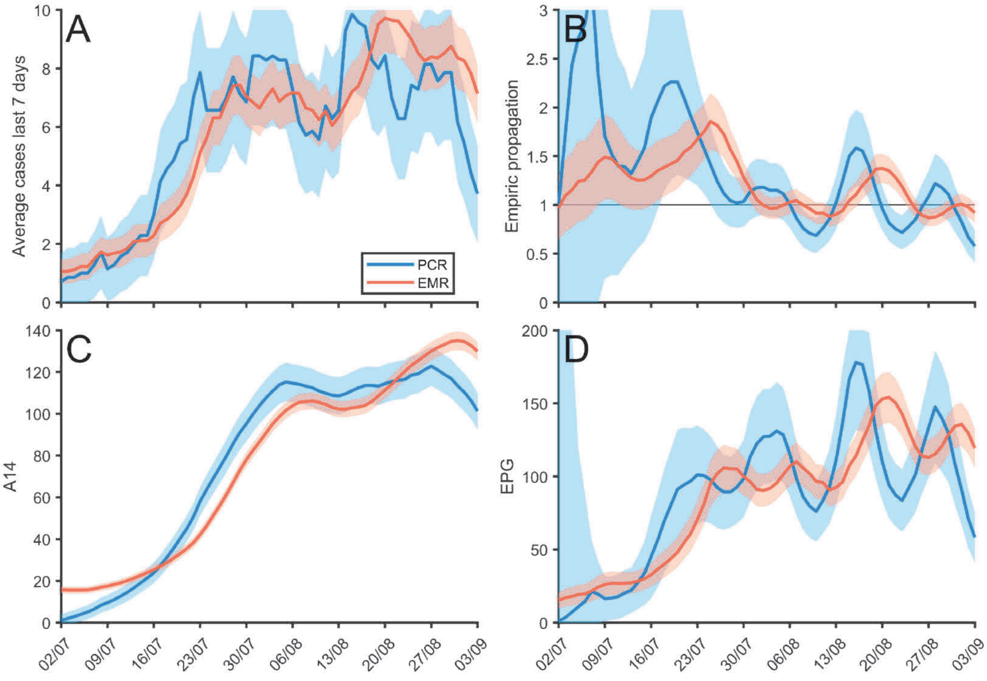

Fig. 27 Weekly average of daily new cases (A), ρ_7 empirical propagation (B), A_{14} attack rate (C) and EPG empirical propagation growth (D) over time for Baix Ebre.

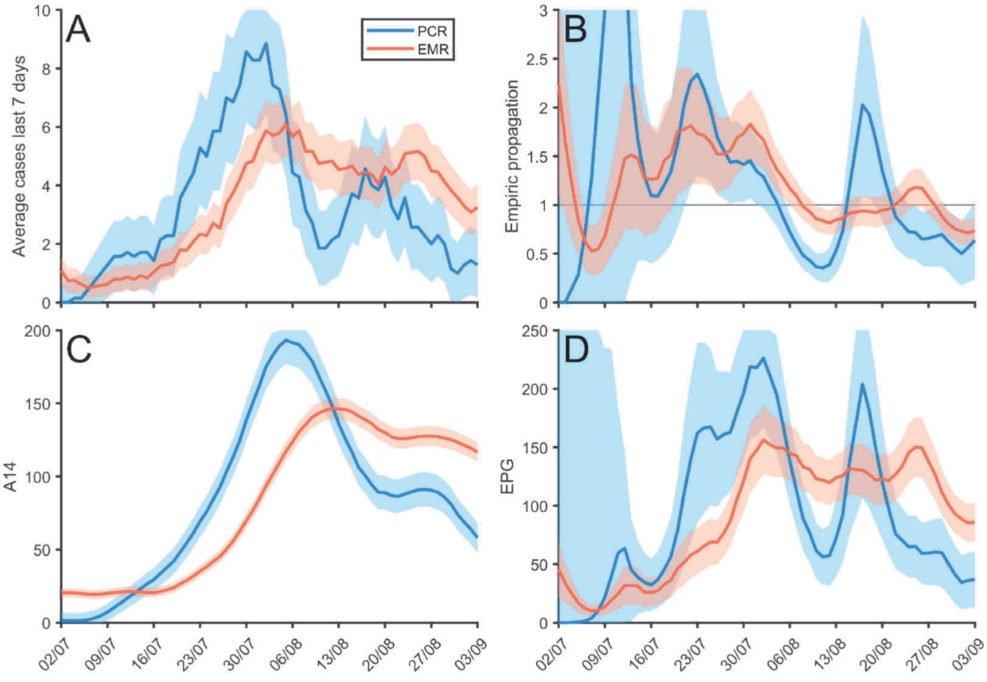

Fig. 28 Weekly average of daily new cases (A), ρ_7 empirical propagation (B), A_{14} attack rate (C) and EPG empirical propagation growth (D) over time for Montsià.

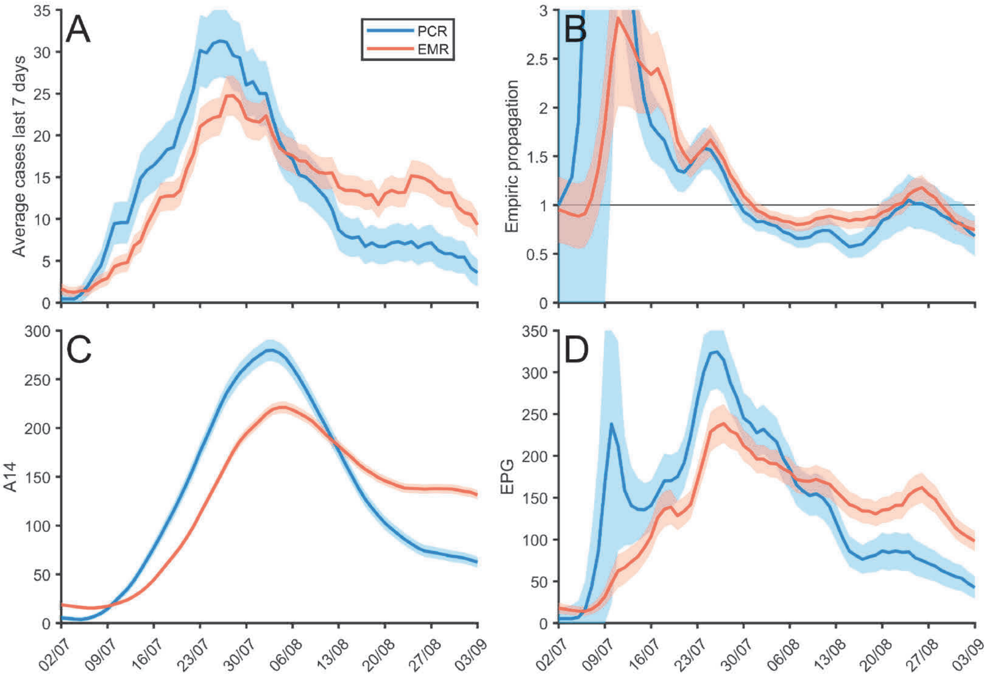

Fig. 29 Weekly average of daily new cases (A), ρ_7 empirical propagation (B), A_{14} attack rate (C) and EPG empirical propagation growth (D) over time for Alt Empordà.

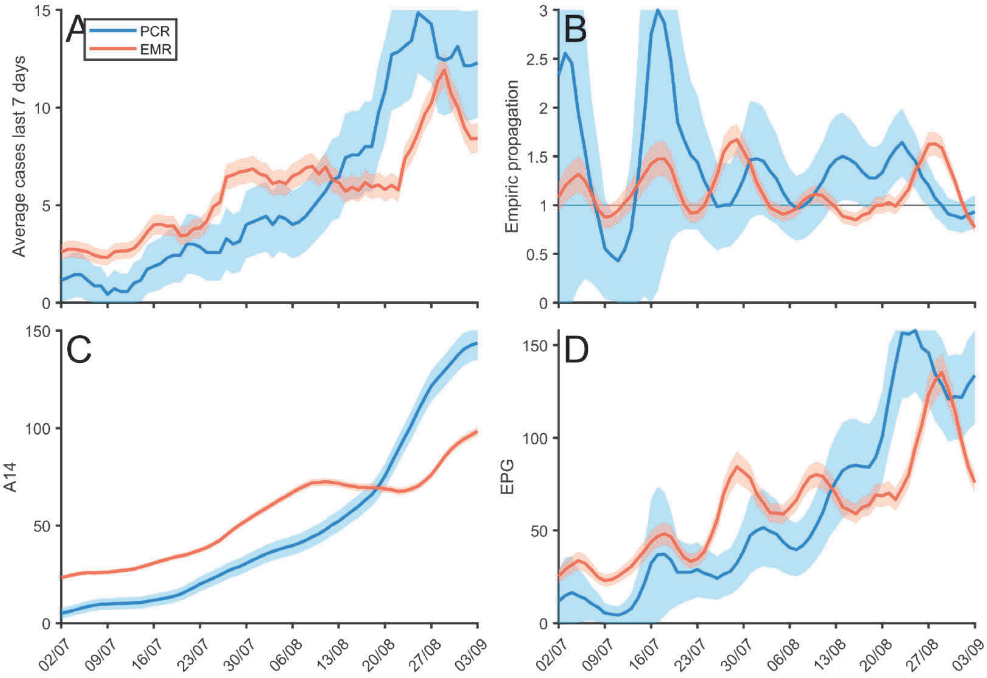

Fig. 30 Weekly average of daily new cases (A), ρ_7 empirical propagation (B), A_{14} attack rate (C) and EPG empirical propagation growth (D) over time for Baix Empordà.

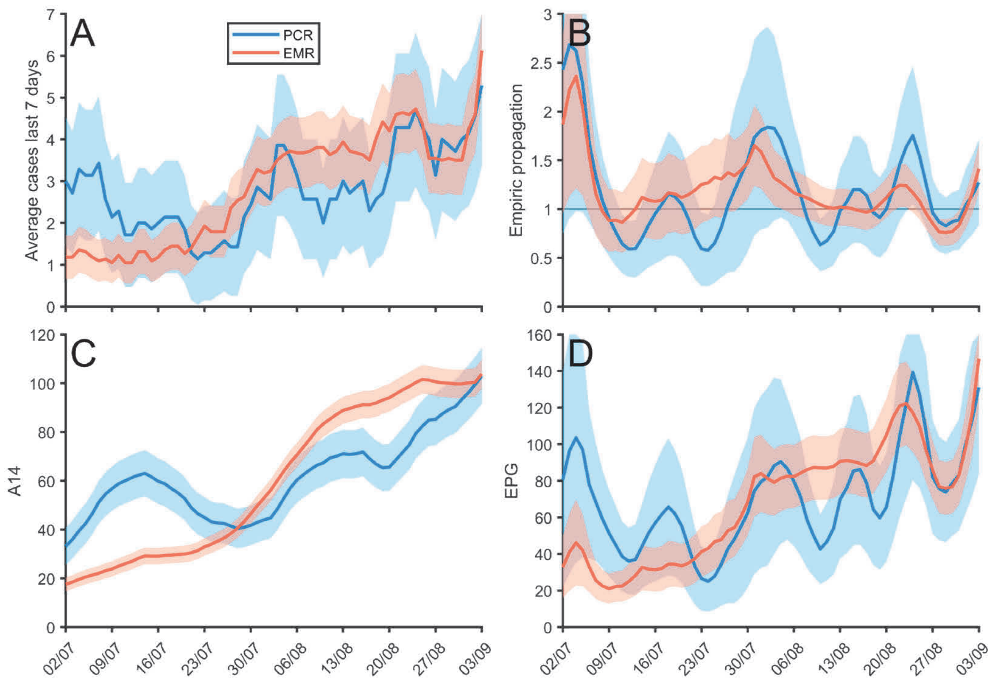

Fig. 31 Weekly average of daily new cases (A), ρ_7 empirical propagation (B), A_{14} attack rate (C) and EPG empirical propagation growth (D) over time for Garrotxa.

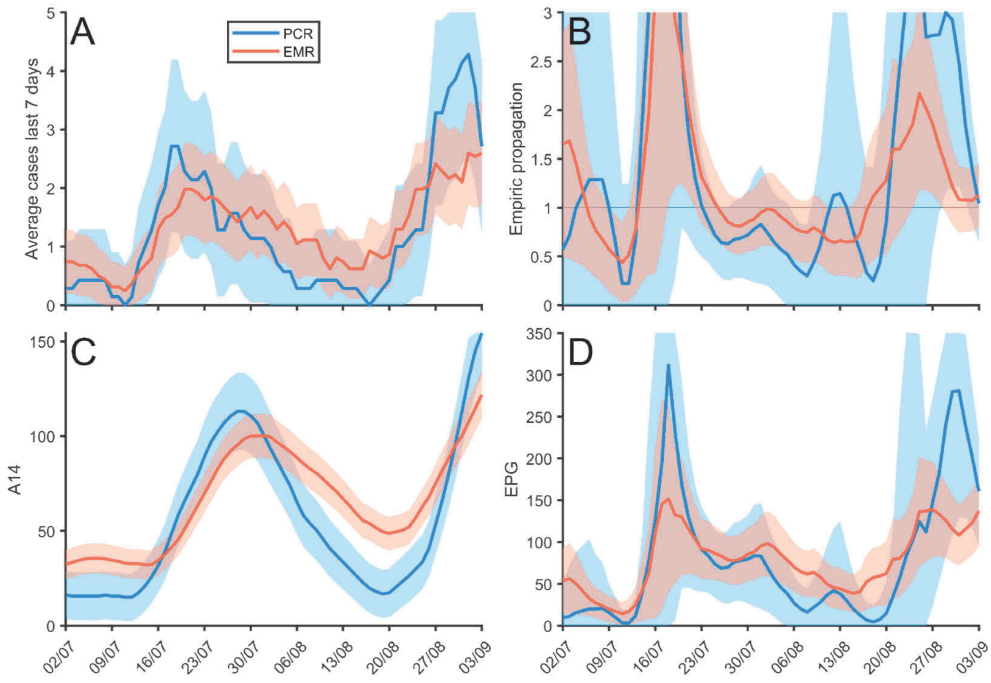

Fig. 32 Weekly average of daily new cases (A), ρ_7 empirical propagation (B), A_{14} attack rate (C) and EPG empirical propagation growth (D) over time for Ripollès.

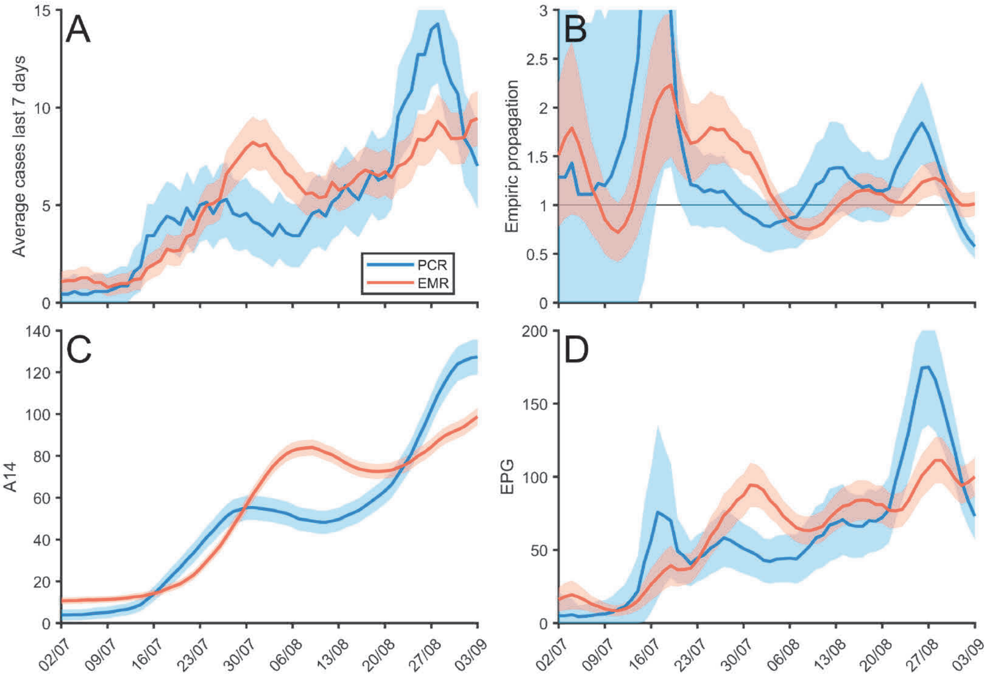

Fig. 33 Weekly average of daily new cases (A), ρ_7 empirical propagation (B), A_{14} attack rate (C) and EPG empirical propagation growth (D) over time for Alt Maresme.

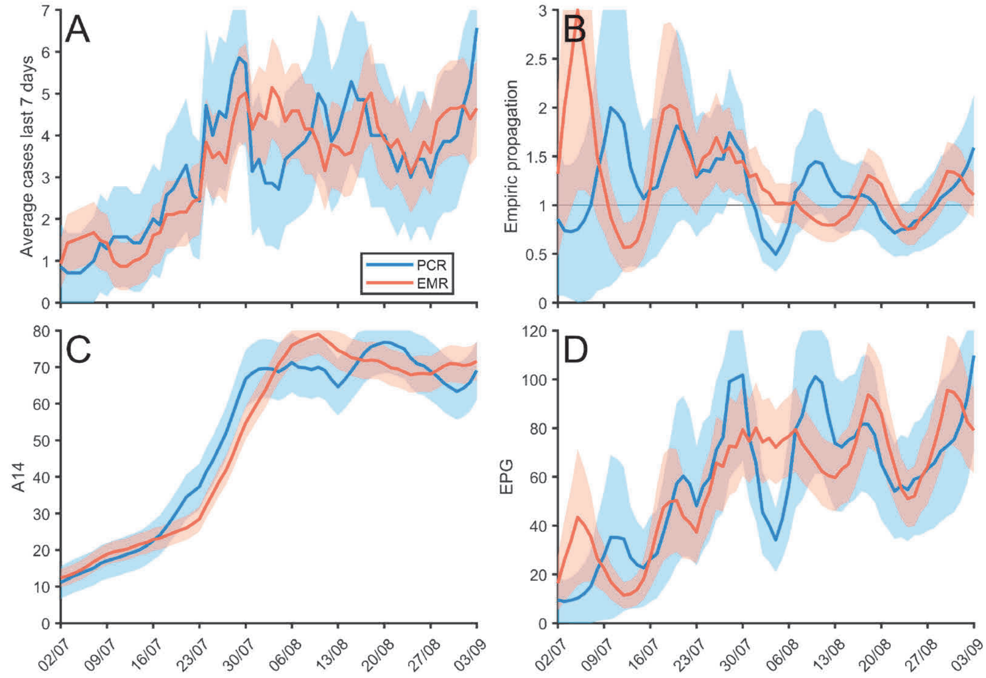

Fig. 34 Weekly average of daily new cases (A), ρ_7 empirical propagation (B), A_{14} attack rate (C) and EPG empirical propagation growth (D) over time for Selva Marítima.

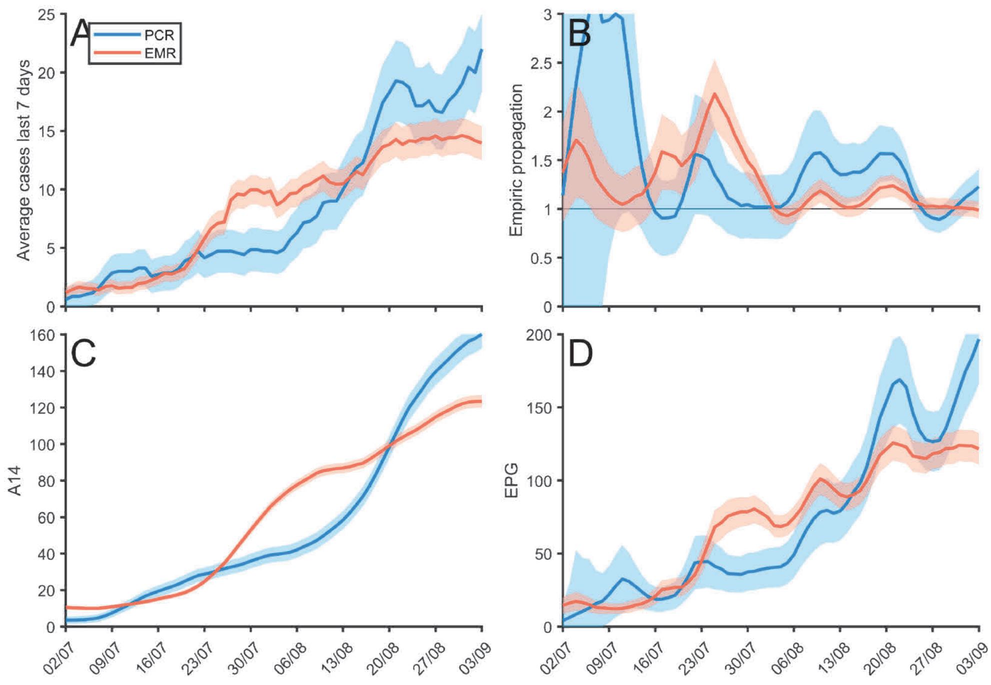

Fig. 35 Weekly average of daily new cases (A), ρ_7 empirical propagation (B), A_{14} attack rate (C) and EPG empirical propagation growth (D) over time for Gironès Nord i Pla de l'Estany.

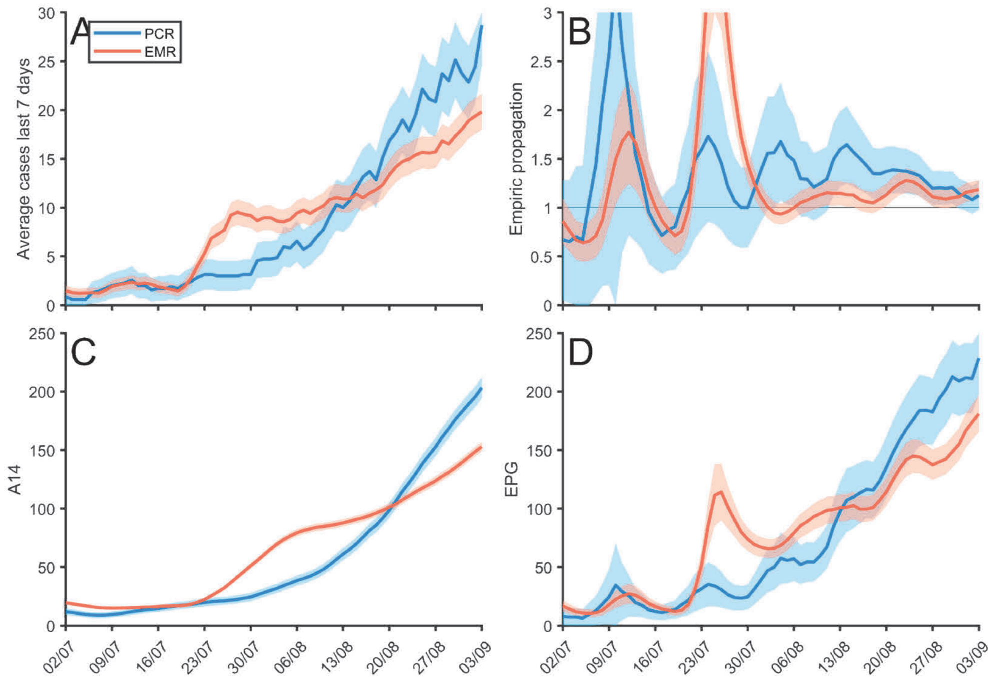

Fig. 36 Weekly average of daily new cases (A), ρ_7 empirical propagation (B), A_{14} attack rate (C) and EPG empirical propagation growth (D) over time for Gironès Sud i Selva Interior.

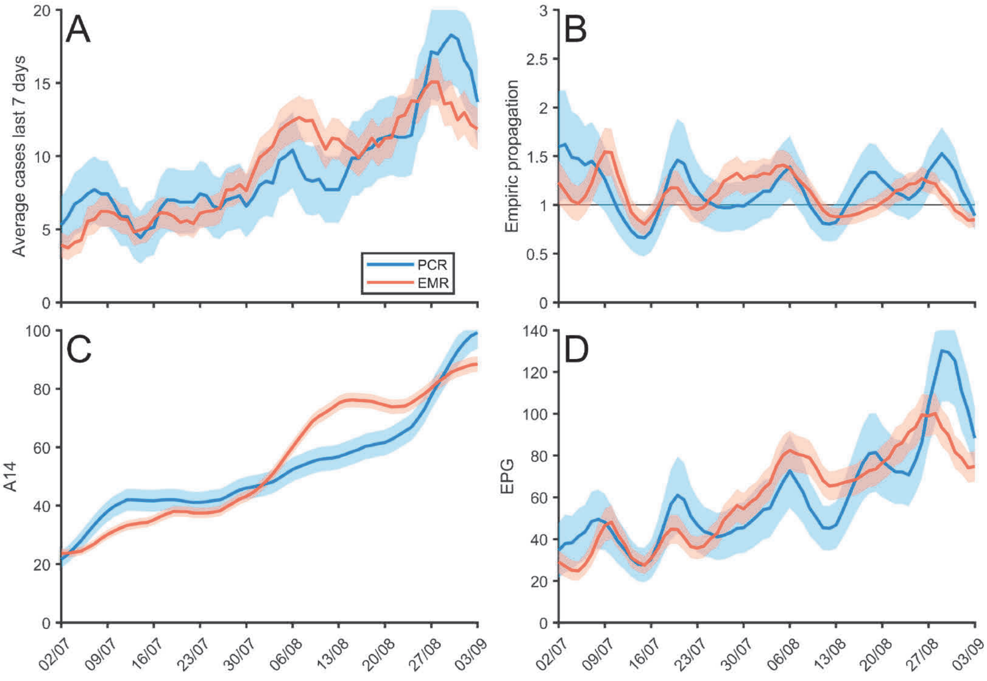

Fig. 37 Weekly average of daily new cases (A), ρ_7 empirical propagation (B), A_{14} attack rate (C) and EPG empirical propagation growth (D) over time for Bages i Solsonès.

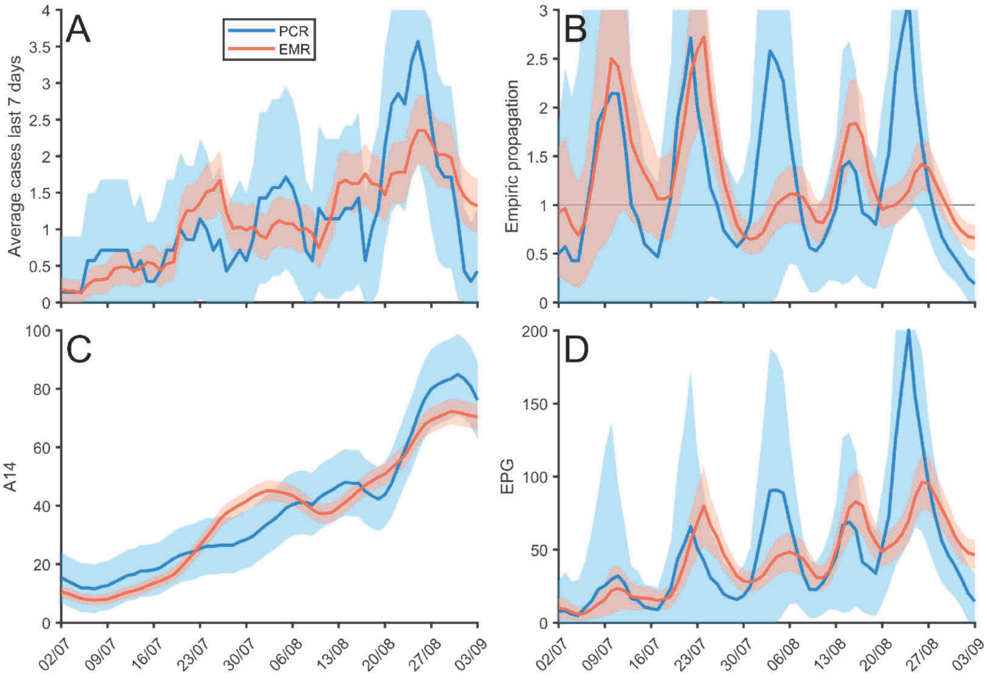

Fig. 38 Weekly average of daily new cases (A), ρ_7 empirical propagation (B), A_{14} attack rate (C) and EPG empirical propagation growth (D) over time for Berguedà.

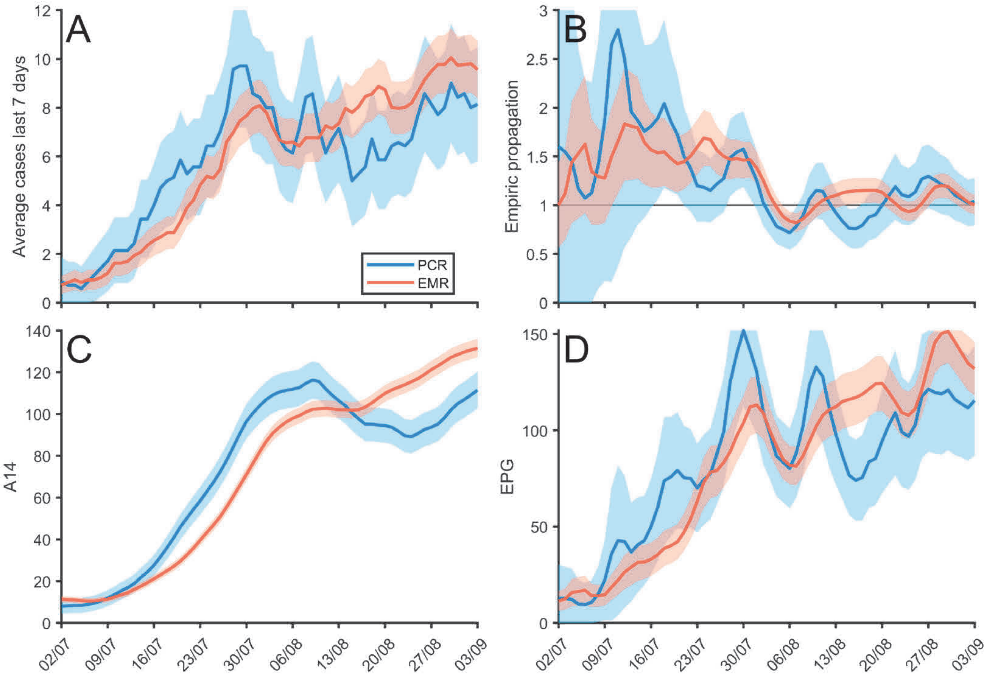

Fig. 39 Weekly average of daily new cases (A), ρ_7 empirical propagation (B), A_{14} attack rate (C) and EPG empirical propagation growth (D) over time for Alt Penedès.

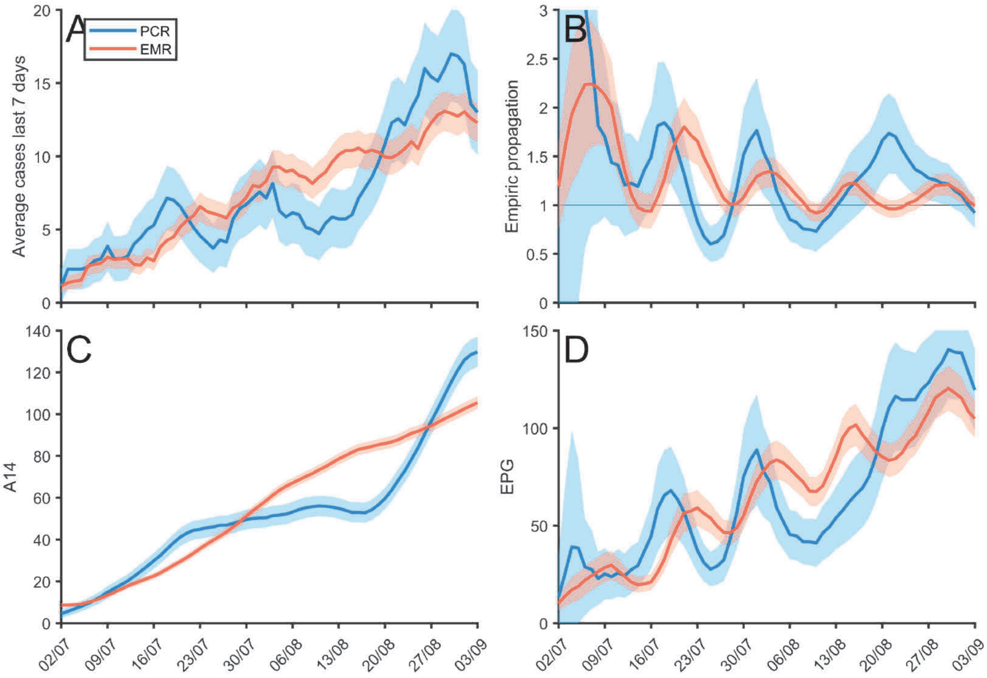

Fig. 40 Weekly average of daily new cases (A), ρ_7 empirical propagation (B), A_{14} attack rate (C) and EPG empirical propagation growth (D) over time for Garraf.

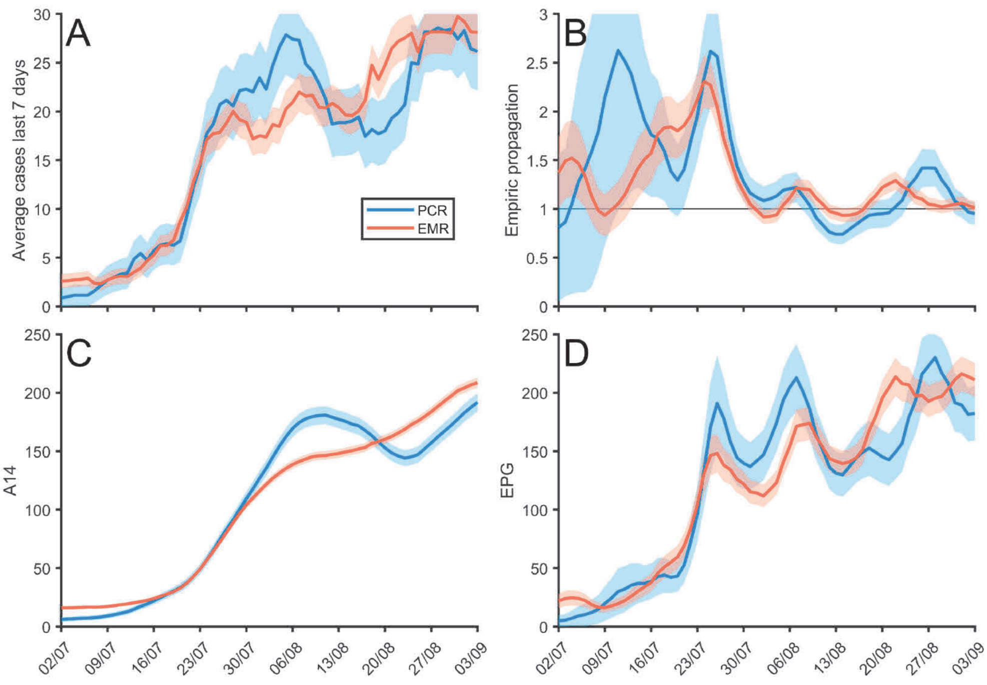

Fig. 41 Weekly average of daily new cases (A), ρ_7 empirical propagation (B), A_{14} attack rate (C) and EPG empirical propagation growth (D) over time for Baix Llobregat Litoral i Viladecans.

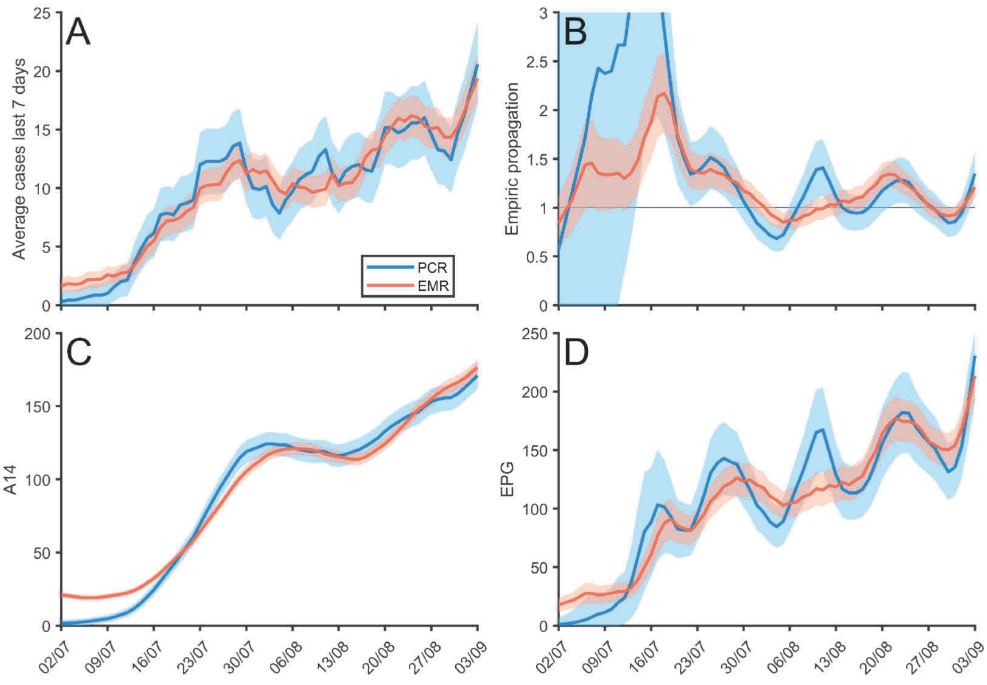

Fig. 42 Weekly average of daily new cases (A), ρ_7 empirical propagation (B), A_{14} attack rate (C) and EPG empirical propagation growth (D) over time for Baix Llobregat Litoral i Sant Boi.

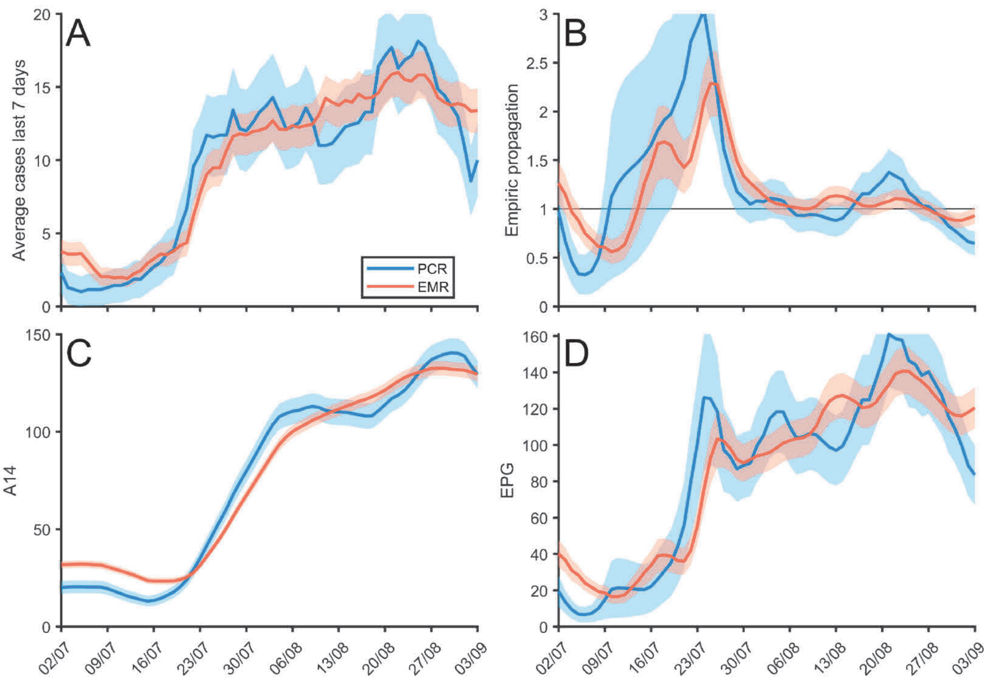

Fig. 43 Weekly average of daily new cases (A), ρ_7 empirical propagation (B), A_{14} attack rate (C) and EPG empirical propagation growth (D) over time for Baix Llobregat Nord.

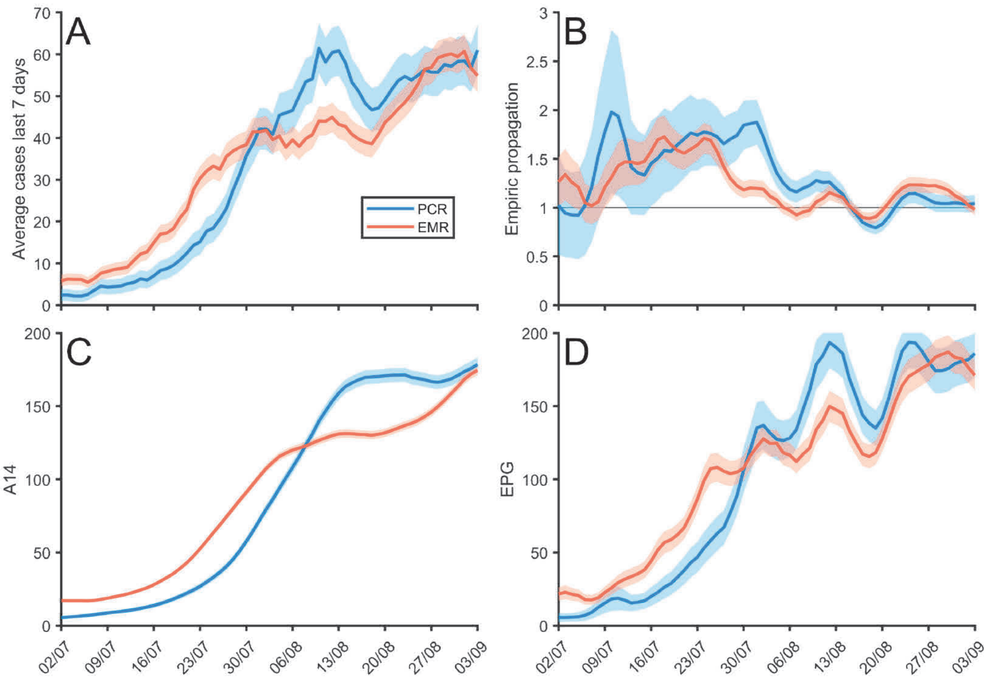

Fig. 44 Weekly average of daily new cases (A), ρ_7 empirical propagation (B), A_{14} attack rate (C) and EPG empirical propagation growth (D) over time for Vallès Occidental Oest.

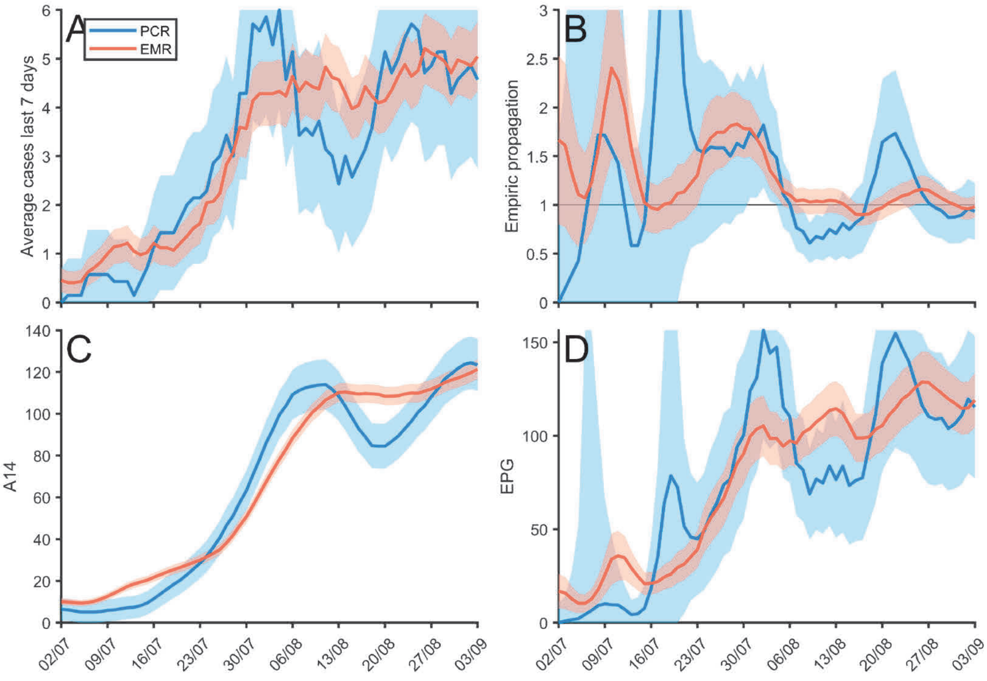

Fig. 45 Weekly average of daily new cases (A), ρ_7 empirical propagation (B), A_{14} attack rate (C) and EPG empirical propagation growth (D) over time for Baix Montseny.

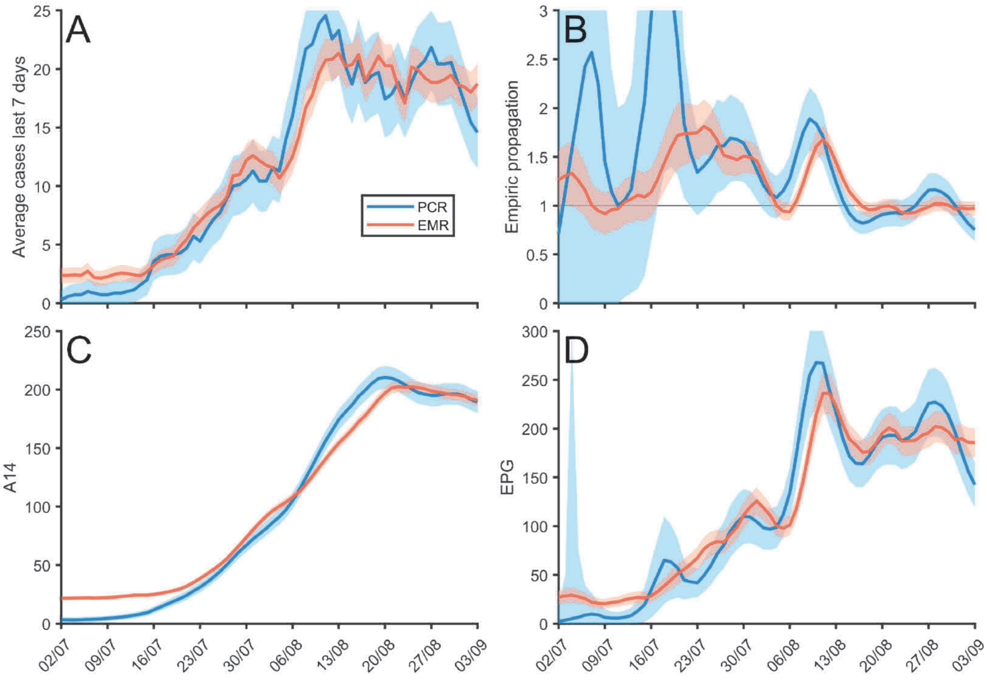

Fig. 46 Weekly average of daily new cases (A), ρ_7 empirical propagation (B), A_{14} attack rate (C) and EPG empirical propagation growth (D) over time for Baix Vallès.

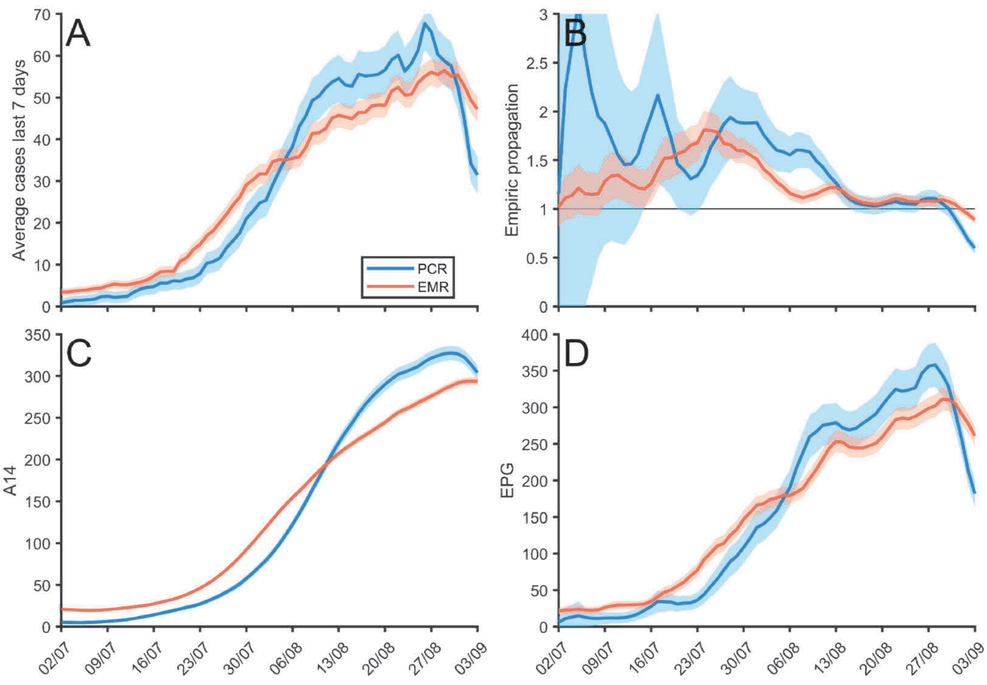

Fig. 47 Weekly average of daily new cases (A), ρ_7 empirical propagation (B), A_{14} attack rate (C) and EPG empirical propagation growth (D) over time for Vallès Oriental Central.

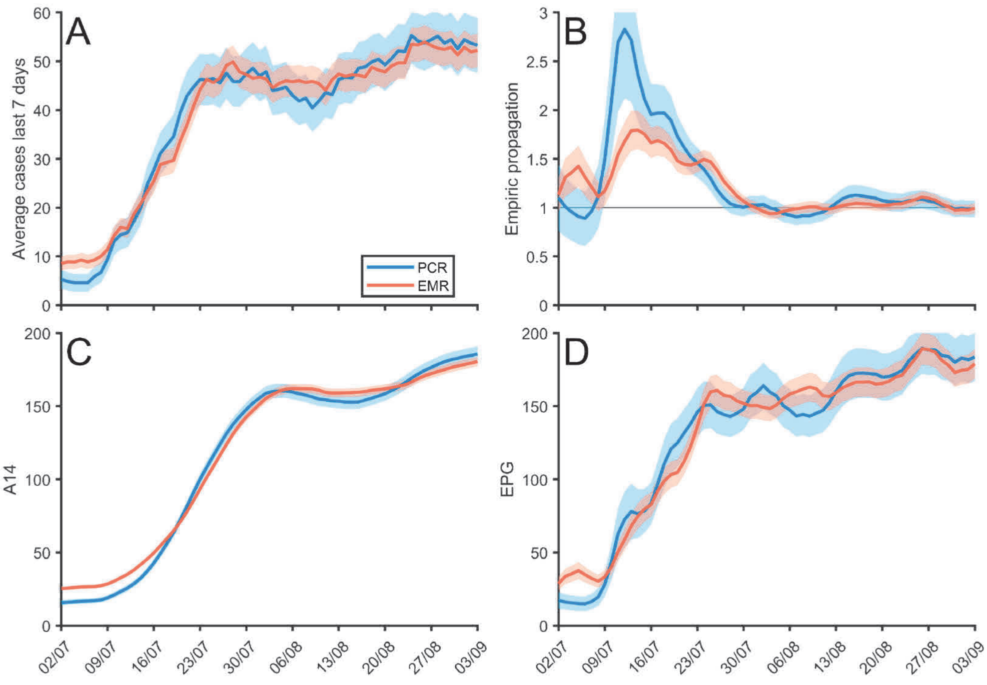

Fig. 48 Weekly average of daily new cases (A), ρ_7 empirical propagation (B), A_{14} attack rate (C) and EPG empirical propagation growth (D) over time for Barcelona Dreta.

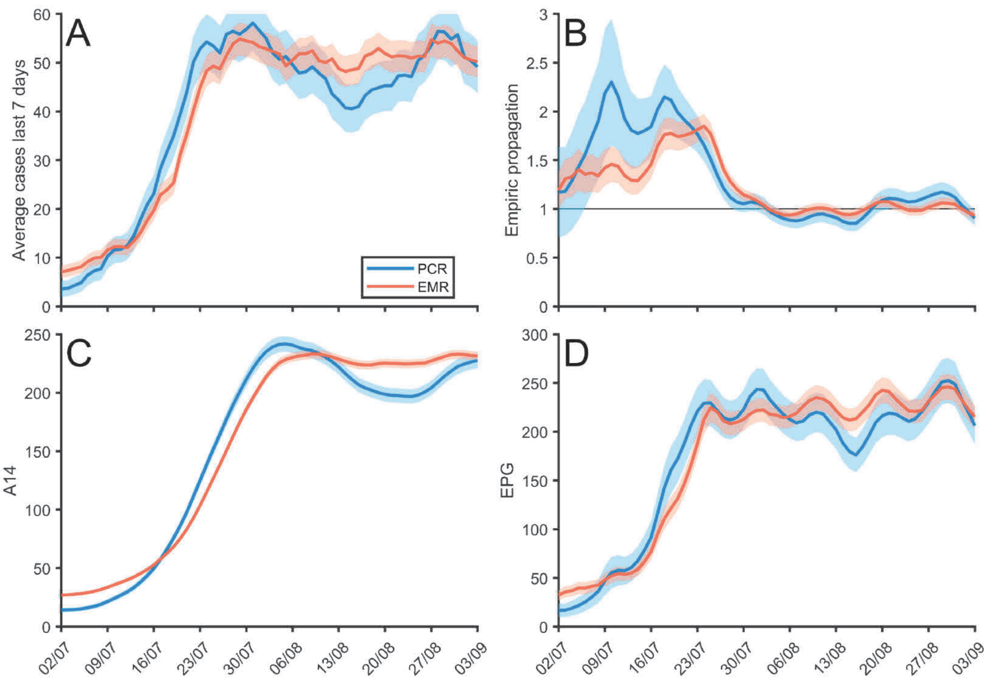

Fig. 49 Weekly average of daily new cases (A), ρ_7 empirical propagation (B), A_{14} attack rate (C) and EPG empirical propagation growth (D) over time for Barcelona Litoral Mar.

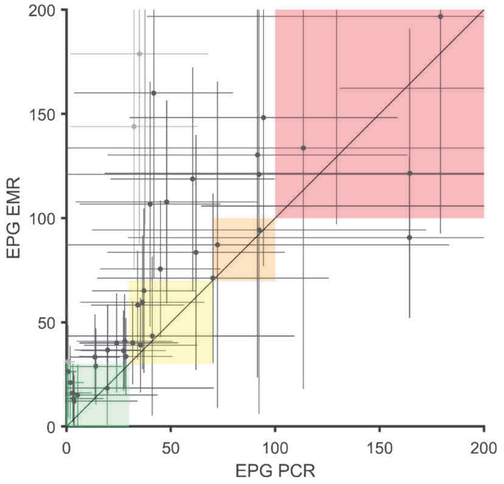

Fig. 51 PCR- and EMR-EPG risk. Coincidences are marked with dark gray and not coincidences in light gray. EPG measured between July 1st and August 31st. EPG for CMA: Alt Urgell.

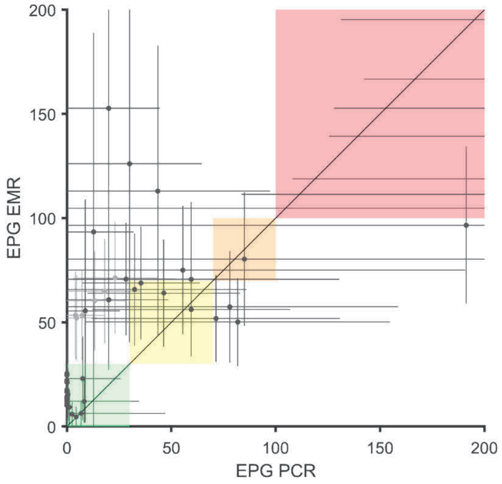

Fig. 52 PCR- and EMR-EPG risk. Coincidences are marked with dark gray and not coincidences in light gray. EPG measured between July 1st and August 31st. EPG for CMA: Cerdanya.

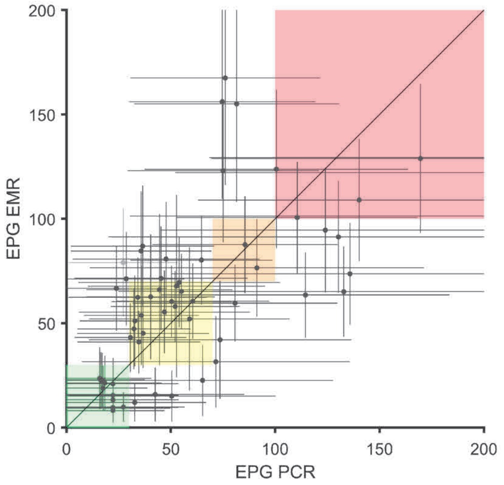

Fig. 53 PCR- and EMR-EPG risk. Coincidences are marked with dark gray and not coincidences in light gray. EPG measured between July 1st and August 31st. EPG for CMA: Pallars.

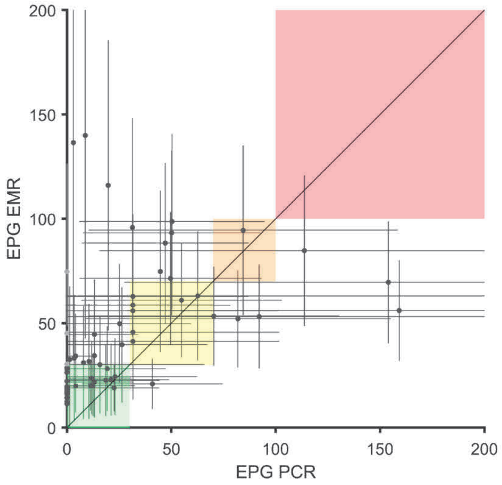

Fig. 54 PCR- and EMR-EPG risk. Coincidences are marked with dark gray and not coincidences in light gray. EPG measured between July 1st and August 31st. EPG for CMA: Aran.

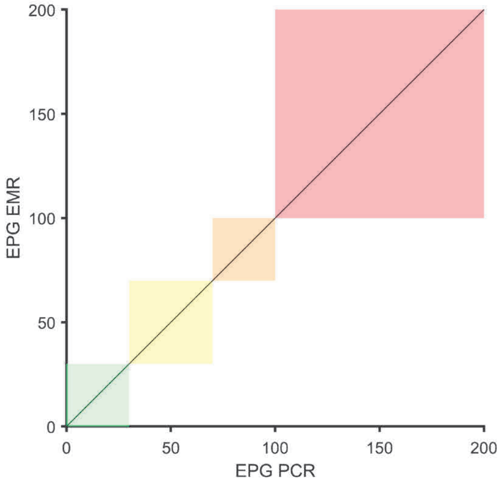

Fig. 55 PCR- and EMR-EPG risk. Coincidences are marked with dark gray and not coincidences in light gray. EPG measured between July 1st and August 31st. EPG for CMA: Lleida.

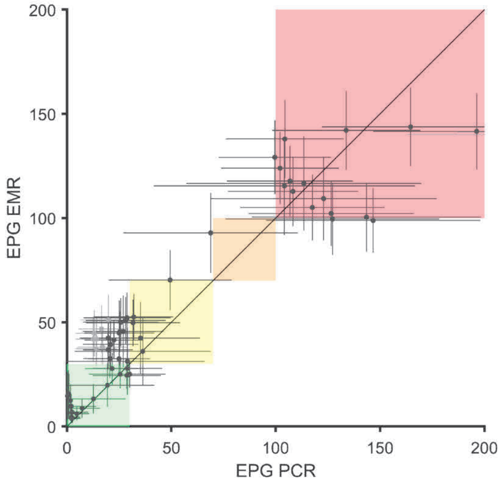

Fig. 56 PCR- and EMR-EPG risk. Coincidences are marked with dark gray and not coincidences in light gray. EPG measured between July 1st and August 31st. EPG for CMA: Alt Camp i Conca de Barberà.

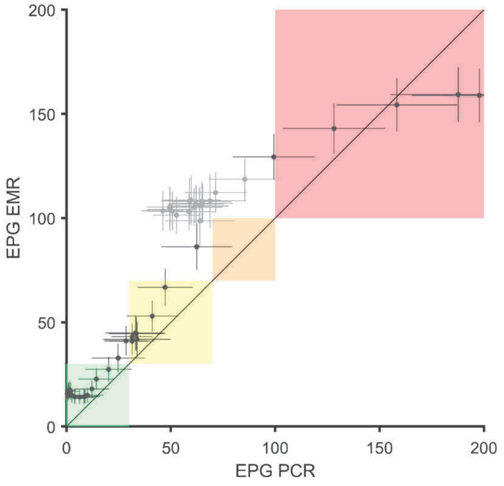

Fig. 57 PCR- and EMR-EPG risk. Coincidences are marked with dark gray and not coincidences in light gray. EPG measured between July 1st and August 31st. EPG for CMA: Baix Camp i Priorat.

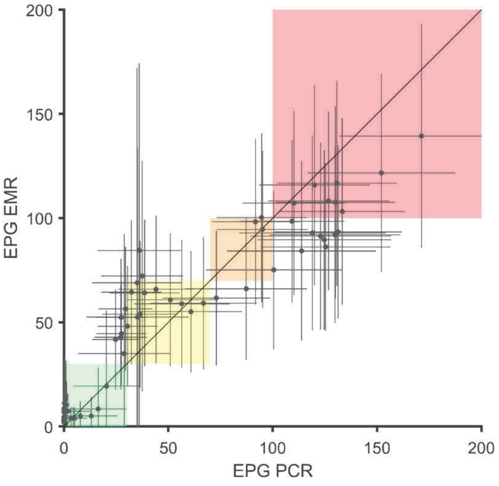

Fig. 58 PCR- and EMR-EPG risk. Coincidences are marked with dark gray and not coincidences in light gray. EPG measured between July 1st and August 31st. EPG for CMA: Baix Penedès.

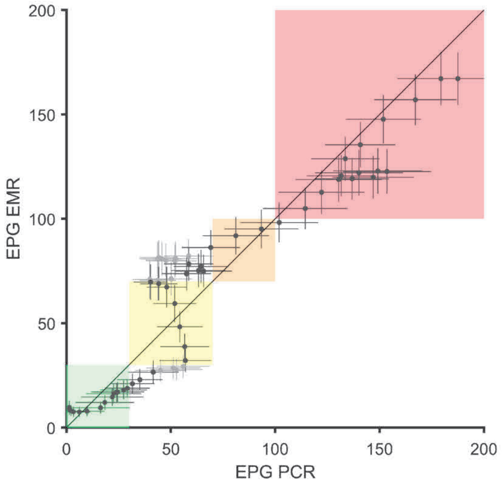

Fig. 59 PCR- and EMR-EPG risk. Coincidences are marked with dark gray and not coincidences in light gray. EPG measured between July 1st and August 31st. EPG for CMA: Tarragonès.

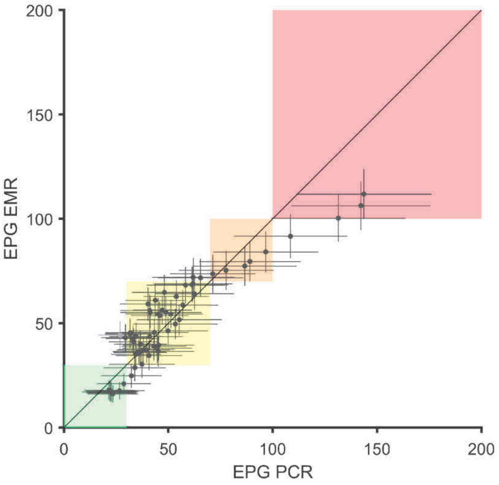

Fig. 60 PCR- and EMR-EPG risk. Coincidences are marked with dark gray and not coincidences in light gray. EPG measured between July 1st and August 31st. EPG for CMA: Anoia.

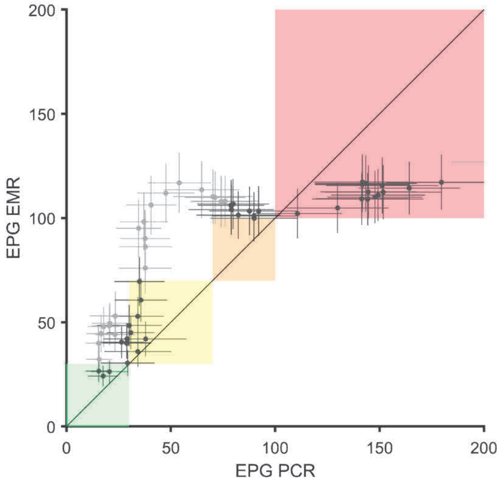

Fig. 61 PCR- and EMR-EPG risk. Coincidences are marked with dark gray and not coincidences in light gray. EPG measured between July 1st and August 31st. EPG for CMA: Osona.

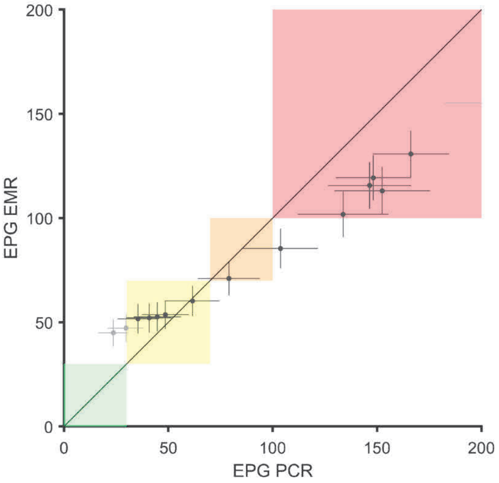

Fig. 62 PCR- and EMR-EPG risk. Coincidences are marked with dark gray and not coincidences in light gray. EPG measured between July 1st and August 31st. EPG for CMA: Baix Llobregat Centre i Fontsanta -L'H N.

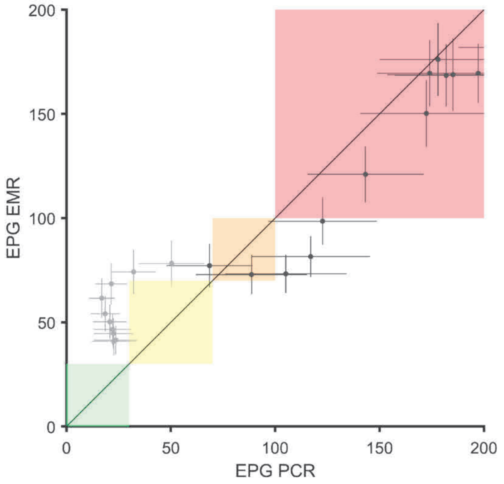

Fig. 63 PCR- and EMR-EPG risk. Coincidences are marked with dark gray and not coincidences in light gray. EPG measured between July 1st and August 31st. EPG for CMA: L'Hospitalet Sud i el Prat de Llobregat.

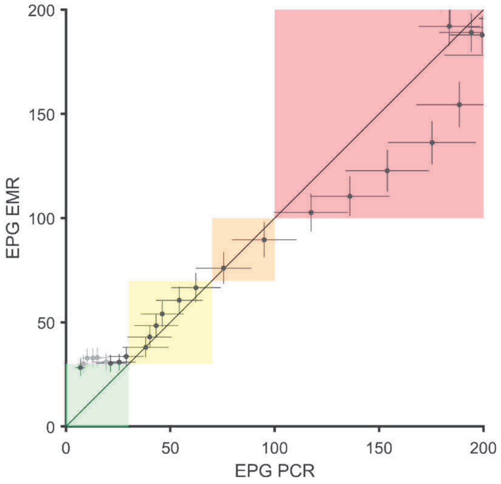

Fig. 64 PCR- and EMR-EPG risk. Coincidences are marked with dark gray and not coincidences in light gray. EPG measured between July 1st and August 31st. EPG for CMA: Barcelonès Nord i Baix Maresme.

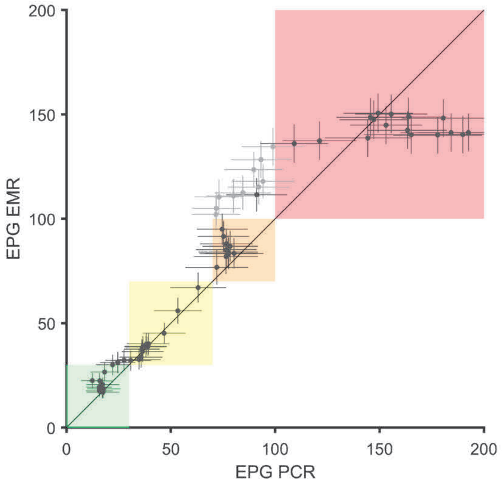

Fig. 65 PCR- and EMR-EPG risk. Coincidences are marked with dark gray and not coincidences in light gray. EPG measured between July 1st and August 31st. EPG for CMA: Maresme Central.

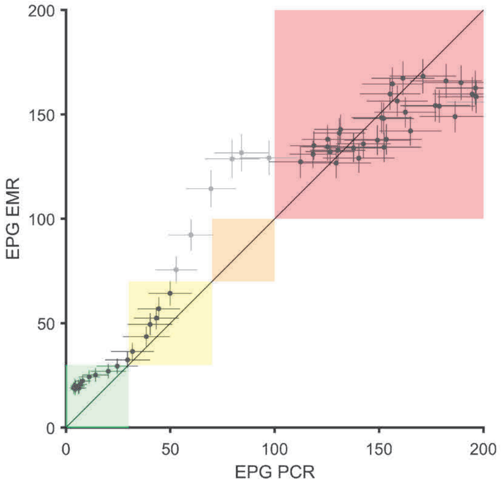

Fig. 66 PCR- and EMR-EPG risk. Coincidences are marked with dark gray and not coincidences in light gray. EPG measured between July 1st and August 31st. EPG for CMA: Vallès Occidental Est.

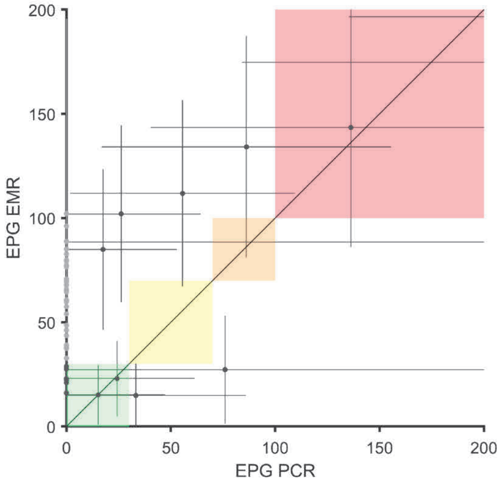

Fig. 67 PCR- and EMR-EPG risk. Coincidences are marked with dark gray and not coincidences in light gray. EPG measured between July 1st and August 31st. EPG for CMA: Alta Ribagorça.

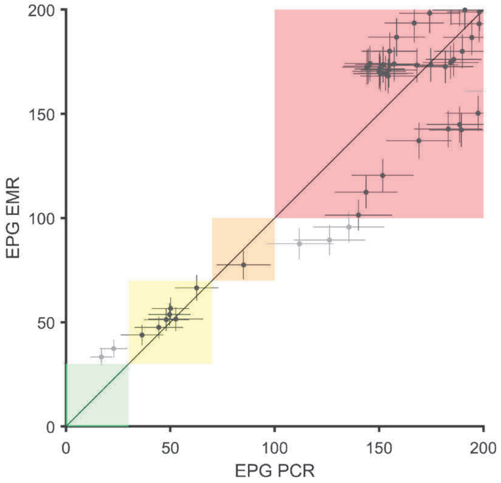

Fig. 68 PCR- and EMR-EPG risk. Coincidences are marked with dark gray and not coincidences in light gray. EPG measured between July 1st and August 31st. EPG for CMA: Barcelona Esquerra.

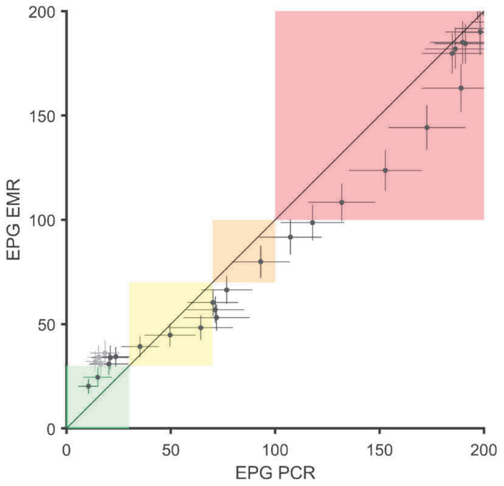

Fig. 69 PCR- and EMR-EPG risk. Coincidences are marked with dark gray and not coincidences in light gray. EPG measured between July 1st and August 31st. EPG for CMA: Barcelona Nord.

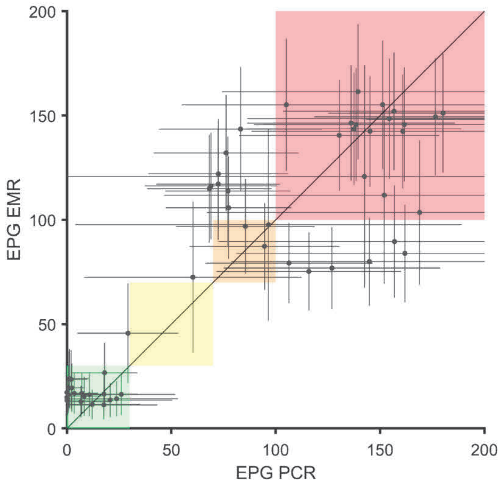

Fig. 70 PCR- and EMR-EPG risk. Coincidences are marked with dark gray and not coincidences in light gray. EPG measured between July 1st and August 31st. EPG for CMA: Altebrat.

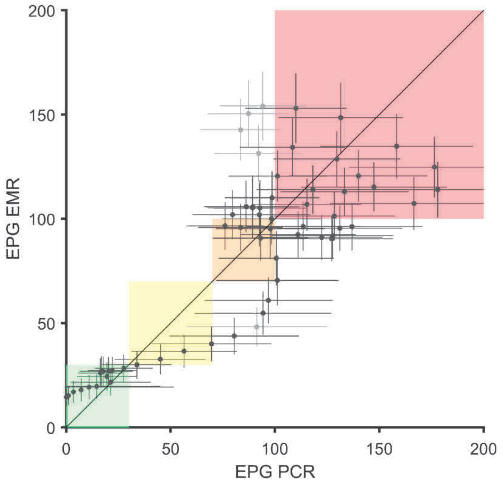

Fig. 71 PCR- and EMR-EPG risk. Coincidences are marked with dark gray and not coincidences in light gray. EPG measured between July 1st and August 31st. EPG for CMA: Baix Ebre.

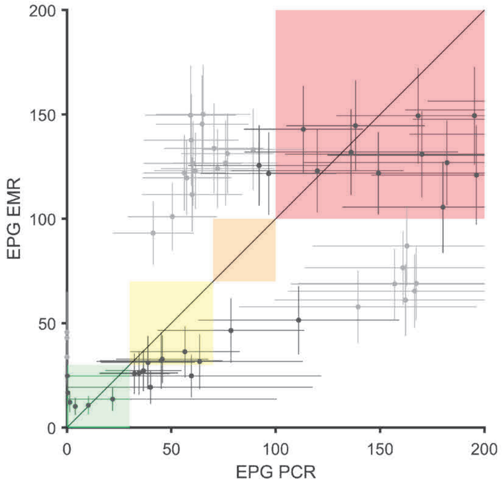

Fig. 72 PCR- and EMR-EPG risk. Coincidences are marked with dark gray and not coincidences in light gray. EPG measured between July 1st and August 31st. EPG for CMA: Montsià.

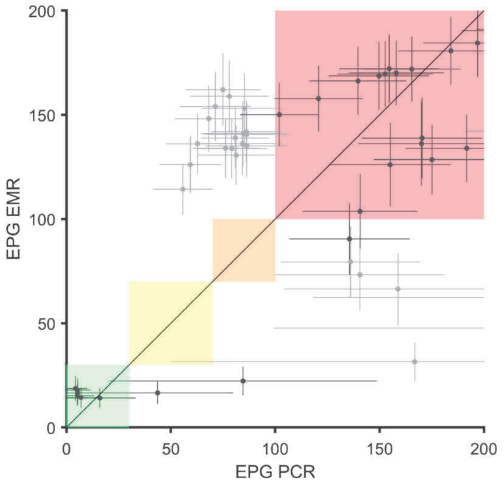

Fig. 73 PCR- and EMR-EPG risk. Coincidences are marked with dark gray and not coincidences in light gray. EPG measured between July 1st and August 31st. EPG for CMA: Alt Empordà.

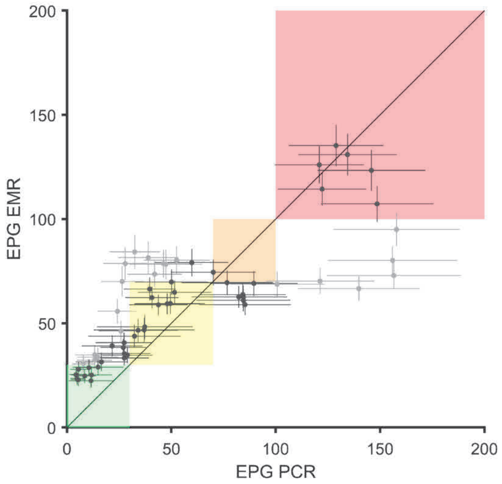

Fig. 74 PCR- and EMR-EPG risk. Coincidences are marked with dark gray and not coincidences in light gray. EPG measured between July 1st and August 31st. EPG for CMA: Baix Empordà.

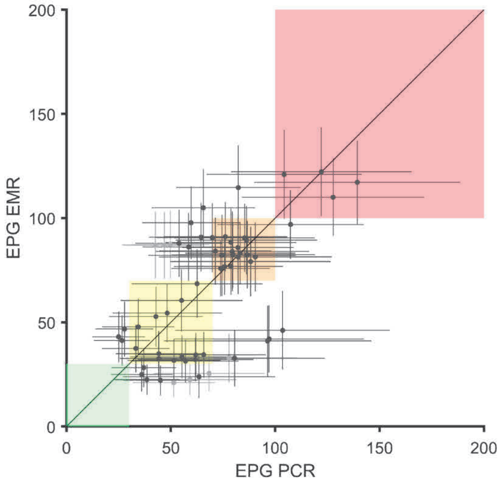

Fig. 75 PCR- and EMR-EPG risk. Coincidences are marked with dark gray and not coincidences in light gray. EPG measured between July 1st and August 31st. EPG for CMA: Garrotxa.

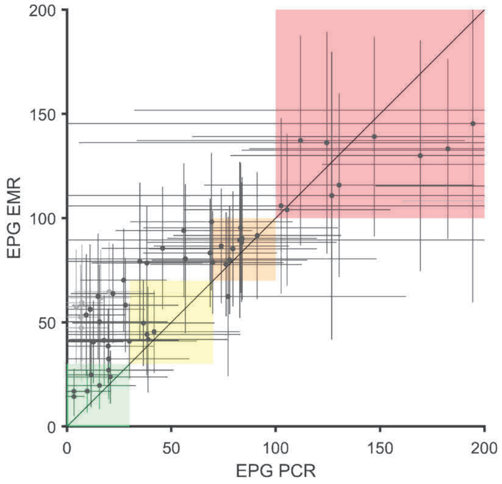

Fig. 76 PCR- and EMR-EPG risk. Coincidences are marked with dark gray and not coincidences in light gray. EPG measured between July 1st and August 31st. EPG for CMA: Ripollès.

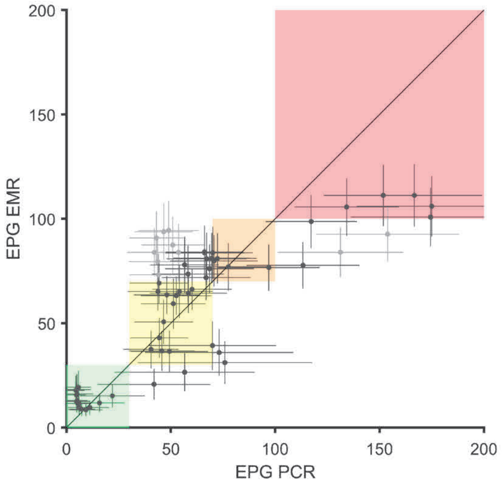

Fig. 77 PCR- and EMR-EPG risk. Coincidences are marked with dark gray and not coincidences in light gray. EPG measured between July 1st and August 31st. EPG for CMA: Alt Maresme.

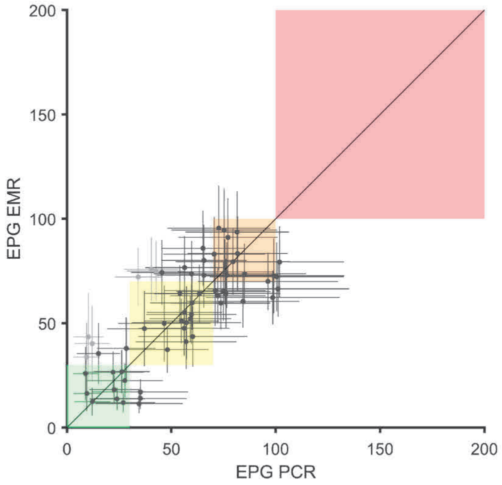

Fig. 78 PCR- and EMR-EPG risk. Coincidences are marked with dark gray and not coincidences in light gray. EPG measured between July 1st and August 31st. EPG for CMA: Selva Marítima.

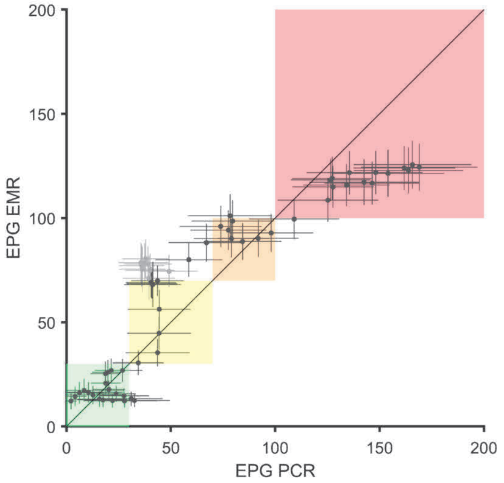

Fig. 79 PCR- and EMR-EPG risk. Coincidences are marked with dark gray and not coincidences in light gray. EPG measured between July 1st and August 31st. EPG for CMA: Gironès Nord i Pla de l'Estany.

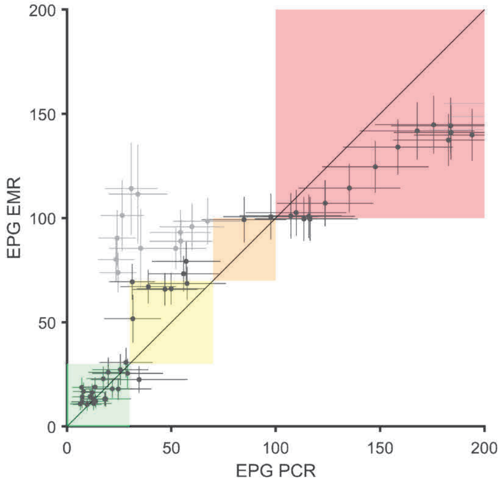

Fig. 80 PCR- and EMR-EPG risk. Coincidences are marked with dark gray and not coincidences in light gray. EPG measured between July 1st and August 31st. EPG for CMA: Gironès Sud i Selva Interior.

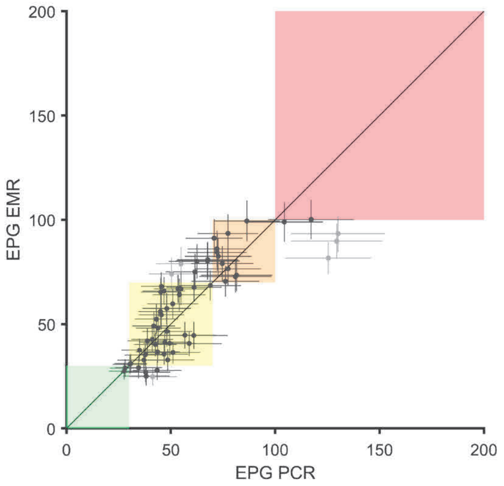

Fig. 81 PCR- and EMR-EPG risk. Coincidences are marked with dark gray and not coincidences in light gray. EPG measured between July 1st and August 31st. EPG for CMA: Bages i Solsonès.

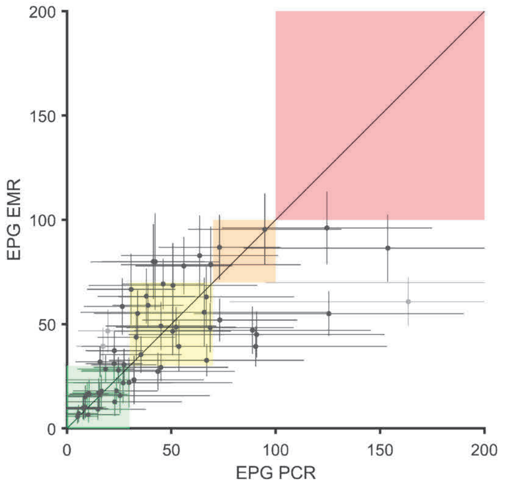

Fig. 82 PCR- and EMR-EPG risk. Coincidences are marked with dark gray and not coincidences in light gray. EPG measured between July 1st and August 31st. EPG for CMA: Berguedà.

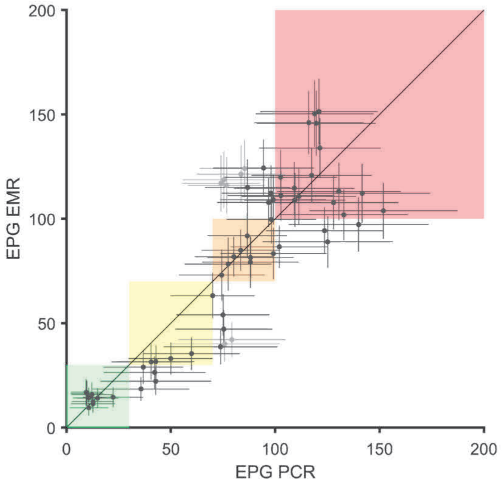

Fig. 83 PCR- and EMR-EPG risk. Coincidences are marked with dark gray and not coincidences in light gray. EPG measured between July 1st and August 31st. EPG for CMA: Alt Penedès.

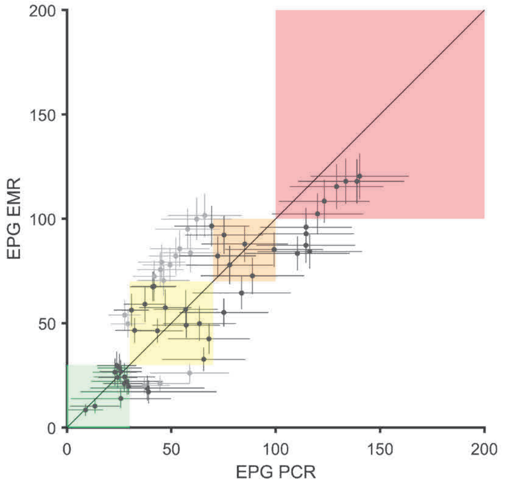

Fig. 84 PCR- and EMR-EPG risk. Coincidences are marked with dark gray and not coincidences in light gray. EPG measured between July 1st and August 31st. EPG for CMA: Garraf.

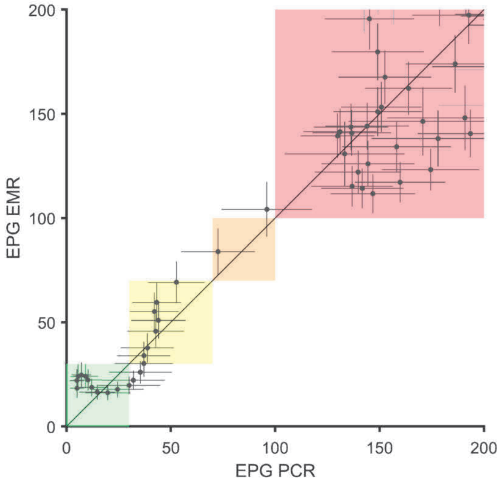

Fig. 85 PCR- and EMR-EPG risk. Coincidences are marked with dark gray and not coincidences in light gray. EPG measured between July 1st and August 31st. EPG for CMA: Baix Llobregat Litoral i Viladecans.

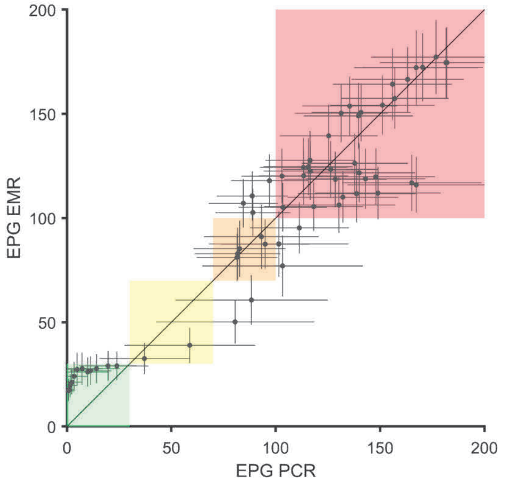

Fig. 86 PCR- and EMR-EPG risk. Coincidences are marked with dark gray and not coincidences in light gray. EPG measured between July 1st and August 31st. EPG for CMA: Baix Llobregat Litoral i Sant Boi.

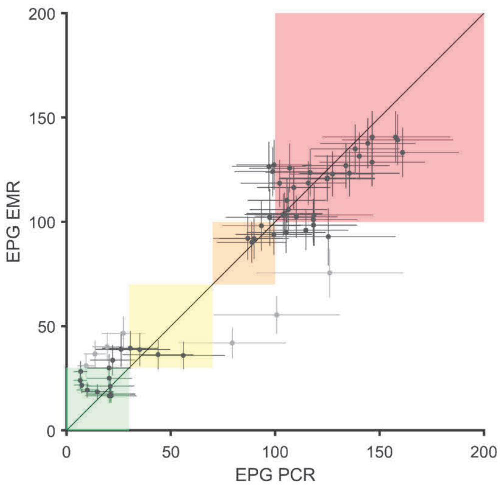

Fig. 87 PCR- and EMR-EPG risk. Coincidences are marked with dark gray and not coincidences in light gray. EPG measured between July 1st and August 31st. EPG for CMA: Baix Llobregat Nord.

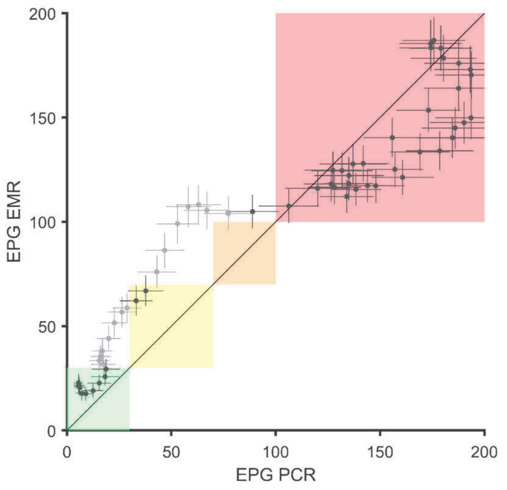

Fig. 88 PCR- and EMR-EPG risk. Coincidences are marked with dark gray and not coincidences in light gray. EPG measured between July 1st and August 31st. EPG for CMA: Vallès Occidental Oest.

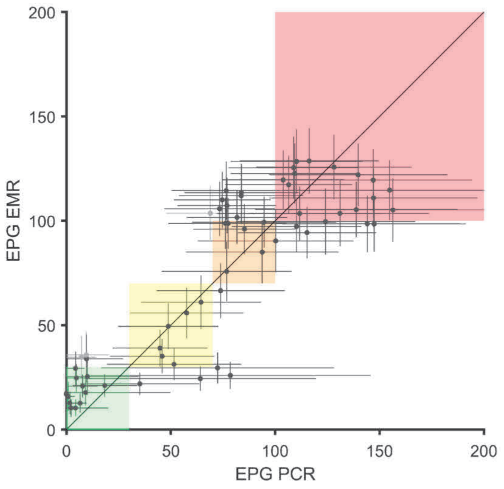

Fig. 89 PCR- and EMR-EPG risk. Coincidences are marked with dark gray and not coincidences in light gray. EPG measured between July 1st and August 31st. EPG for CMA: Baix Montseny.

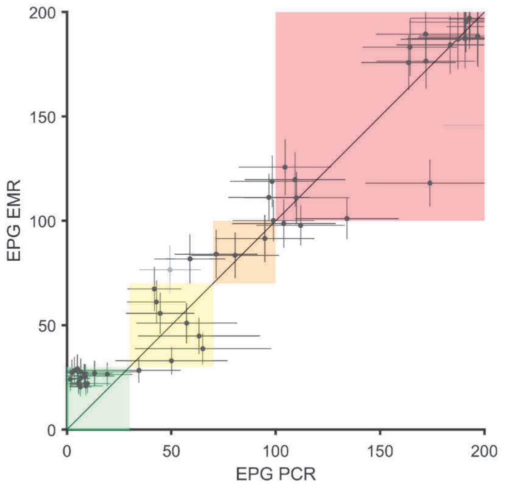

Fig. 90 PCR- and EMR-EPG risk. Coincidences are marked with dark gray and not coincidences in light gray. EPG measured between July 1st and August 31st. EPG for CMA: Baix Vallès.

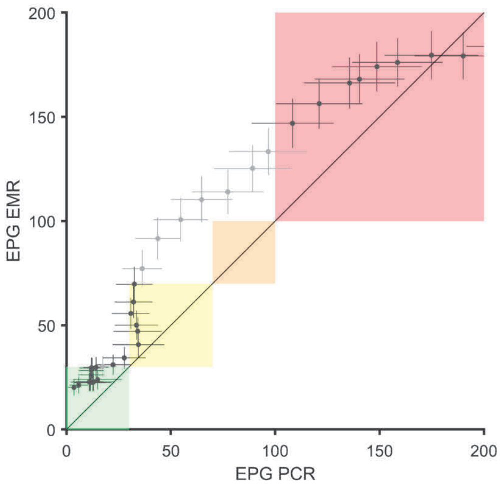

Fig. 91 PCR- and EMR-EPG risk. Coincidences are marked with dark gray and not coincidences in light gray. EPG measured between July 1st and August 31st. EPG for CMA: Vallès Oriental Central.

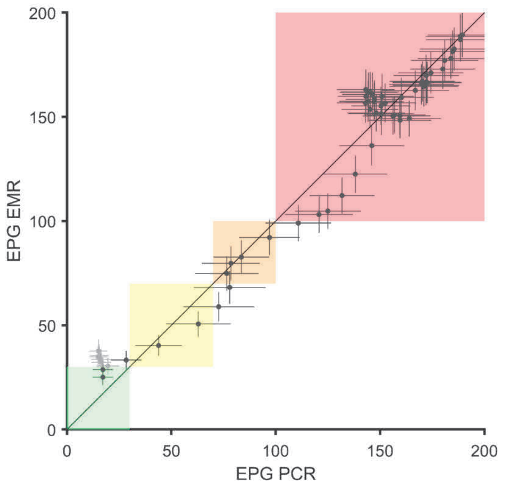

Fig. 92 PCR- and EMR-EPG risk. Coincidences are marked with dark gray and not coincidences in light gray. EPG measured between July 1st and August 31st. EPG for CMA: Barcelona Dreta.

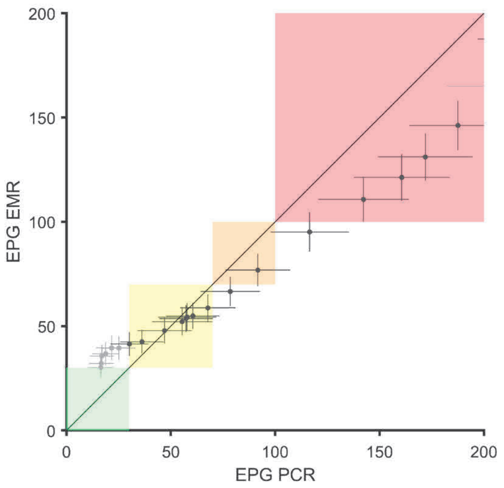

Fig. 93 PCR- and EMR-EPG risk. Coincidences are marked with dark gray and not coincidences in light gray. EPG measured between July 1st and August 31st. EPG for CMA: Barcelona Litoral Mar.

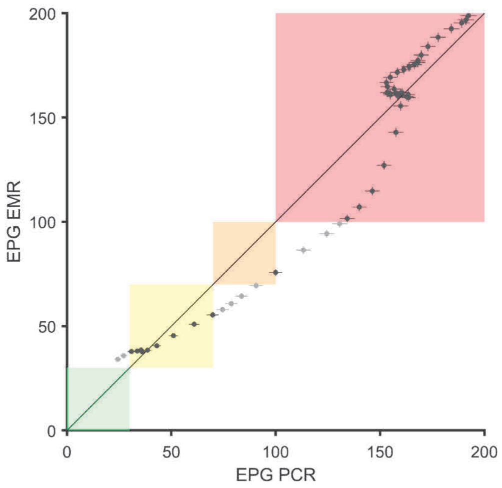

Fig. 94 PCR- and EMR-EPG risk. Coincidences are marked with dark gray and not coincidences in light gray. EPG measured between July 1st and August 31st. EPG for CMA: CATALUNYA.

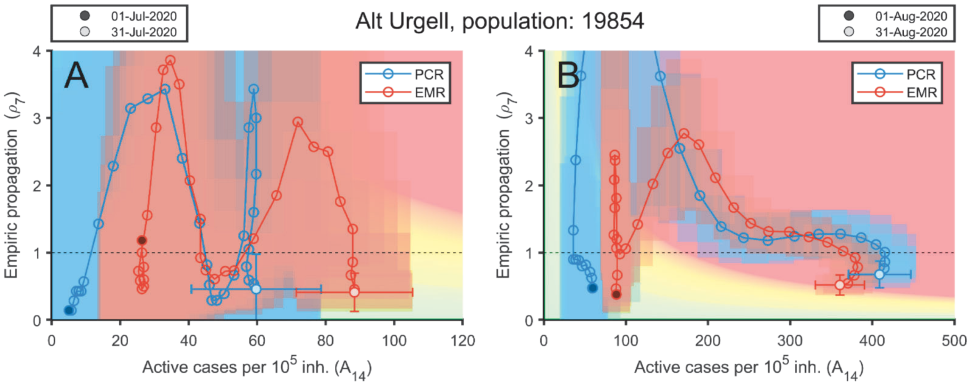

Fig. 95 Risk diagram for the evolution of the COVID-19 pandemic in Alt Urgell based on EMR (red) and on PCR (blue) cases for the month of July (left) and August 2020 (right).

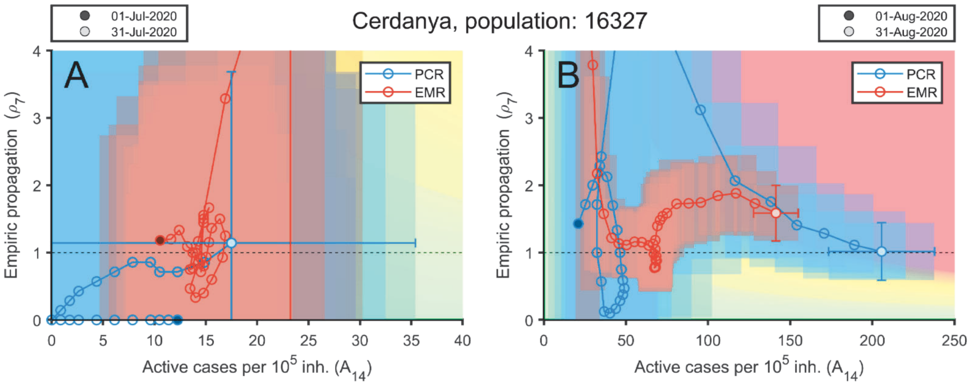

Fig. 96 Risk diagram for the evolution of the COVID-19 pandemic in Cerdanya based on EMR (red) and on PCR (blue) cases for the month of July (left) and August 2020 (right).

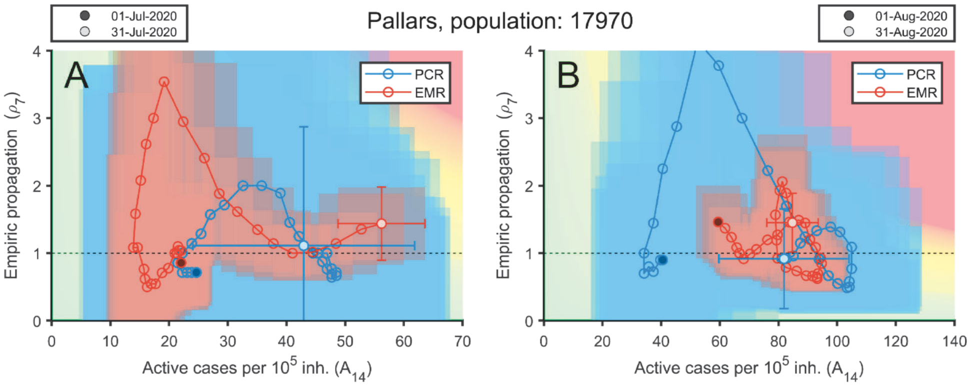

Fig. 97 Risk diagram for the evolution of the COVID-19 pandemic in Pallars based on EMR (red) and on PCR (blue) cases for the month of July (left) and August 2020 (right).

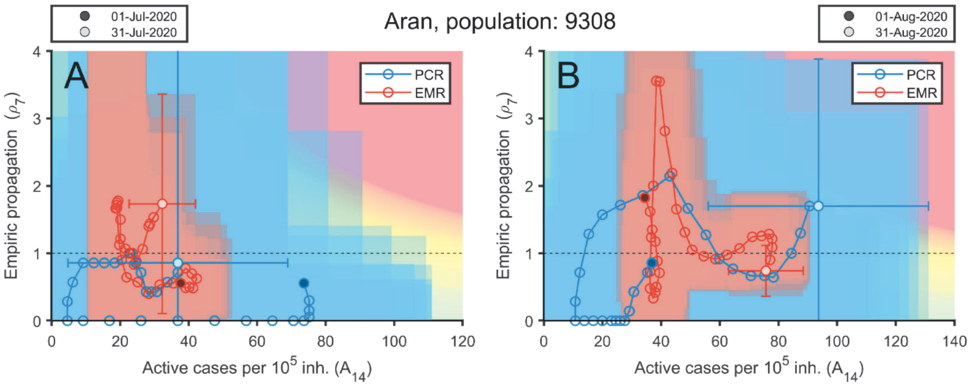

Fig. 98 Risk diagram for the evolution of the COVID-19 pandemic in Aran based on EMR (red) and on PCR (blue) cases for the month of July (left) and August 2020 (right).

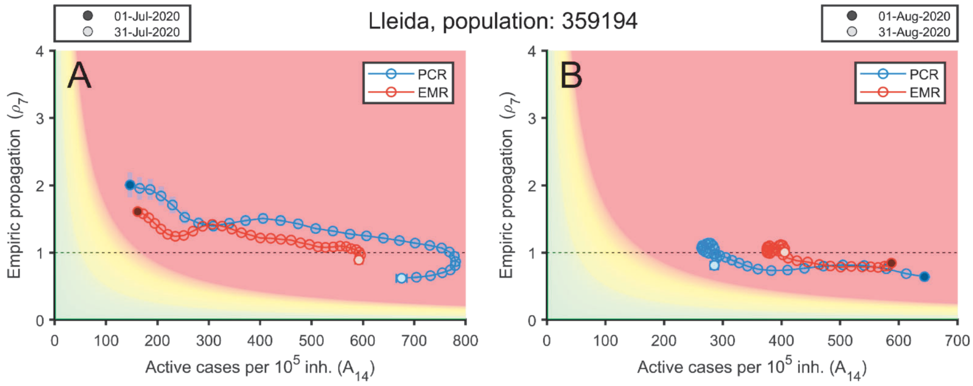

Fig. 99 Risk diagram for the evolution of the COVID-19 pandemic in Lleida based on EMR (red) and on PCR (blue) cases for the month of July (left) and August 2020 (right).

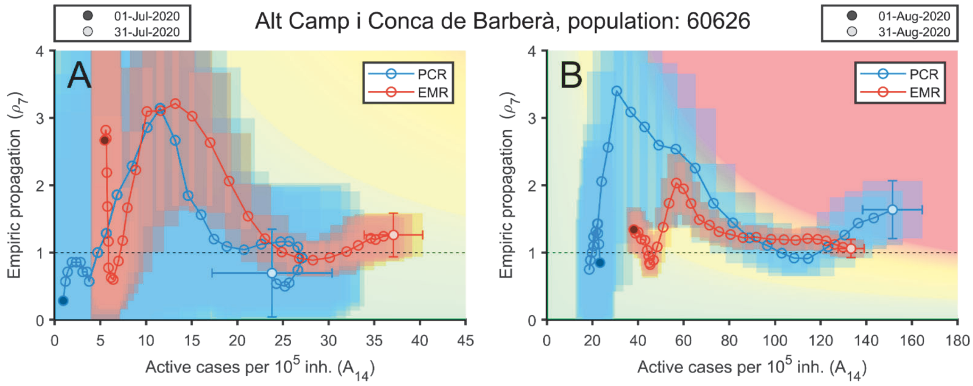

Fig. 100 Risk diagram for the evolution of the COVID-19 pandemic in Alt Camp i Conca de Barberà based on EMR (red) and on PCR (blue) cases for the month of July (left) and August 2020 (right).

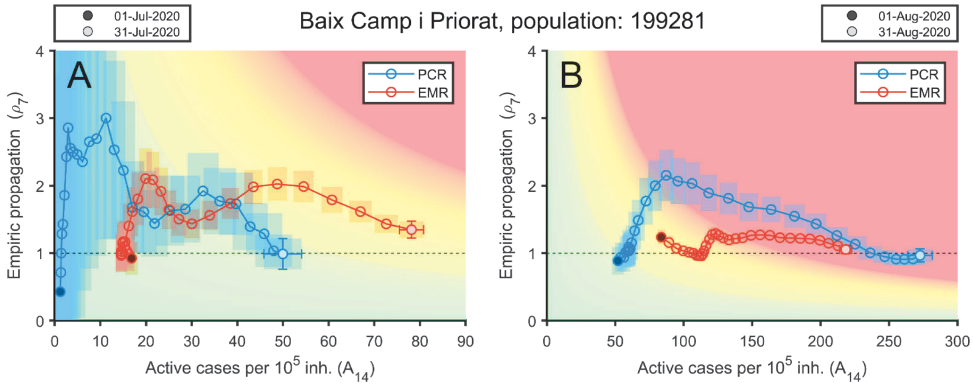

Fig. 101 Risk diagram for the evolution of the COVID-19 pandemic in Baix Camp i Priorat based on EMR (red) and on PCR (blue) cases for the month of July (left) and August 2020 (right).

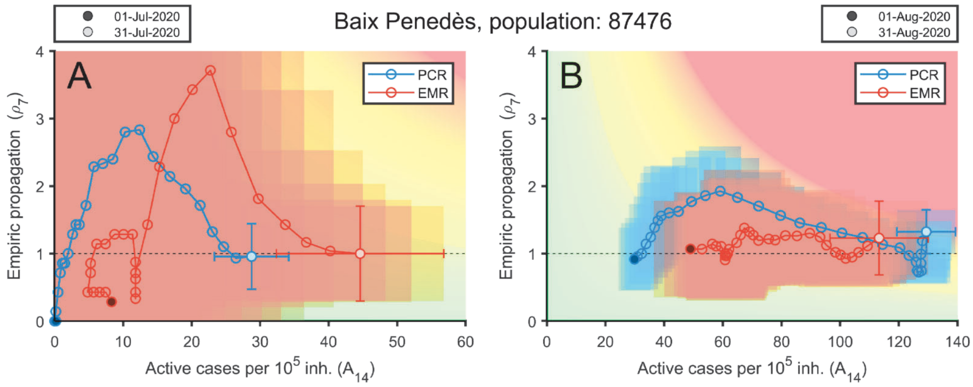

Fig. 102 Risk diagram for the evolution of the COVID-19 pandemic in Baix Penedès based on EMR (red) and on PCR (blue) cases for the month of July (left) and August 2020 (right).

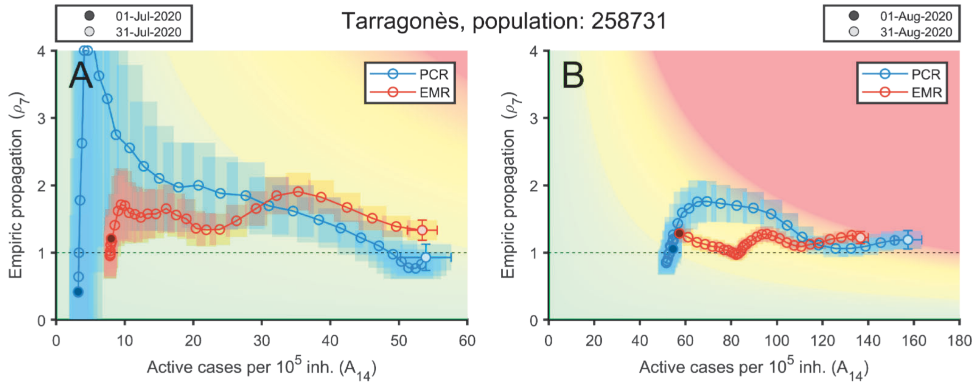

Fig. 103 Risk diagram for the evolution of the COVID-19 pandemic in Tarragonès based on EMR (red) and on PCR (blue) cases for the month of July (left) and August 2020 (right).

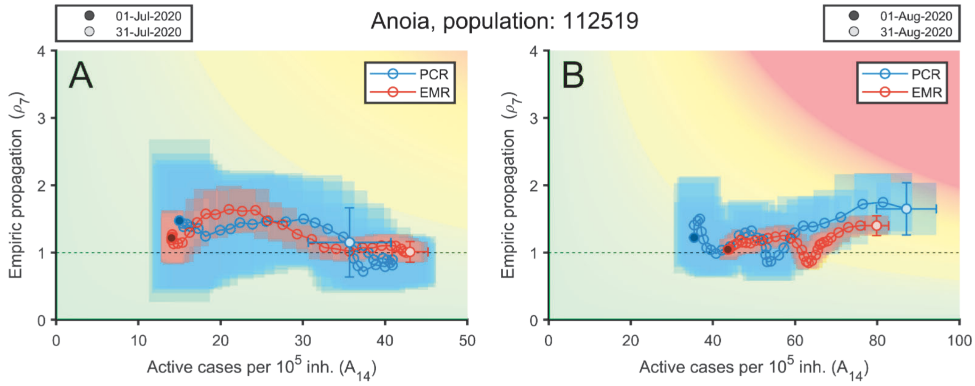

Fig. 104 Risk diagram for the evolution of the COVID-19 pandemic in Anoia based on EMR (red) and on PCR (blue) cases for the month of July (left) and August 2020 (right).

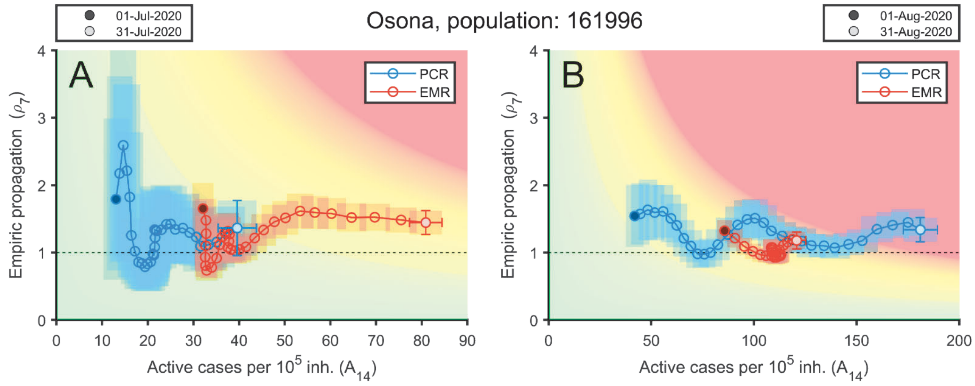

Fig. 105 Risk diagram for the evolution of the COVID-19 pandemic in Osona based on EMR (red) and on PCR (blue) cases for the month of July (left) and August 2020 (right).

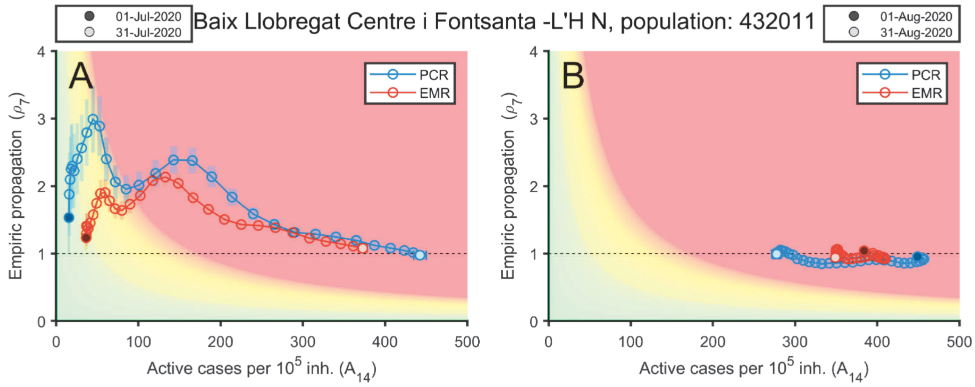

Fig. 106 Risk diagram for the evolution of the COVID-19 pandemic in Baix Llobregat Centre i Fontsanta -L'H N based on EMR (red) and on PCR (blue) cases for the month of July (left) and August 2020 (right).

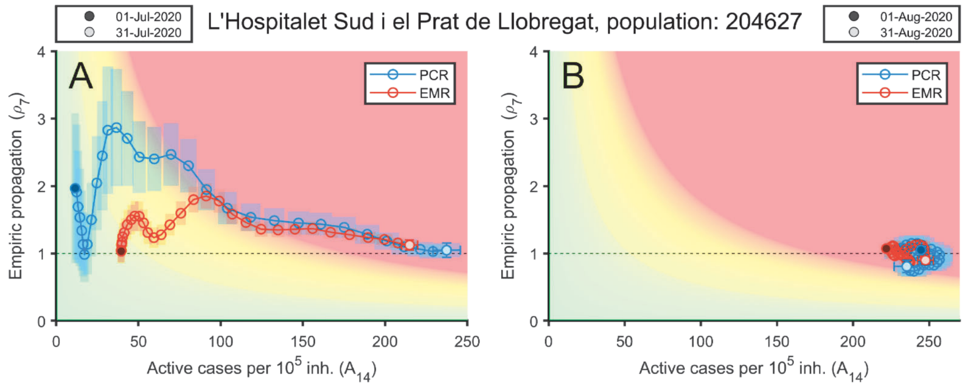

Fig. 107 Risk diagram for the evolution of the COVID-19 pandemic in L'Hospitalet Sud i el Prat de Llobregat based on EMR (red) and on PCR (blue) cases for the month of July (left) and August 2020 (right).

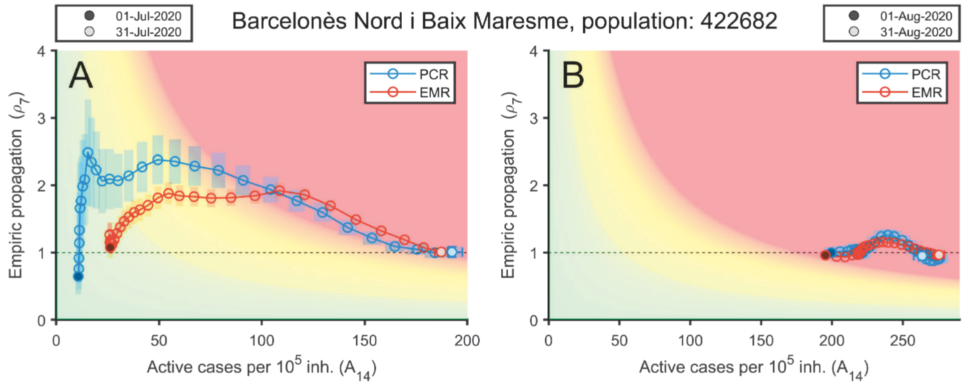

Fig. 108 Risk diagram for the evolution of the COVID-19 pandemic in Barcelonès Nord i Baix Maresme based on EMR (red) and on PCR (blue) cases for the month of July (left) and August 2020 (right).

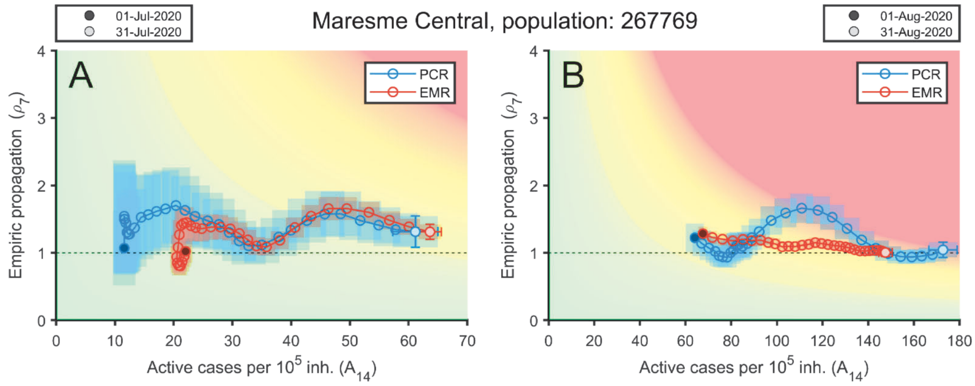

Fig. 109 Risk diagram for the evolution of the COVID-19 pandemic in Maresme Central based on EMR (red) and on PCR (blue) cases for the month of July (left) and August 2020 (right).

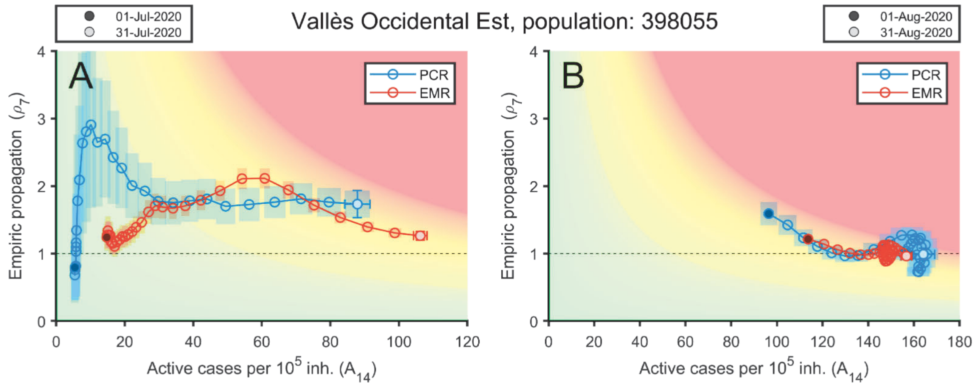

Fig. 110 Risk diagram for the evolution of the COVID-19 pandemic in Vallès Occidental Est based on EMR (red) and on PCR (blue) cases for the month of July (left) and August 2020 (right).

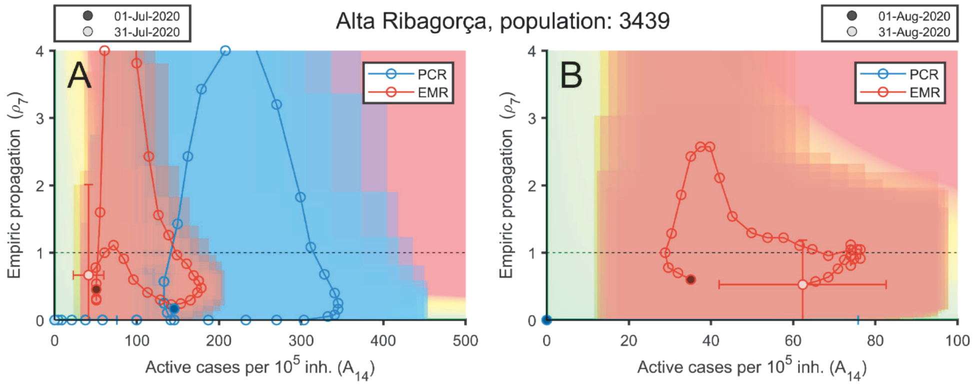

Fig. 111 Risk diagram for the evolution of the COVID-19 pandemic in Alta Ribagorça based on EMR (red) and on PCR (blue) cases for the month of July (left) and August 2020 (right).

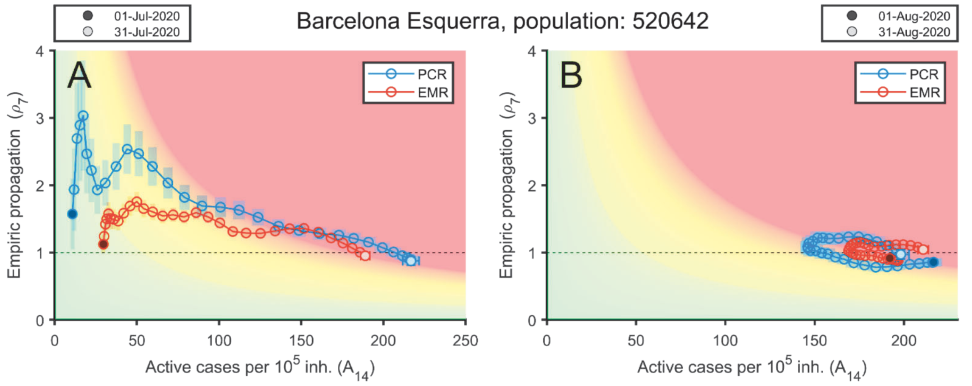

Fig. 112 Risk diagram for the evolution of the COVID-19 pandemic in Barcelona Esquerra based on EMR (red) and on PCR (blue) cases for the month of July (left) and August 2020 (right).

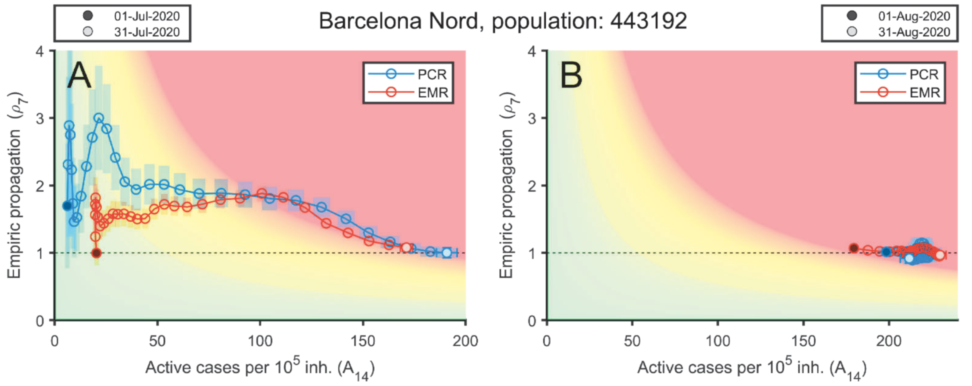

Fig. 113 Risk diagram for the evolution of the COVID-19 pandemic in Barcelona Nord based on EMR (red) and on PCR (blue) cases for the month of July (left) and August 2020 (right).

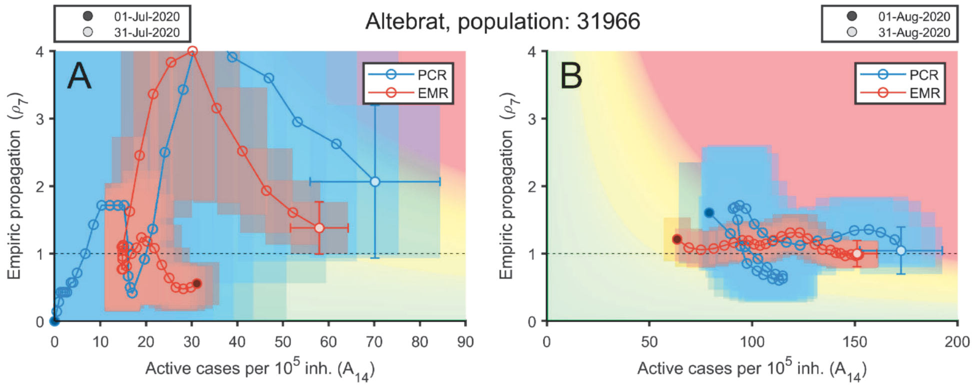

Fig. 114 Risk diagram for the evolution of the COVID-19 pandemic in Altebrat based on EMR (red) and on PCR (blue) cases for the month of July (left) and August 2020 (right).

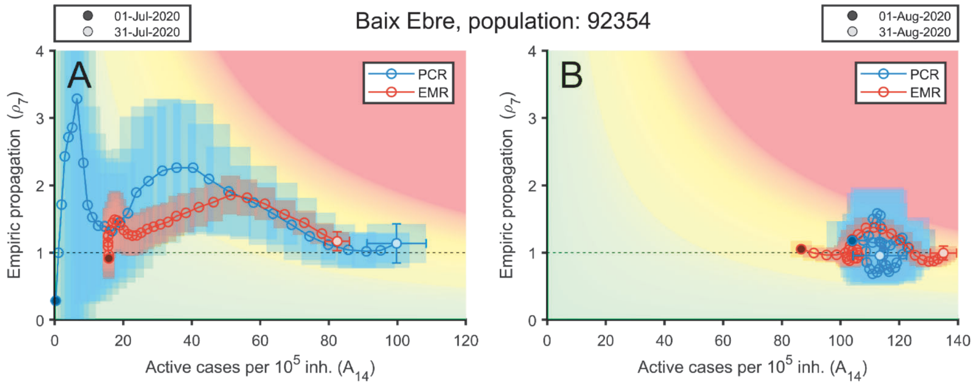

Fig. 115 Risk diagram for the evolution of the COVID-19 pandemic in Baix Ebre based on EMR (red) and on PCR (blue) cases for the month of July (left) and August 2020 (right).

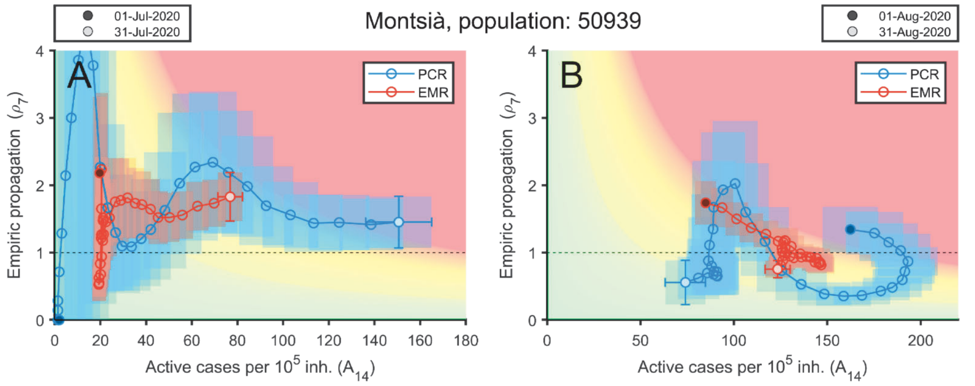

Fig. 116 Risk diagram for the evolution of the COVID-19 pandemic in Montsià based on EMR (red) and on PCR (blue) cases for the month of July (left) and August 2020 (right).

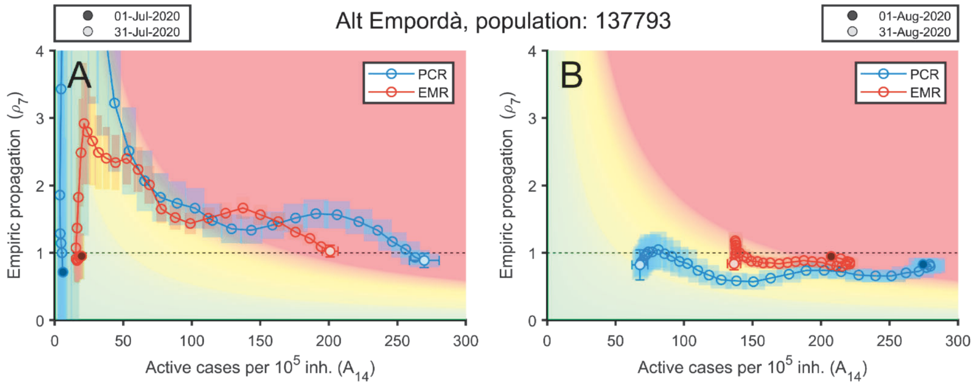

Fig. 117 Risk diagram for the evolution of the COVID-19 pandemic in Alt Empordà based on EMR (red) and on PCR (blue) cases for the month of July (left) and August 2020 (right).

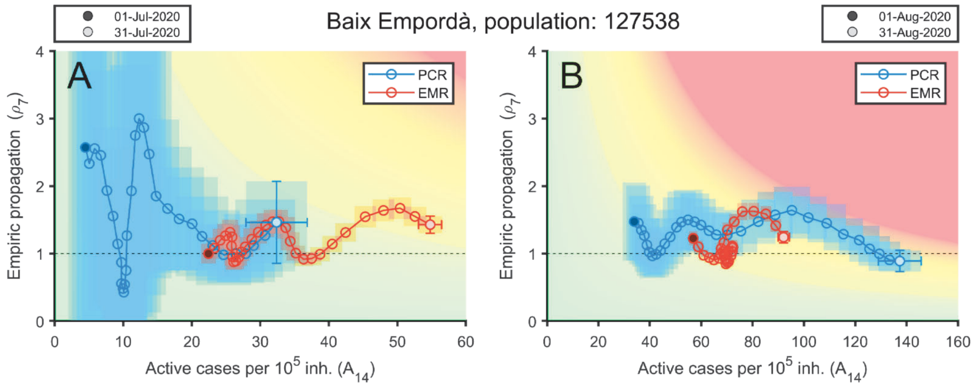

Fig. 118 Risk diagram for the evolution of the COVID-19 pandemic in Baix Empordà based on EMR (red) and on PCR (blue) cases for the month of July (left) and August 2020 (right).

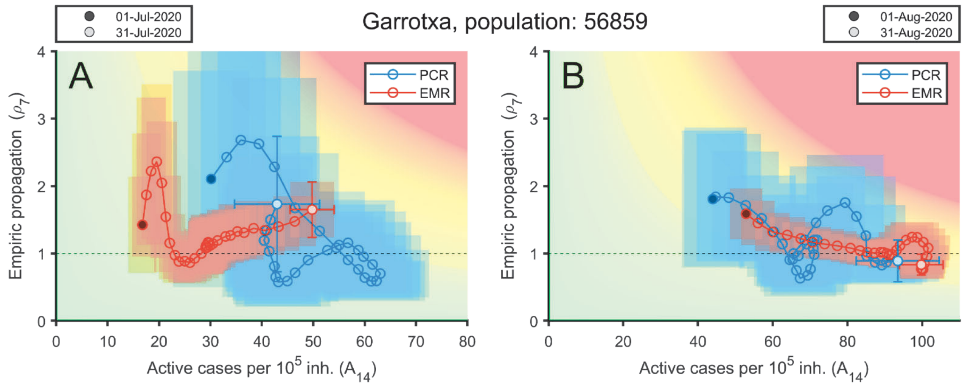

Fig. 119 Risk diagram for the evolution of the COVID-19 pandemic in Garrotxa based on EMR (red) and on PCR (blue) cases for the month of July (left) and August 2020 (right).

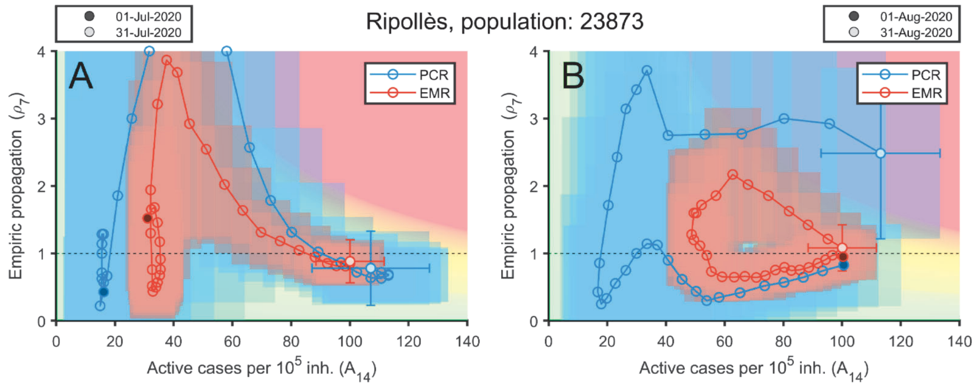

Fig. 120 Risk diagram for the evolution of the COVID-19 pandemic in Ripollès based on EMR (red) and on PCR (blue) cases for the month of July (left) and August 2020 (right).

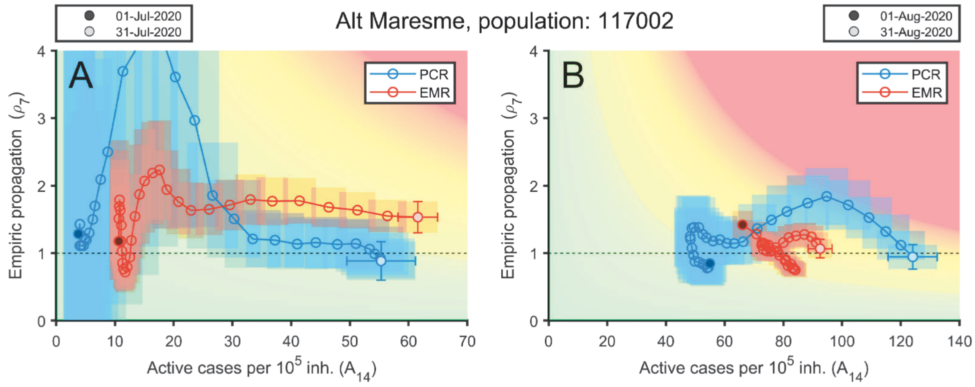

Fig. 121 Risk diagram for the evolution of the COVID-19 pandemic in Alt Maresme based on EMR (red) and on PCR (blue) cases for the month of July (left) and August 2020 (right).

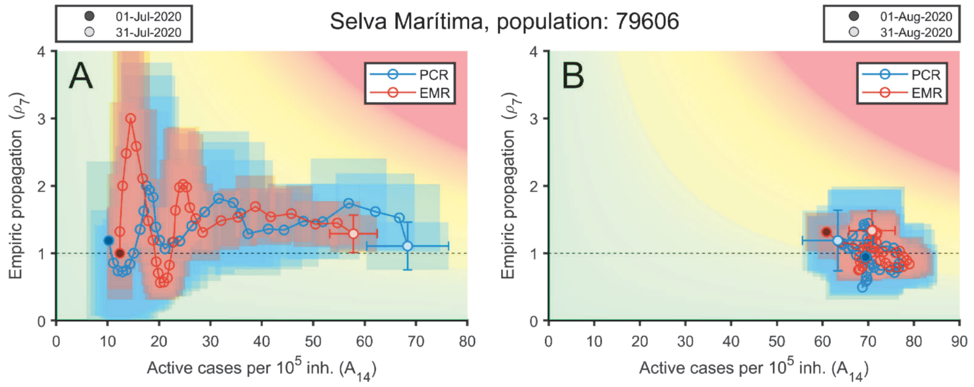

Fig. 122 Risk diagram for the evolution of the COVID-19 pandemic in Selva Marítima based on EMR (red) and on PCR (blue) cases for the month of July (left) and August 2020 (right).

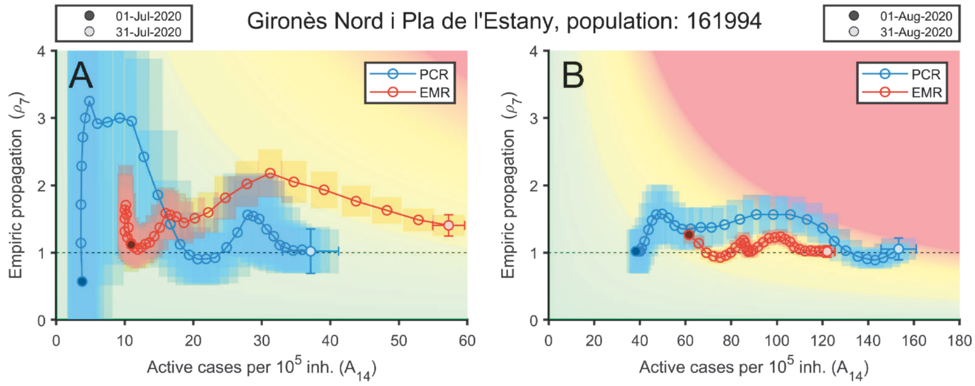

Fig. 123 Risk diagram for the evolution of the COVID-19 pandemic in Gironès Nord i Pla de l'Estany based on EMR (red) and on PCR (blue) cases for the month of July (left) and August 2020 (right).

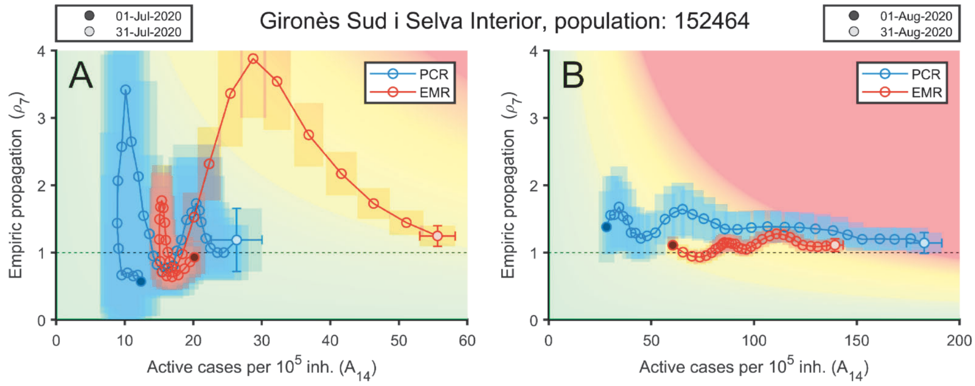

Fig. 124 Risk diagram for the evolution of the COVID-19 pandemic in Gironès Sud i Selva Interior based on EMR (red) and on PCR (blue) cases for the month of July (left) and August 2020 (right).

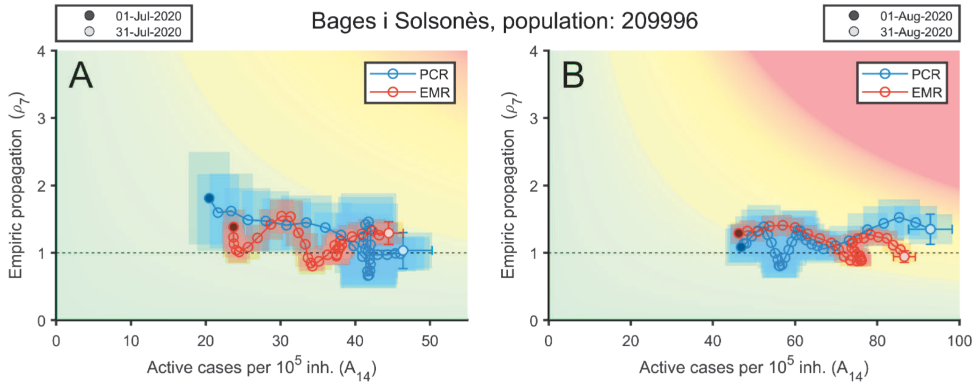

Fig. 125 Risk diagram for the evolution of the COVID-19 pandemic in Bages i Solsonès based on EMR (red) and on PCR (blue) cases for the month of July (left) and August 2020 (right).

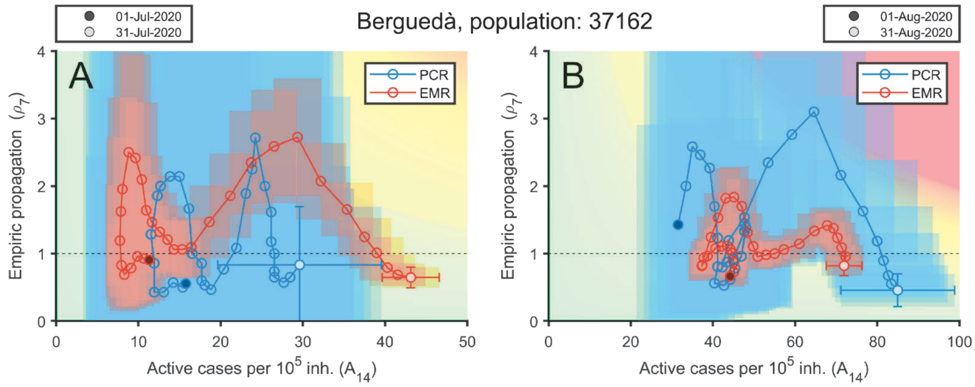

Fig. 126 Risk diagram for the evolution of the COVID-19 pandemic in Berguedà based on EMR (red) and on PCR (blue) cases for the month of July (left) and August 2020 (right).

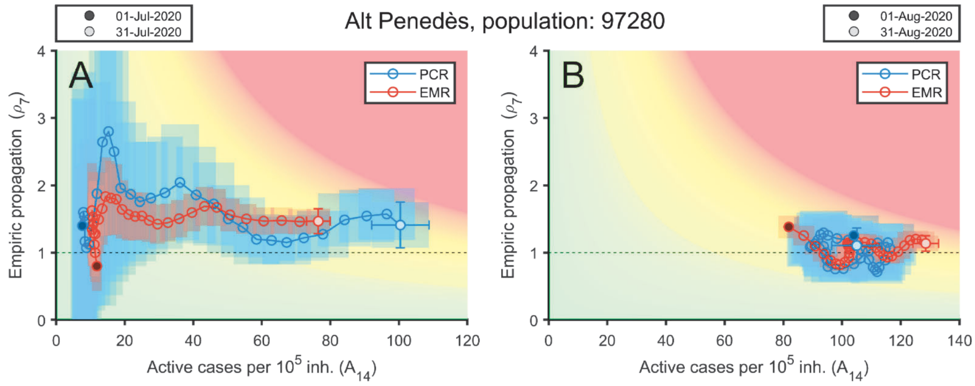

Fig. 127 Risk diagram for the evolution of the COVID-19 pandemic in Alt Penedès based on EMR (red) and on PCR (blue) cases for the month of July (left) and August 2020 (right).

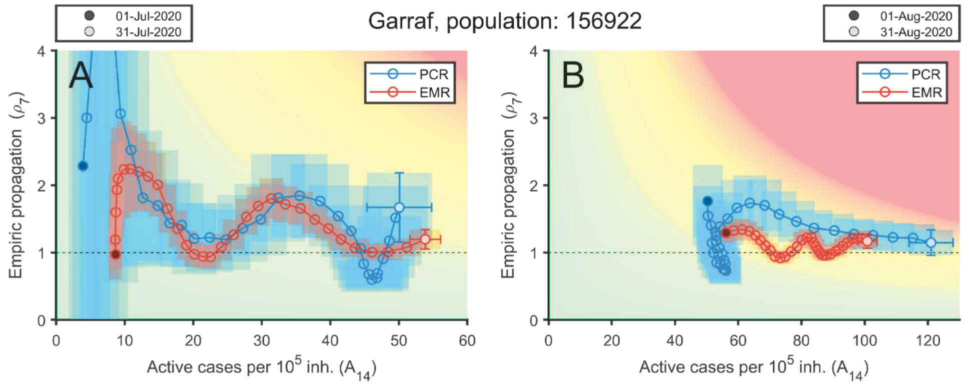

Fig. 128 Risk diagram for the evolution of the COVID-19 pandemic in Garraf based on EMR (red) and on PCR (blue) cases for the month of July (left) and August 2020 (right).

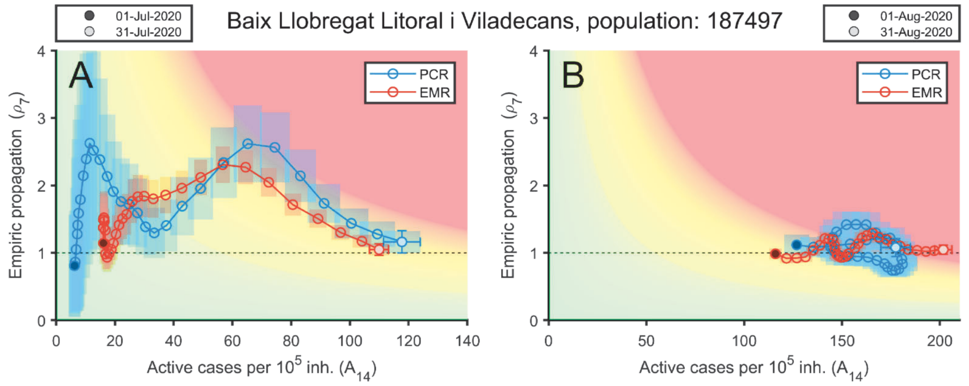

Fig. 129 Risk diagram for the evolution of the COVID-19 pandemic in Baix Llobregat Litoral i Viladecans based on EMR (red) and on PCR (blue) cases for the month of July (left) and August 2020 (right).

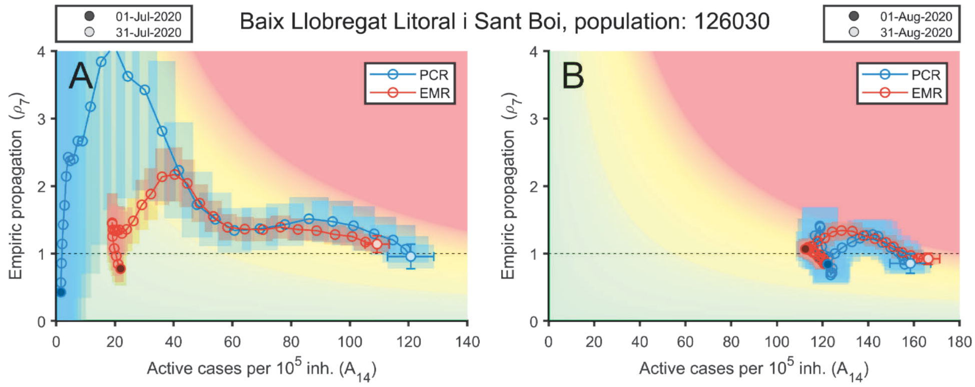

Fig. 130 Risk diagram for the evolution of the COVID-19 pandemic in Baix Llobregat Litoral i Sant Boi based on EMR (red) and on PCR (blue) cases for the month of July (left) and August 2020 (right).

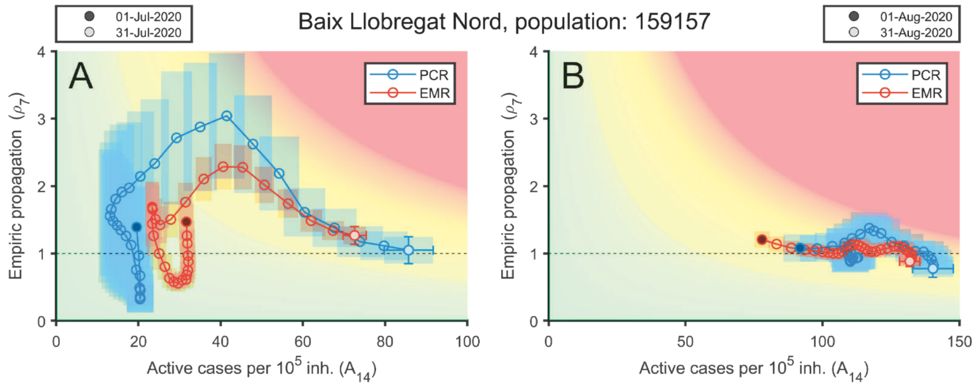

Fig. 131 Risk diagram for the evolution of the COVID-19 pandemic in Baix Llobregat Nord based on EMR (red) and on PCR (blue) cases for the month of July (left) and August 2020 (right).

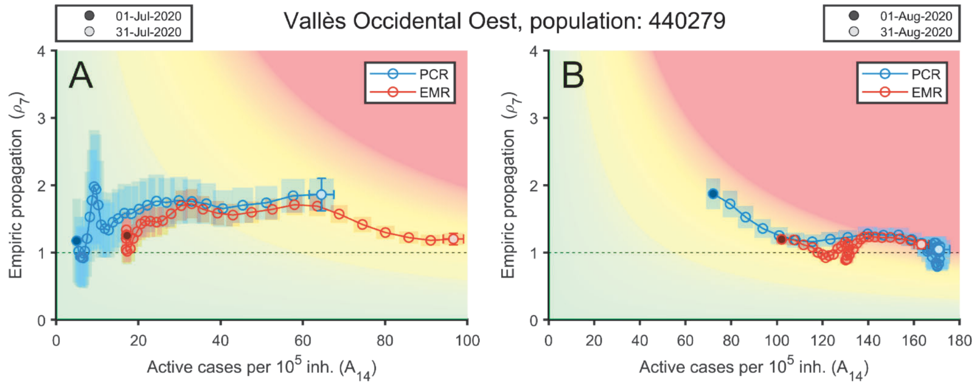

Fig. 132 Risk diagram for the evolution of the COVID-19 pandemic in Vallès Occidental Oest based on EMR (red) and on PCR (blue) cases for the month of July (left) and August 2020 (right).

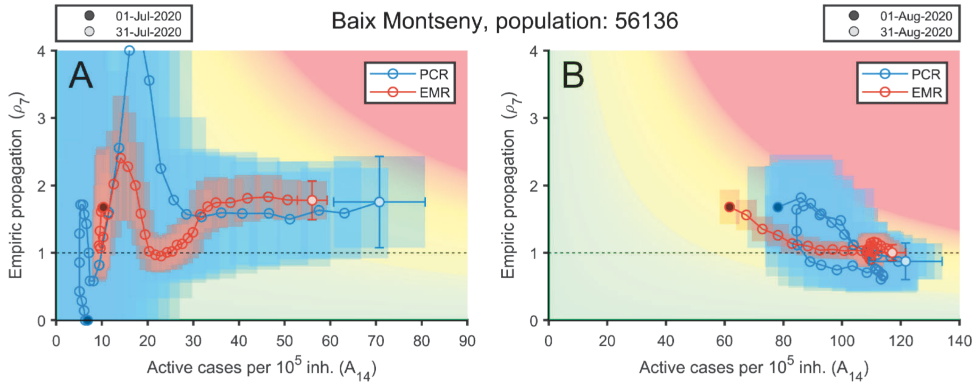

Fig. 133 Risk diagram for the evolution of the COVID-19 pandemic in Baix Montseny based on EMR (red) and on PCR (blue) cases for the month of July (left) and August 2020 (right).

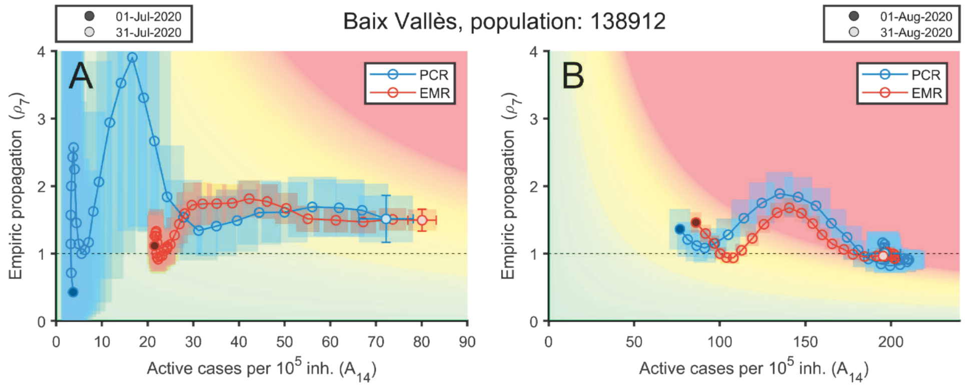

Fig. 134 Risk diagram for the evolution of the COVID-19 pandemic in Baix Vallès based on EMR (red) and on PCR (blue) cases for the month of July (left) and August 2020 (right).

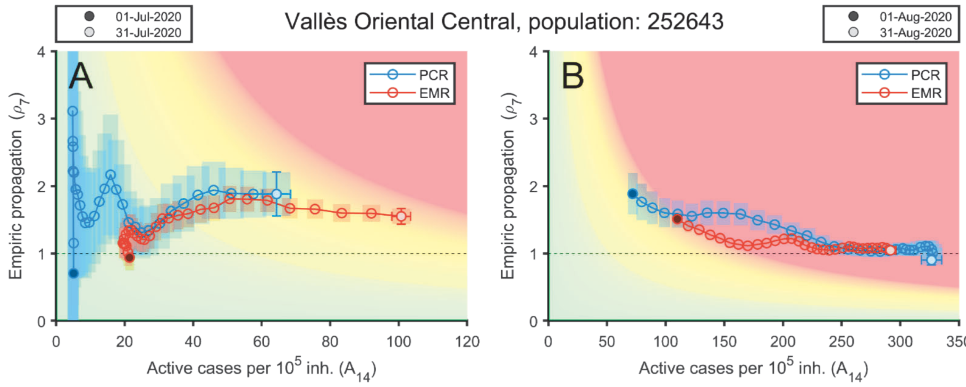

Fig. 135 Risk diagram for the evolution of the COVID-19 pandemic in Vallès Oriental Central based on EMR (red) and on PCR (blue) cases for the month of July (left) and August 2020 (right).

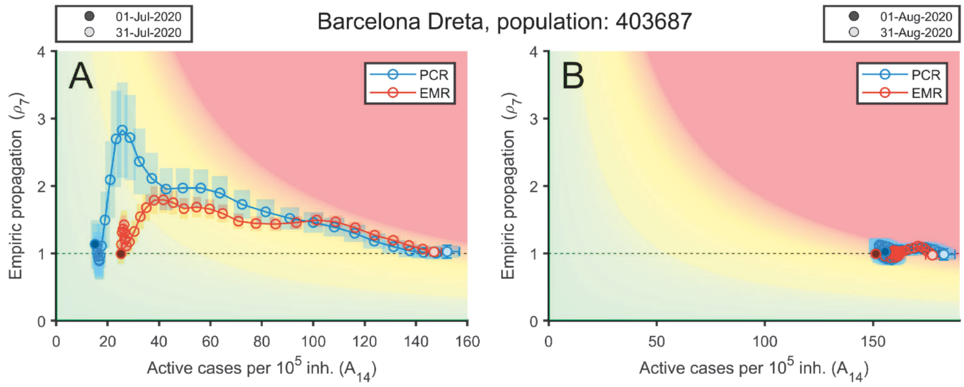

Fig. 136 Risk diagram for the evolution of the COVID-19 pandemic in Barcelona Dreta based on EMR (red) and on PCR (blue) cases for the month of July (left) and August 2020 (right).

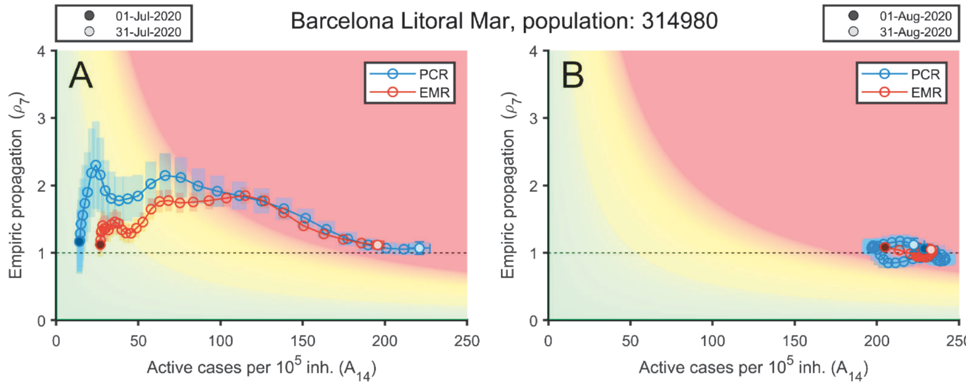

Fig. 137 Risk diagram for the evolution of the COVID-19 pandemic in Barcelona Litoral Mar based on EMR (red) and on PCR (blue) cases for the month of July (left) and August 2020 (right).

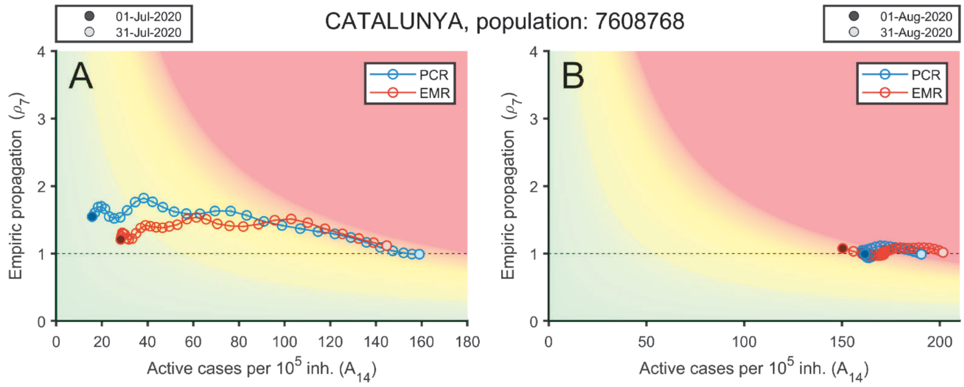

Fig. 138 Risk diagram for the evolution of the COVID-19 pandemic in CATALUNYA based on EMR (red) and on PCR (blue) cases for the month of July (left) and August 2020 (right).

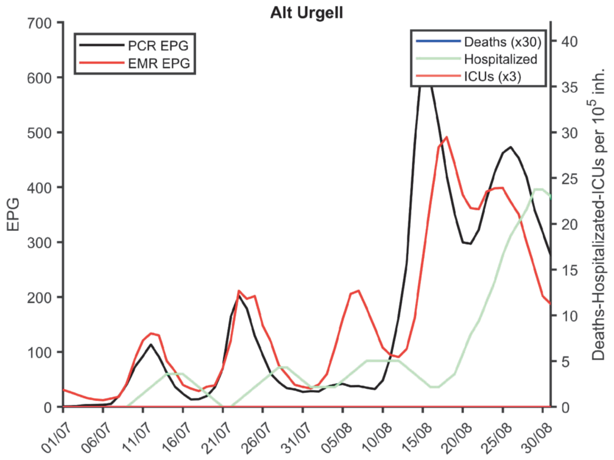

Fig. 139 Daily measures of EMR (maroon) and PCR-based EPGs (black), number of hospitalizations (green), ICU occupancy (red) and mortality (blue) in Alt Urgell over time from late June until the end of August 2020. Hospitalizations, ICU and mortality are averaged over the previous 7 day period.

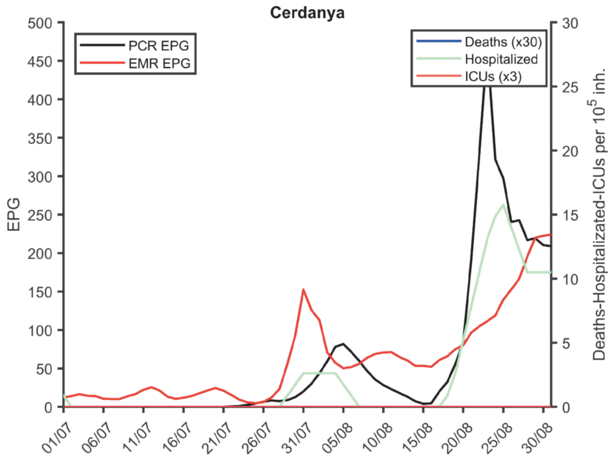

Fig. 140 Daily measures of EMR (maroon) and PCR-based EPGs (black), number of hospitalizations (green), ICU occupancy (red) and mortality (blue) in Cerdanya over time from late June until the end of August 2020. Hospitalizations, ICU and mortality are averaged over the previous 7 day period.

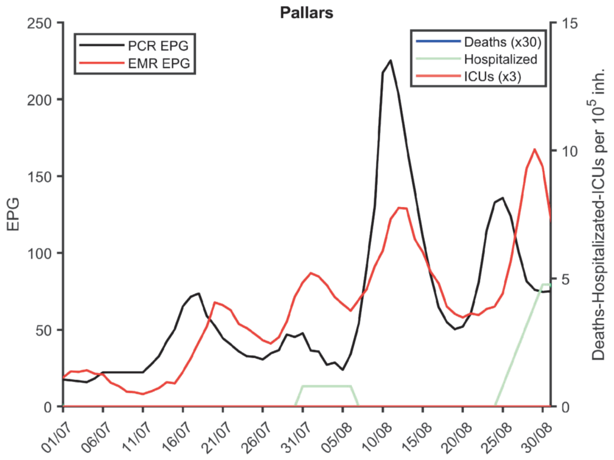

Fig. 141 Daily measures of EMR (maroon) and PCR-based EPGs (black), number of hospitalizations (green), ICU occupancy (red) and mortality (blue) in Pallars over time from late June until the end of August 2020. Hospitalizations, ICU and mortality are averaged over the previous 7 day period.

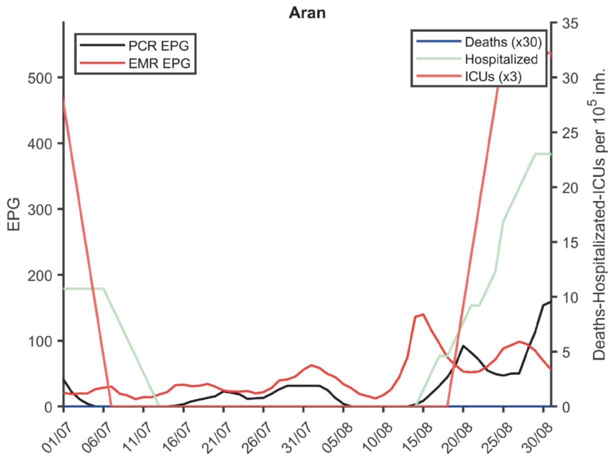

Fig. 142 Daily measures of EMR (maroon) and PCR-based EPGs (black), number of hospitalizations (green), ICU occupancy (red) and mortality (blue) in Aran over time from late June until the end of August 2020. Hospitalizations, ICU and mortality are averaged over the previous 7 day period.

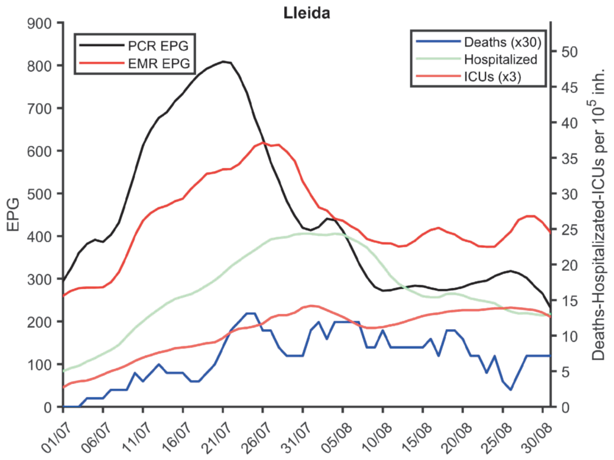

Fig. 143 Daily measures of EMR (maroon) and PCR-based EPGs (black), number of hospitalizations (green), ICU occupancy (red) and mortality (blue) in Lleida over time from late June until the end of August 2020. Hospitalizations, ICU and mortality are averaged over the previous 7 day period.

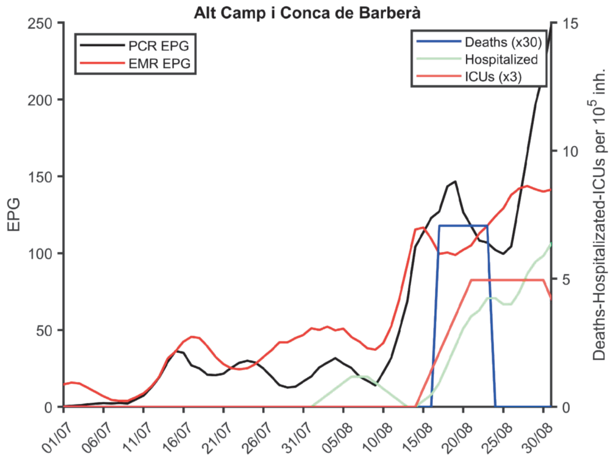

Fig. 144 Daily measures of EMR (maroon) and PCR-based EPGs (black), number of hospitalizations (green), ICU occupancy (red) and mortality (blue) in Alt Camp i Conca de Barberà over time from late June until the end of August 2020. Hospitalizations, ICU and mortality are averaged over the previous 7 day period.

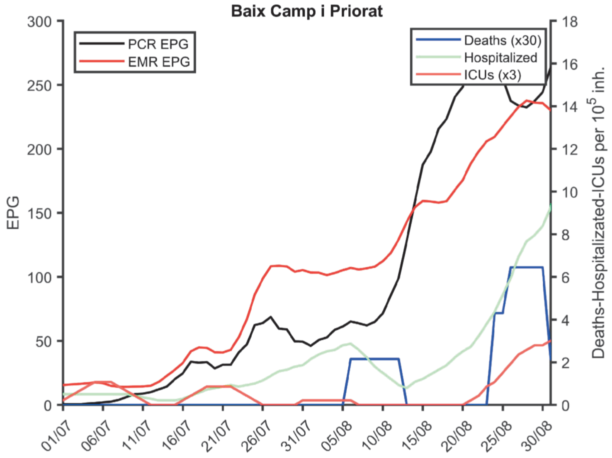

Fig. 145 Daily measures of EMR (maroon) and PCR-based EPGs (black), number of hospitalizations (green), ICU occupancy (red) and mortality (blue) in Baix Camp i Priorat over time from late June until the end of August 2020. Hospitalizations, ICU and mortality are averaged over the previous 7 day period.

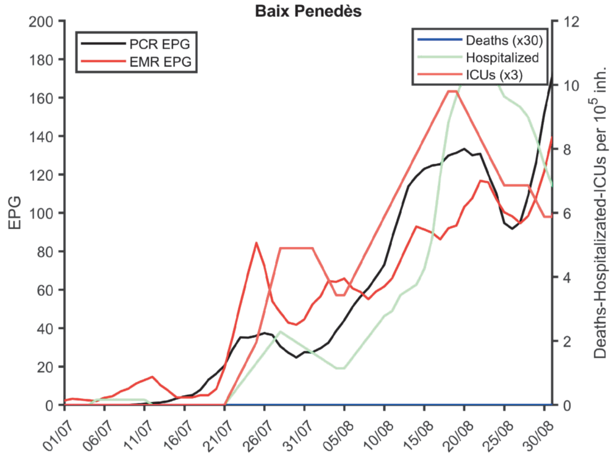

Fig. 146 Daily measures of EMR (maroon) and PCR-based EPGs (black), number of hospitalizations (green), ICU occupancy (red) and mortality (blue) in Baix Penedès over time from late June until the end of August 2020. Hospitalizations, ICU and mortality are averaged over the previous 7 day period.

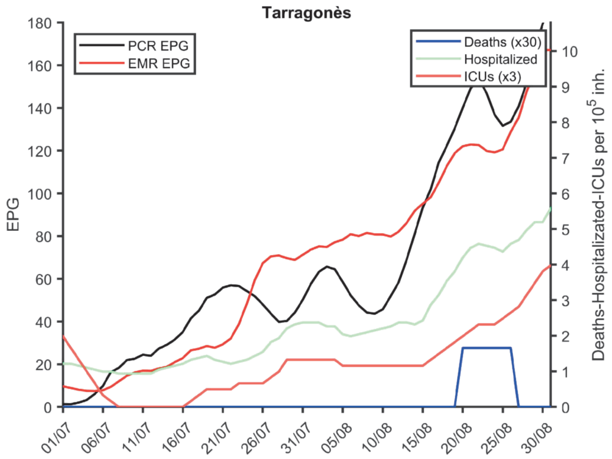

Fig. 147 Daily measures of EMR (maroon) and PCR-based EPGs (black), number of hospitalizations (green), ICU occupancy (red) and mortality (blue) in Tarragonès over time from late June until the end of August 2020. Hospitalizations, ICU and mortality are averaged over the previous 7 day period.

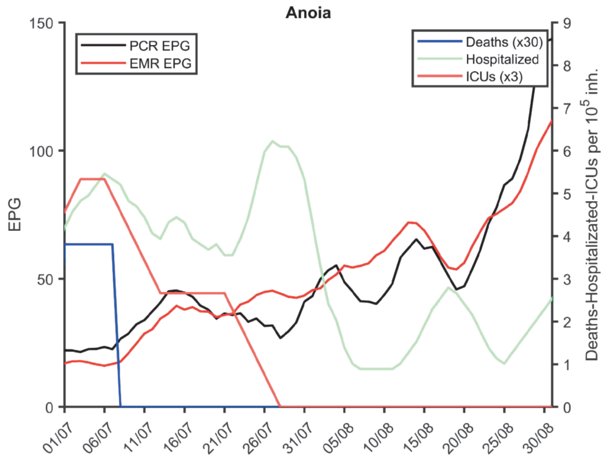

Fig. 148 Daily measures of EMR (maroon) and PCR-based EPGs (black), number of hospitalizations (green), ICU occupancy (red) and mortality (blue) in Anoia over time from late June until the end of August 2020. Hospitalizations, ICU and mortality are averaged over the previous 7 day period.

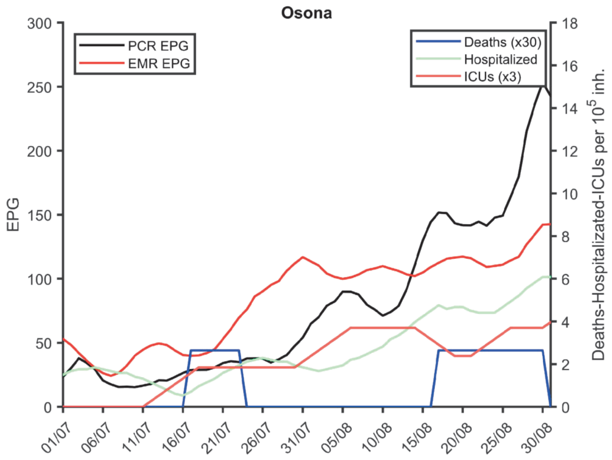

Fig. 149 Daily measures of EMR (maroon) and PCR-based EPGs (black), number of hospitalizations (green), ICU occupancy (red) and mortality (blue) in Osona over time from late June until the end of August 2020. Hospitalizations, ICU and mortality are averaged over the previous 7 day period.

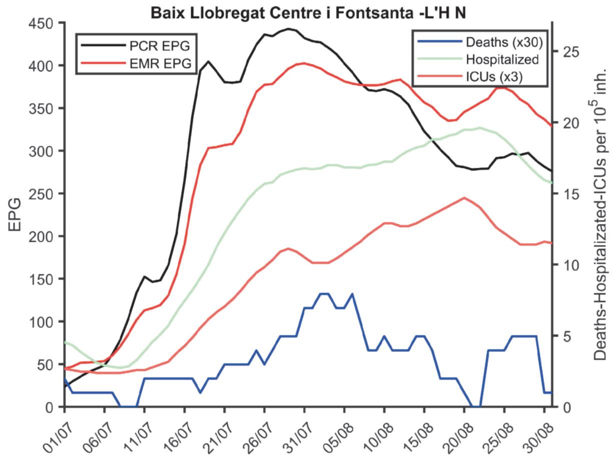

Fig. 150 Daily measures of EMR (maroon) and PCR-based EPGs (black), number of hospitalizations (green), ICU occupancy (red) and mortality (blue) in Baix Llobregat Centre i Fontsanta -L'H N over time from late June until the end of August 2020. Hospitalizations, ICU and mortality are averaged over the previous 7 day period.

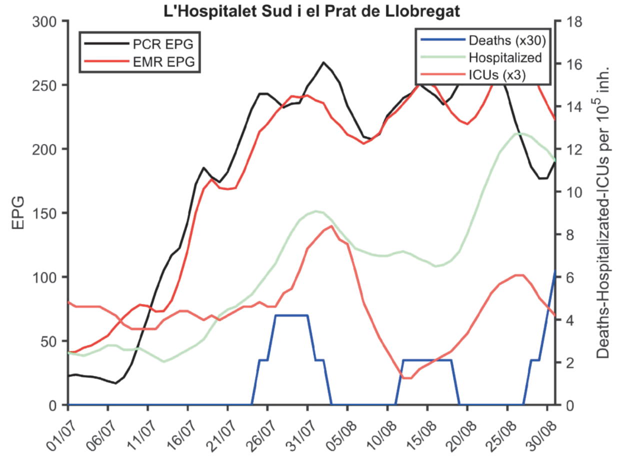

Fig. 151 Daily measures of EMR (maroon) and PCR-based EPGs (black), number of hospitalizations (green), ICU occupancy (red) and mortality (blue) in L'Hospitalet Sud i el Prat de Llobregat over time from late June until the end of August 2020. Hospitalizations, ICU and mortality are averaged over the previous 7 day period.

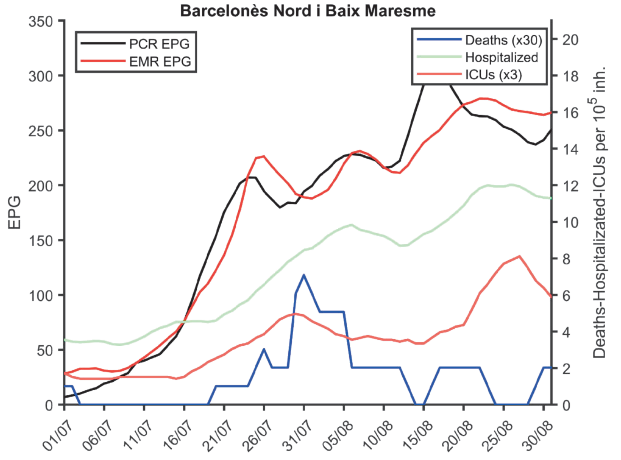

Fig. 152 Daily measures of EMR (maroon) and PCR-based EPGs (black), number of hospitalizations (green), ICU occupancy (red) and mortality (blue) in Barcelonès Nord i Baix Maresme over time from late June until the end of August 2020. Hospitalizations, ICU and mortality are averaged over the previous 7 day period.

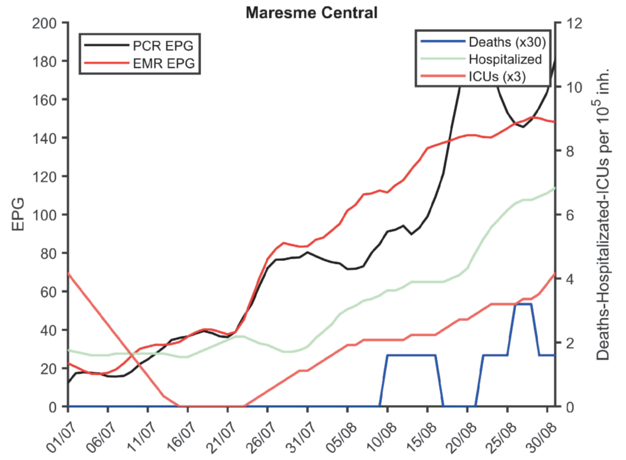

Fig. 153 Daily measures of EMR (maroon) and PCR-based EPGs (black), number of hospitalizations (green), ICU occupancy (red) and mortality (blue) in Maresme Central over time from late June until the end of August 2020. Hospitalizations, ICU and mortality are averaged over the previous 7 day period.

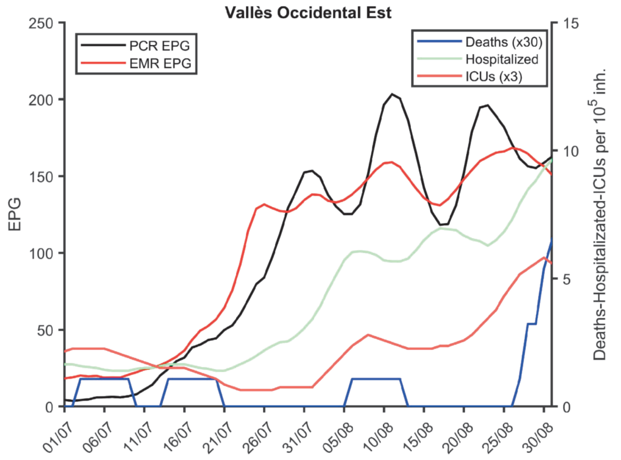

Fig. 154 Daily measures of EMR (maroon) and PCR-based EPGs (black), number of hospitalizations (green), ICU occupancy (red) and mortality (blue) in Vallès Occidental Est over time from late June until the end of August 2020. Hospitalizations, ICU and mortality are averaged over the previous 7 day period.

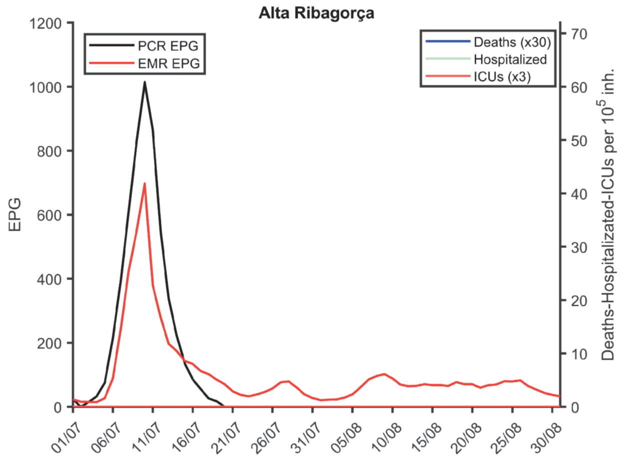

Fig. 155 Daily measures of EMR (maroon) and PCR-based EPGs (black), number of hospitalizations (green), ICU occupancy (red) and mortality (blue) in Alta Ribagorça over time from late June until the end of August 2020. Hospitalizations, ICU and mortality are averaged over the previous 7 day period.

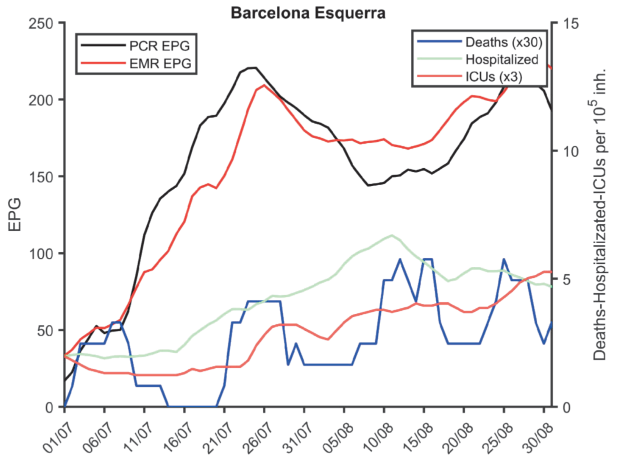

Fig. 156 Daily measures of EMR (maroon) and PCR-based EPGs (black), number of hospitalizations (green), ICU occupancy (red) and mortality (blue) in Barcelona Esquerra over time from late June until the end of August 2020. Hospitalizations, ICU and mortality are averaged over the previous 7 day period.

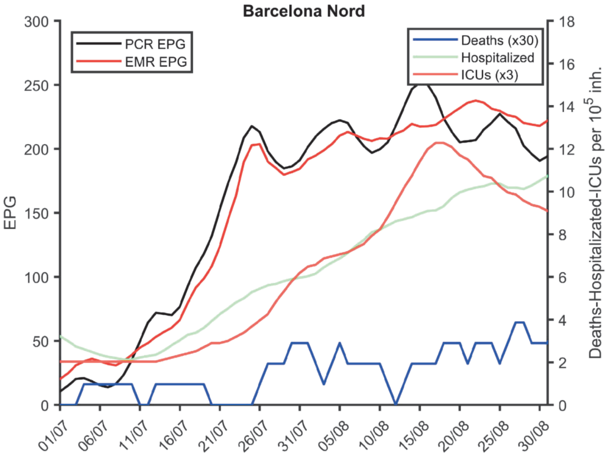

Fig. 157 Daily measures of EMR (maroon) and PCR-based EPGs (black), number of hospitalizations (green), ICU occupancy (red) and mortality (blue) in Barcelona Nord over time from late June until the end of August 2020. Hospitalizations, ICU and mortality are averaged over the previous 7 day period.

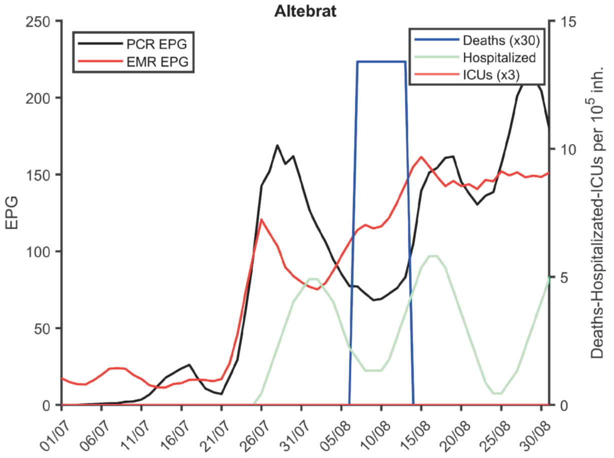

Fig. 158 Daily measures of EMR (maroon) and PCR-based EPGs (black), number of hospitalizations (green), ICU occupancy (red) and mortality (blue) in Altebrat over time from late June until the end of August 2020. Hospitalizations, ICU and mortality are averaged over the previous 7 day period.

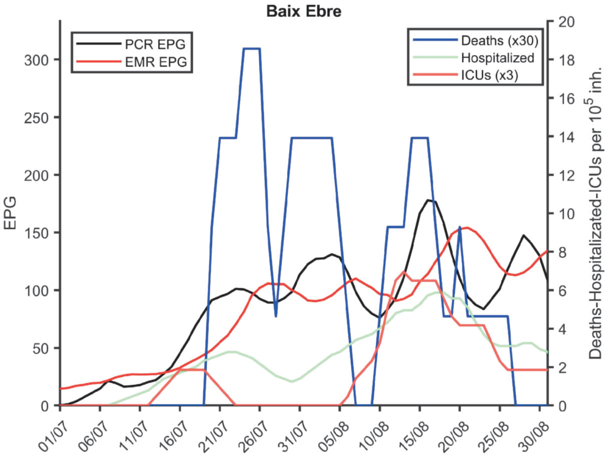

Fig. 159 Daily measures of EMR (maroon) and PCR-based EPGs (black), number of hospitalizations (green), ICU occupancy (red) and mortality (blue) in Baix Ebre over time from late June until the end of August 2020. Hospitalizations, ICU and mortality are averaged over the previous 7 day period.

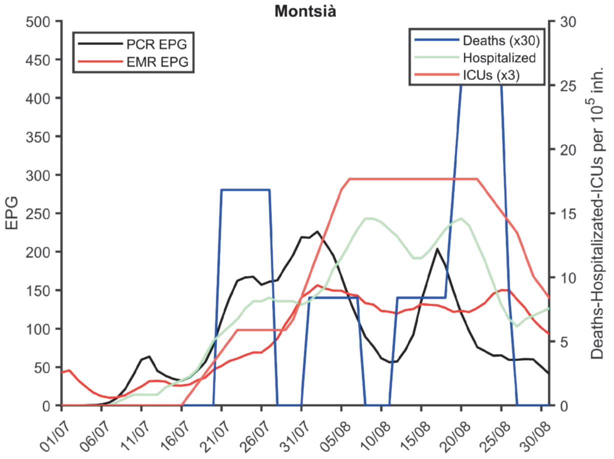

Fig. 160 Daily measures of EMR (maroon) and PCR-based EPGs (black), number of hospitalizations (green), ICU occupancy (red) and mortality (blue) in Montsià over time from late June until the end of August 2020. Hospitalizations, ICU and mortality are averaged over the previous 7 day period.

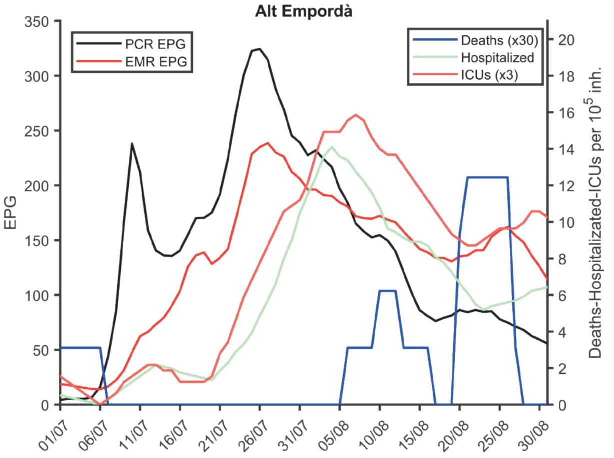

Fig. 161 Daily measures of EMR (maroon) and PCR-based EPGs (black), number of hospitalizations (green), ICU occupancy (red) and mortality (blue) in Alt Empordà over time from late June until the end of August 2020. Hospitalizations, ICU and mortality are averaged over the previous 7 day period.

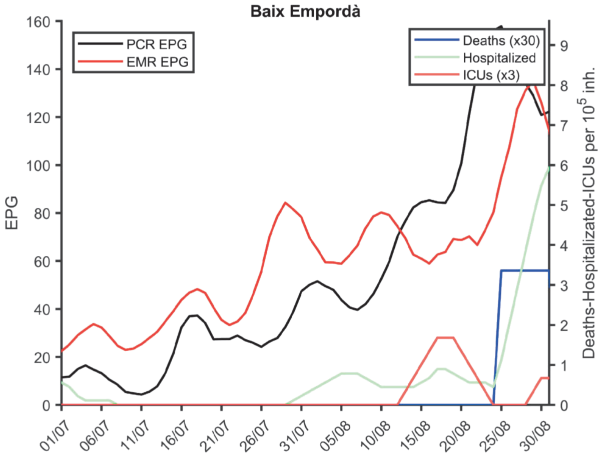

Fig. 162 Daily measures of EMR (maroon) and PCR-based EPGs (black), number of hospitalizations (green), ICU occupancy (red) and mortality (blue) in Baix Empordà over time from late June until the end of August 2020. Hospitalizations, ICU and mortality are averaged over the previous 7 day period.

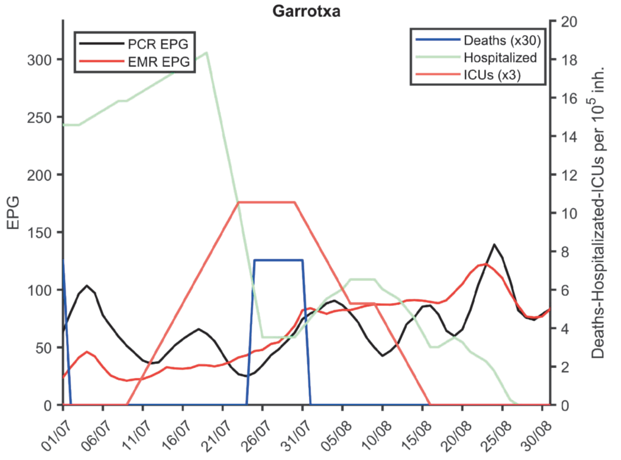

Fig. 163 Daily measures of EMR (maroon) and PCR-based EPGs (black), number of hospitalizations (green), ICU occupancy (red) and mortality (blue) in Garrotxa over time from late June until the end of August 2020. Hospitalizations, ICU and mortality are averaged over the previous 7 day period.

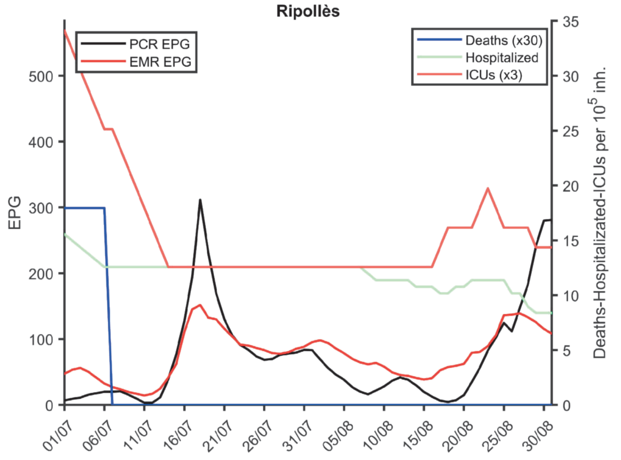

Fig. 164 Daily measures of EMR (maroon) and PCR-based EPGs (black), number of hospitalizations (green), ICU occupancy (red) and mortality (blue) in Ripollès over time from late June until the end of August 2020. Hospitalizations, ICU and mortality are averaged over the previous 7 day period.

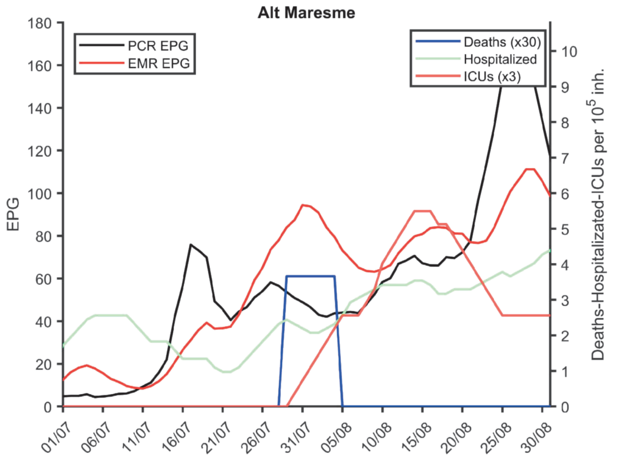

Fig. 165 Daily measures of EMR (maroon) and PCR-based EPGs (black), number of hospitalizations (green), ICU occupancy (red) and mortality (blue) in Alt Maresme over time from late June until the end of August 2020. Hospitalizations, ICU and mortality are averaged over the previous 7 day period.

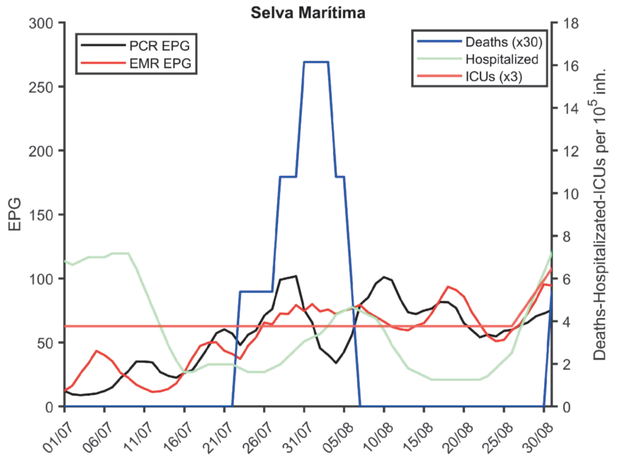

Fig. 166 Daily measures of EMR (maroon) and PCR-based EPGs (black), number of hospitalizations (green), ICU occupancy (red) and mortality (blue) in Selva Marítima over time from late June until the end of August 2020. Hospitalizations, ICU and mortality are averaged over the previous 7 day period.

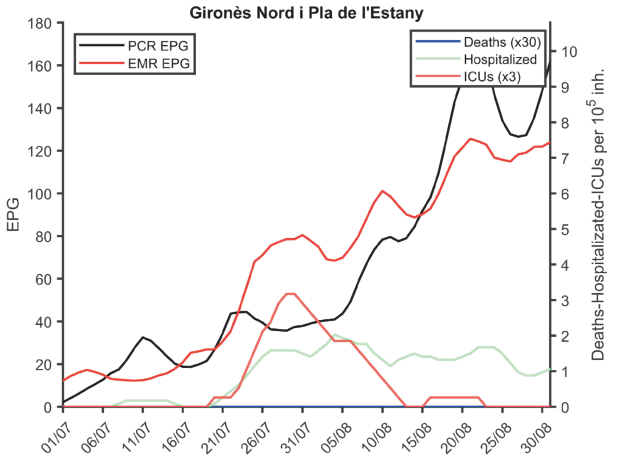

Fig. 167 Daily measures of EMR (maroon) and PCR-based EPGs (black), number of hospitalizations (green), ICU occupancy (red) and mortality (blue) in Gironès Nord i Pla de l'Estany over time from late June until the end of August 2020. Hospitalizations, ICU and mortality are averaged over the previous 7 day period.

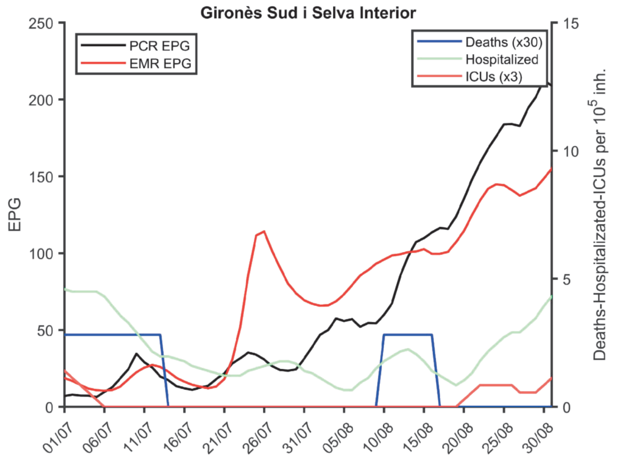

Fig. 168 Daily measures of EMR (maroon) and PCR-based EPGs (black), number of hospitalizations (green), ICU occupancy (red) and mortality (blue) in Gironès Sud i Selva Interior over time from late June until the end of August 2020. Hospitalizations, ICU and mortality are averaged over the previous 7 day period.

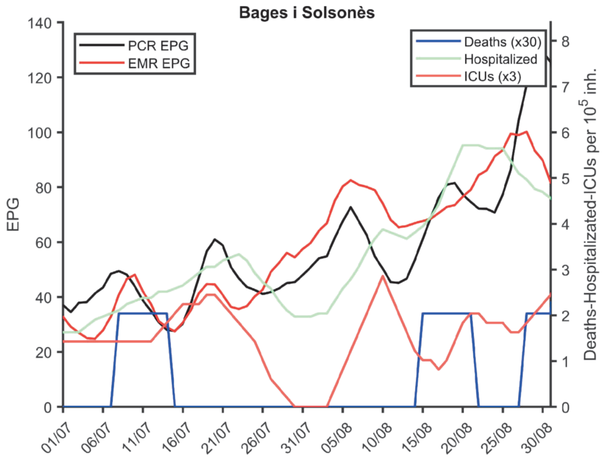

Fig. 169 Daily measures of EMR (maroon) and PCR-based EPGs (black), number of hospitalizations (green), ICU occupancy (red) and mortality (blue) in Bages i Solsonès over time from late June until the end of August 2020. Hospitalizations, ICU and mortality are averaged over the previous 7 day period.

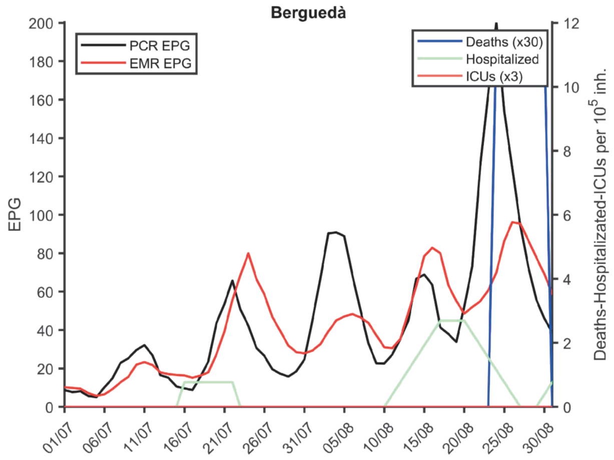

Fig. 170 Daily measures of EMR (maroon) and PCR-based EPGs (black), number of hospitalizations (green), ICU occupancy (red) and mortality (blue) in Berguedà over time from late June until the end of August 2020. Hospitalizations, ICU and mortality are averaged over the previous 7 day period.

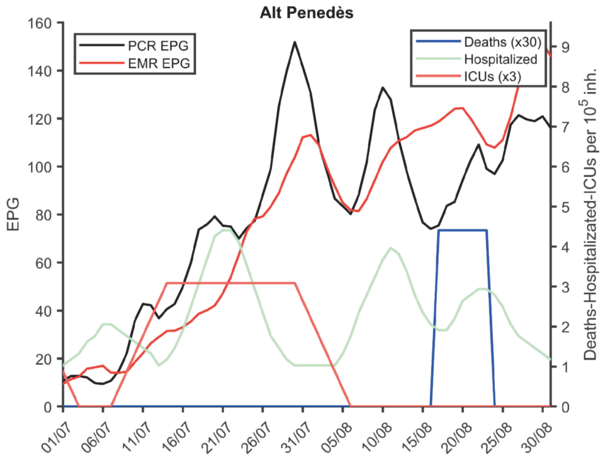

Fig. 171 Daily measures of EMR (maroon) and PCR-based EPGs (black), number of hospitalizations (green), ICU occupancy (red) and mortality (blue) in Alt Penedès over time from late June until the end of August 2020. Hospitalizations, ICU and mortality are averaged over the previous 7 day period.

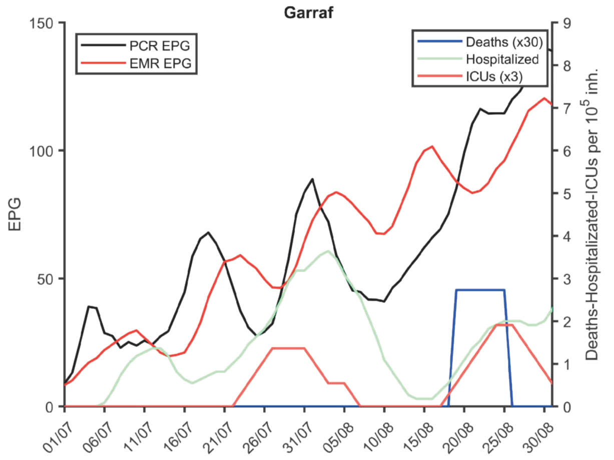

Fig. 172 Daily measures of EMR (maroon) and PCR-based EPGs (black), number of hospitalizations (green), ICU occupancy (red) and mortality (blue) in Garraf over time from late June until the end of August 2020. Hospitalizations, ICU and mortality are averaged over the previous 7 day period.

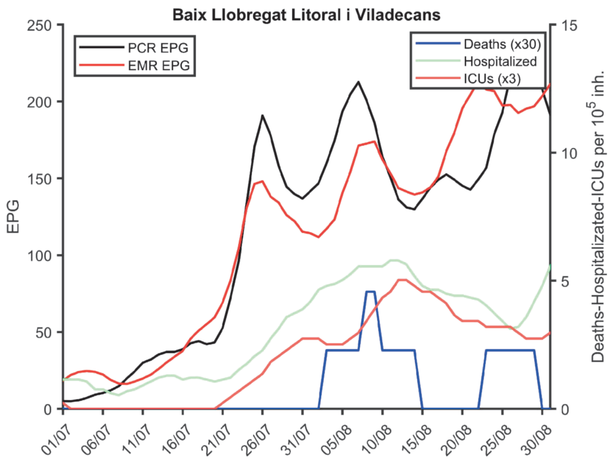

Fig. 173 Daily measures of EMR (maroon) and PCR-based EPGs (black), number of hospitalizations (green), ICU occupancy (red) and mortality (blue) in Baix Llobregat Litoral i Viladecans over time from late June until the end of August 2020. Hospitalizations, ICU and mortality are averaged over the previous 7 day period.

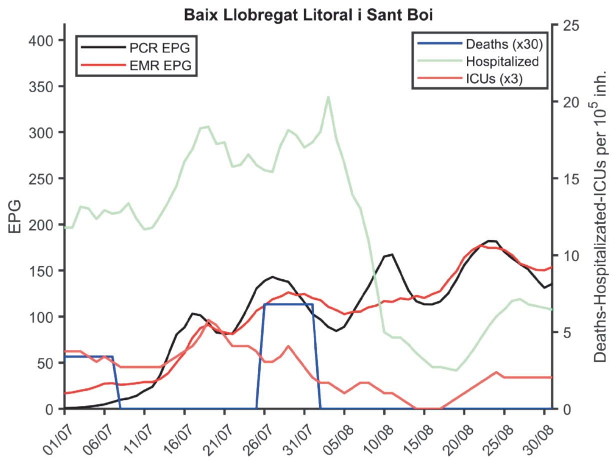

Fig. 174 Daily measures of EMR (maroon) and PCR-based EPGs (black), number of hospitalizations (green), ICU occupancy (red) and mortality (blue) in Baix Llobregat Litoral i Sant Boi over time from late June until the end of August 2020. Hospitalizations, ICU and mortality are averaged over the previous 7 day period.

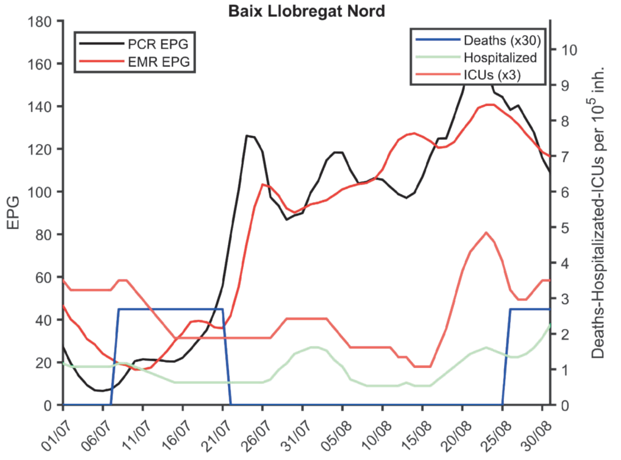

Fig. 175 Daily measures of EMR (maroon) and PCR-based EPGs (black), number of hospitalizations (green), ICU occupancy (red) and mortality (blue) in Baix Llobregat Nord over time from late June until the end of August 2020. Hospitalizations, ICU and mortality are averaged over the previous 7 day period.

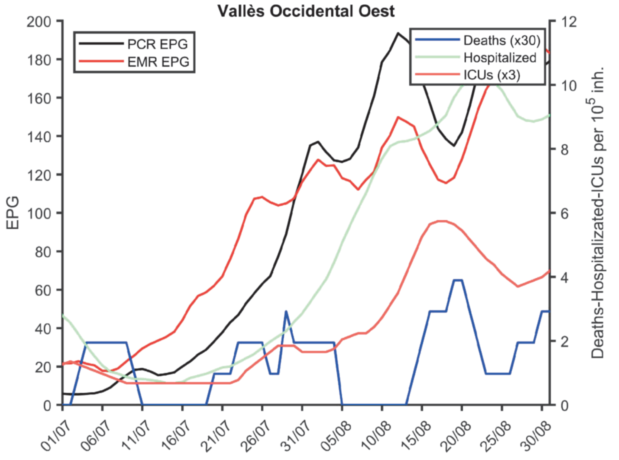

Fig. 176 Daily measures of EMR (maroon) and PCR-based EPGs (black), number of hospitalizations (green), ICU occupancy (red) and mortality (blue) in Vallès Occidental Oest over time from late June until the end of August 2020. Hospitalizations, ICU and mortality are averaged over the previous 7 day period.

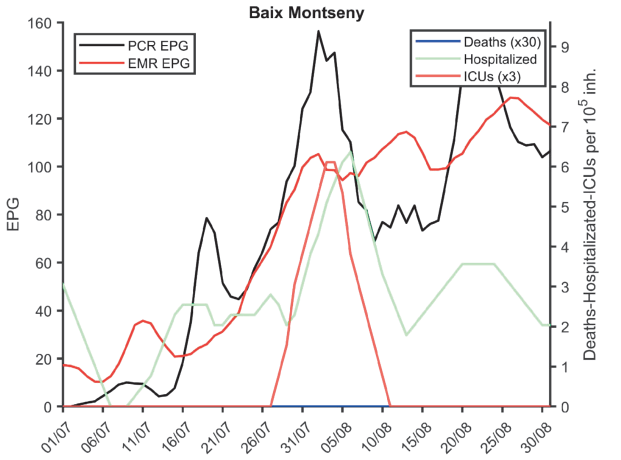

Fig. 177 Daily measures of EMR (maroon) and PCR-based EPGs (black), number of hospitalizations (green), ICU occupancy (red) and mortality (blue) in Baix Montseny over time from late June until the end of August 2020. Hospitalizations, ICU and mortality are averaged over the previous 7 day period.

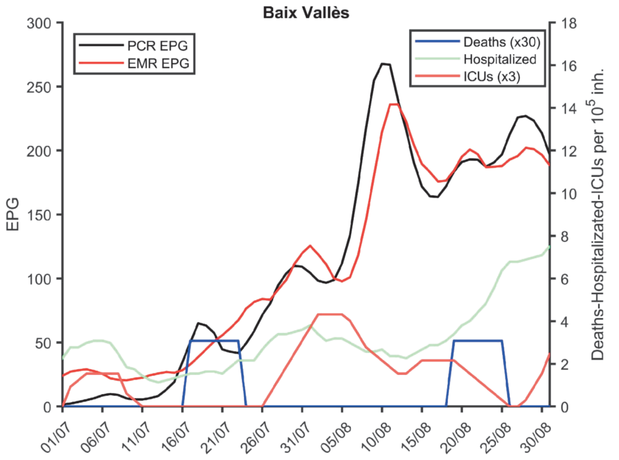

Fig. 178 Daily measures of EMR (maroon) and PCR-based EPGs (black), number of hospitalizations (green), ICU occupancy (red) and mortality (blue) in Baix Vallès over time from late June until the end of August 2020. Hospitalizations, ICU and mortality are averaged over the previous 7 day period.

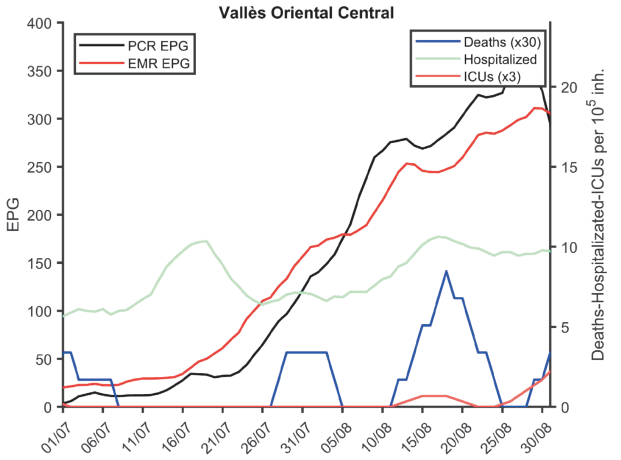

Fig. 179 Daily measures of EMR (maroon) and PCR-based EPGs (black), number of hospitalizations (green), ICU occupancy (red) and mortality (blue) in Vallès Oriental Central over time from late June until the end of August 2020. Hospitalizations, ICU and mortality are averaged over the previous 7 day period.

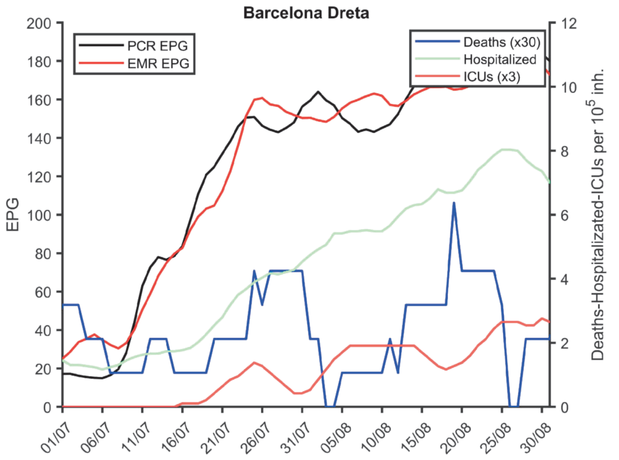

Fig. 180 Daily measures of EMR (maroon) and PCR-based EPGs (black), number of hospitalizations (green), ICU occupancy (red) and mortality (blue) in Barcelona Dreta over time from late June until the end of August 2020. Hospitalizations, ICU and mortality are averaged over the previous 7 day period.

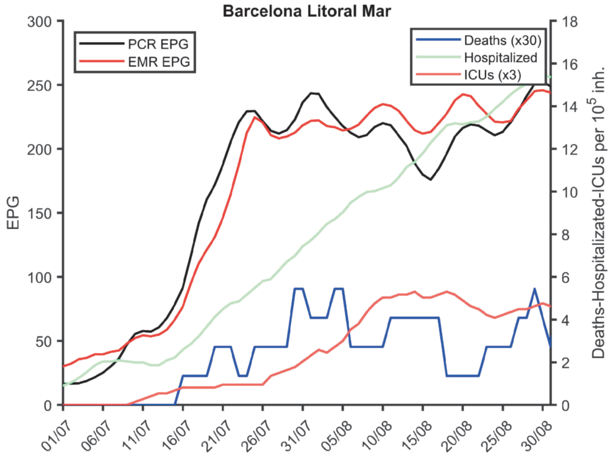

Fig. 181 Daily measures of EMR (maroon) and PCR-based EPGs (black), number of hospitalizations (green), ICU occupancy (red) and mortality (blue) in Barcelona Litoral Mar over time from late June until the end of August 2020. Hospitalizations, ICU and mortality are averaged over the previous 7 day period.

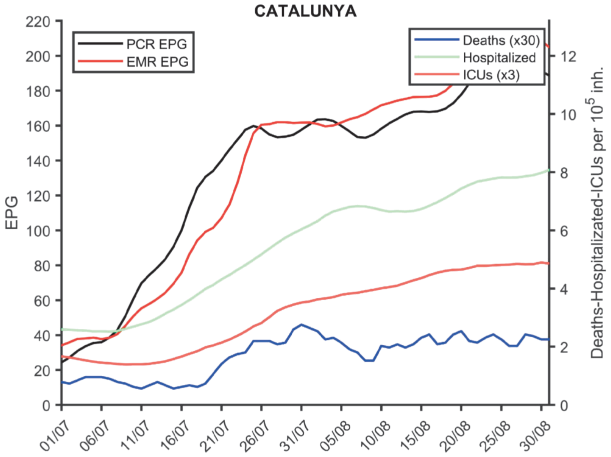

Fig. 182 Daily measures of EMR (maroon) and PCR-based EPGs (black), number of hospitalizations (green), ICU occupancy (red) and mortality (blue) in CATALUNYA over time from late June until the end of August 2020. Hospitalizations, ICU and mortality are averaged over the previous 7 day period.

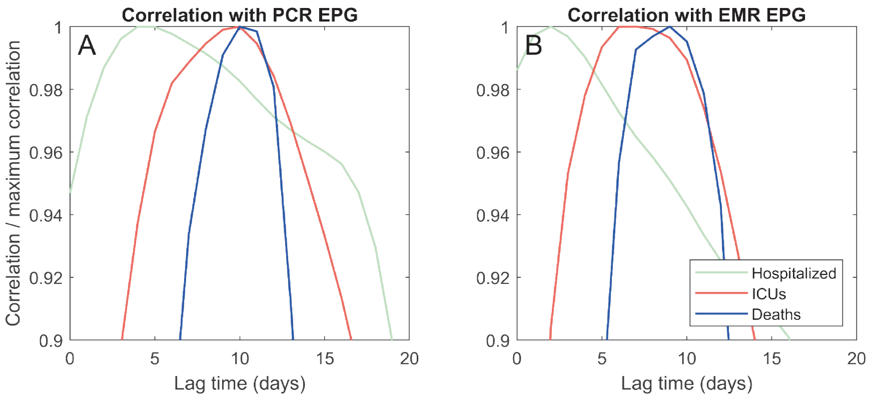

Fig. 183 Correlation between displaced series hospitalized (in green), ICUs (in red) and deaths (in blue). (A) Correlation with PCR EPG series. (B) Correlation with EMR EPG series.
